# Supplementary material for: How Can Newborn Toxicology Testing Be More Equitable? An Interactive Ethics Workshop
Source: MedEdPORTAL. 2024 Sep 10;20:11434. doi: 10.15766/mep_2374-8265.11434 (PMC11383834; doi:10.15766/mep_2374-8265.11434)
Supplement: Supplementary file 1 — Newborn Toxicology Workshop Slides.pptxParticipant Workbook.docxFacilitator Guide.docxSurvey 1.docxSurvey 2.docx [file mep_2374-8265.11434-s001.zip › A. Newborn Toxicology Workshop Slides.pptx]

## Slide 1
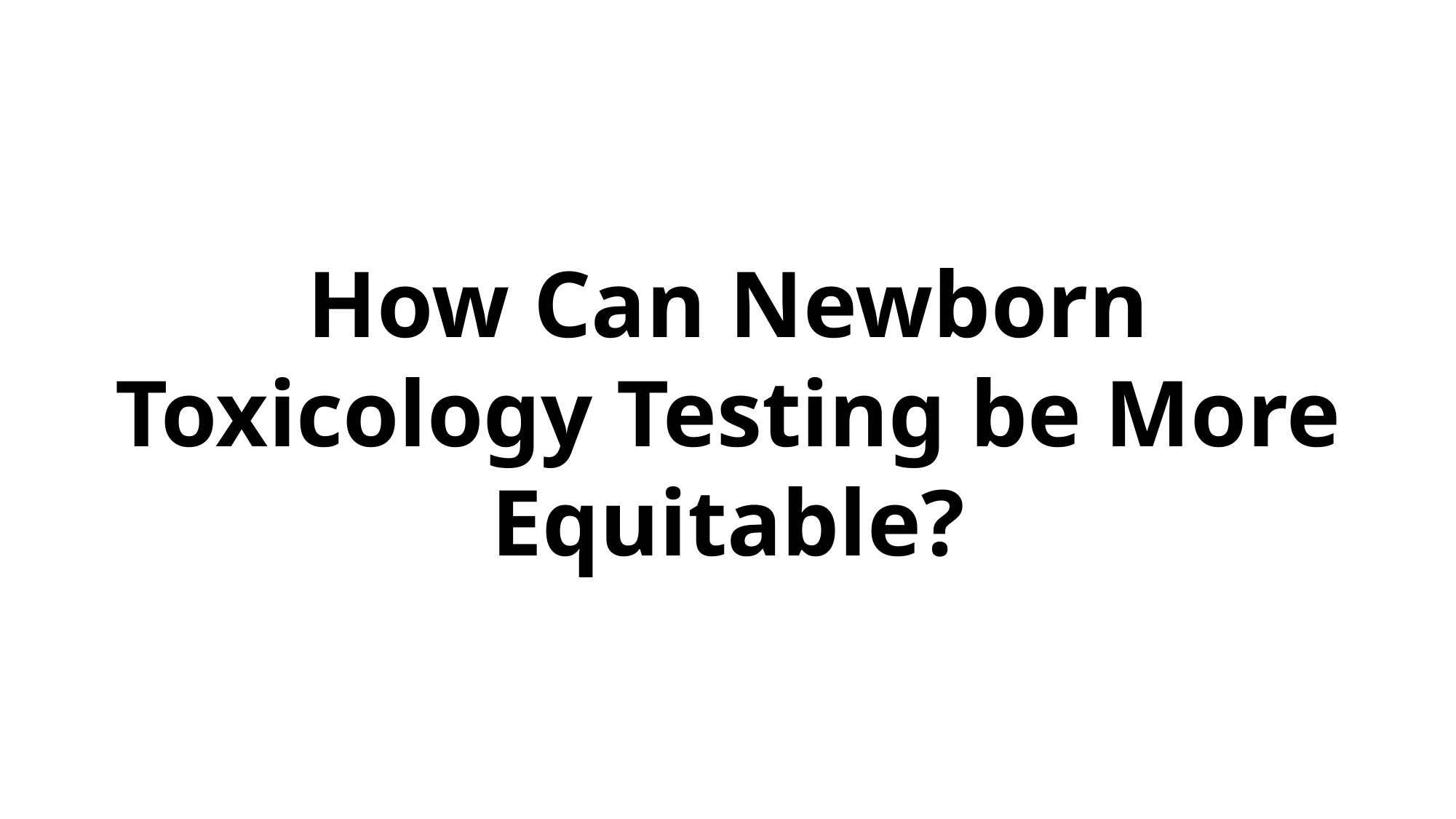

# How Can Newborn Toxicology Testing be More Equitable?

## Slide 2
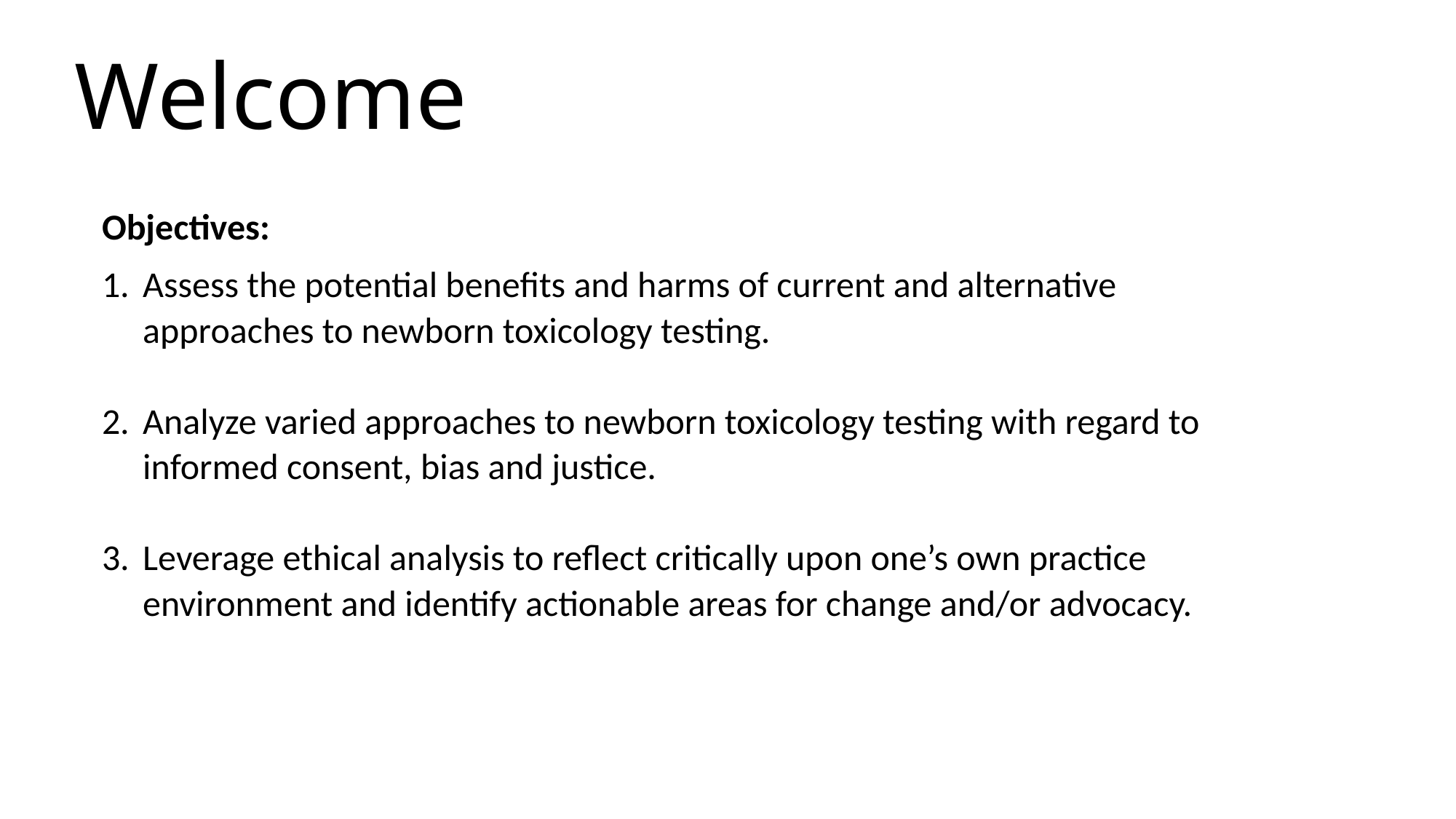

# Welcome
Objectives:
Assess the potential benefits and harms of current and alternative approaches to newborn toxicology testing.
Analyze varied approaches to newborn toxicology testing with regard to informed consent, bias and justice.
Leverage ethical analysis to reflect critically upon one’s own practice environment and identify actionable areas for change and/or advocacy.

## Slide 3
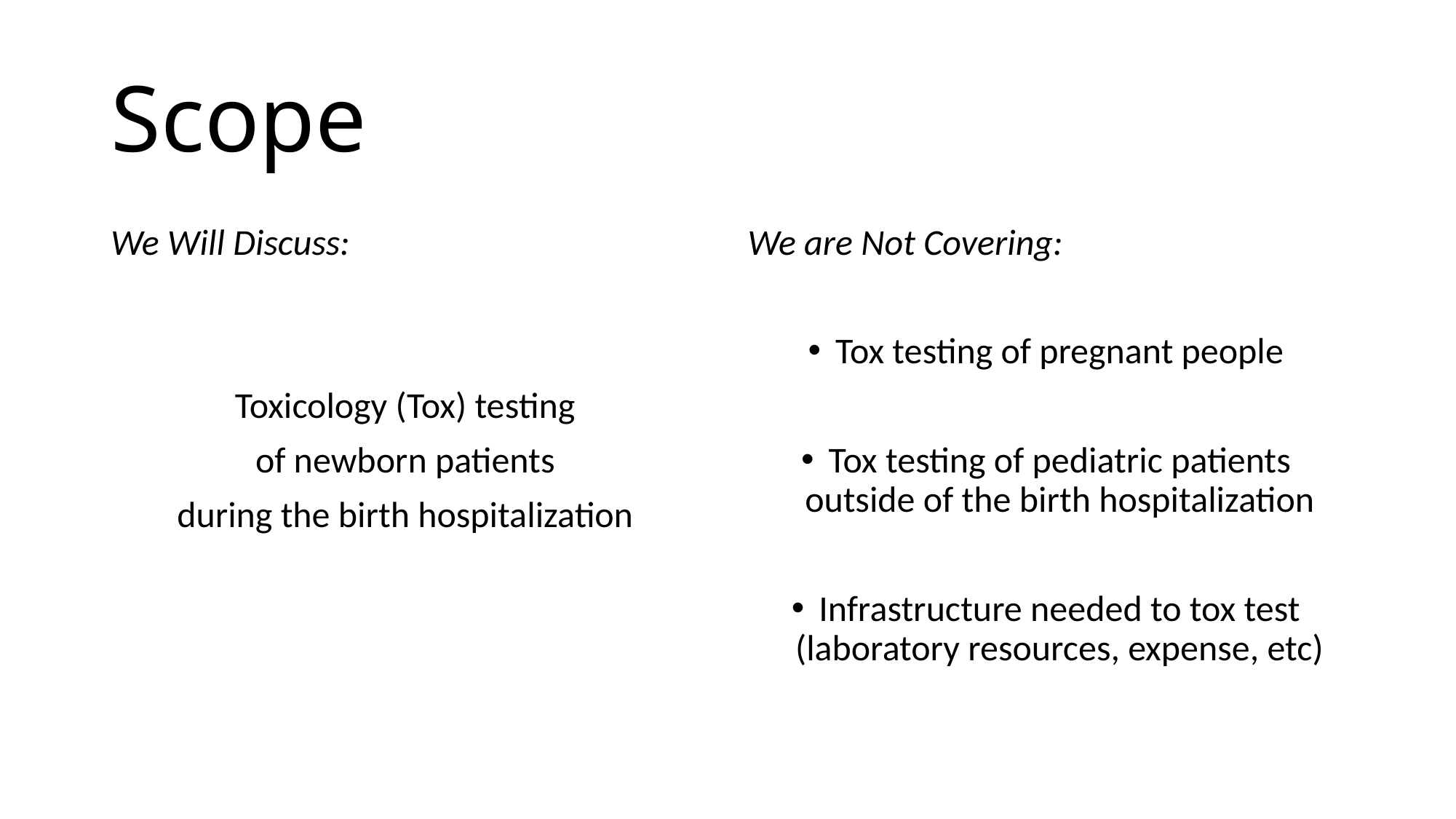

# Scope
We Will Discuss:
Toxicology (Tox) testing
of newborn patients
during the birth hospitalization
We are Not Covering:
Tox testing of pregnant people
Tox testing of pediatric patients outside of the birth hospitalization
Infrastructure needed to tox test (laboratory resources, expense, etc)

## Slide 4
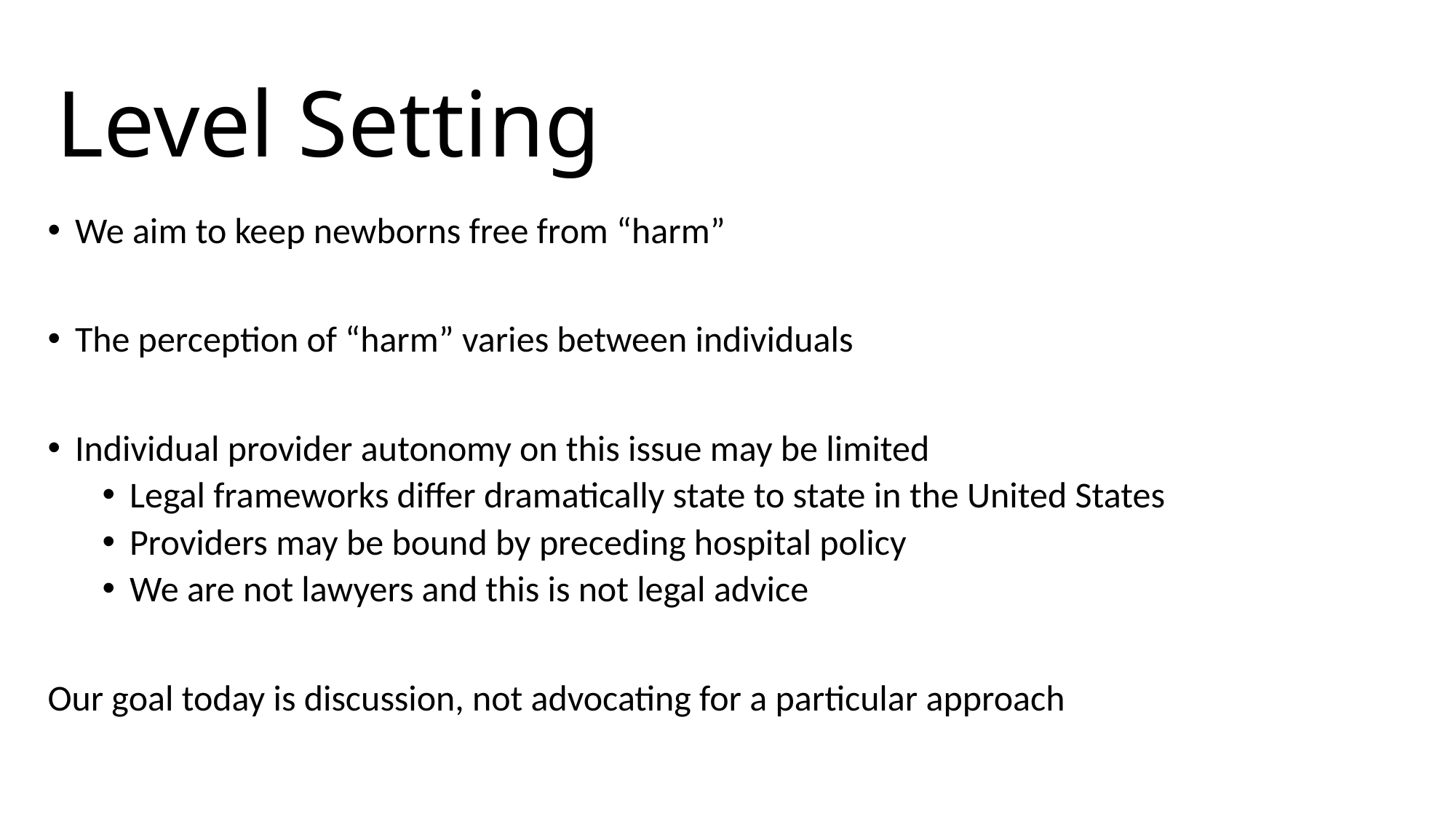

# Level Setting
We aim to keep newborns free from “harm”
The perception of “harm” varies between individuals
Individual provider autonomy on this issue may be limited
Legal frameworks differ dramatically state to state in the United States
Providers may be bound by preceding hospital policy
We are not lawyers and this is not legal advice
Our goal today is discussion, not advocating for a particular approach

## Slide 5
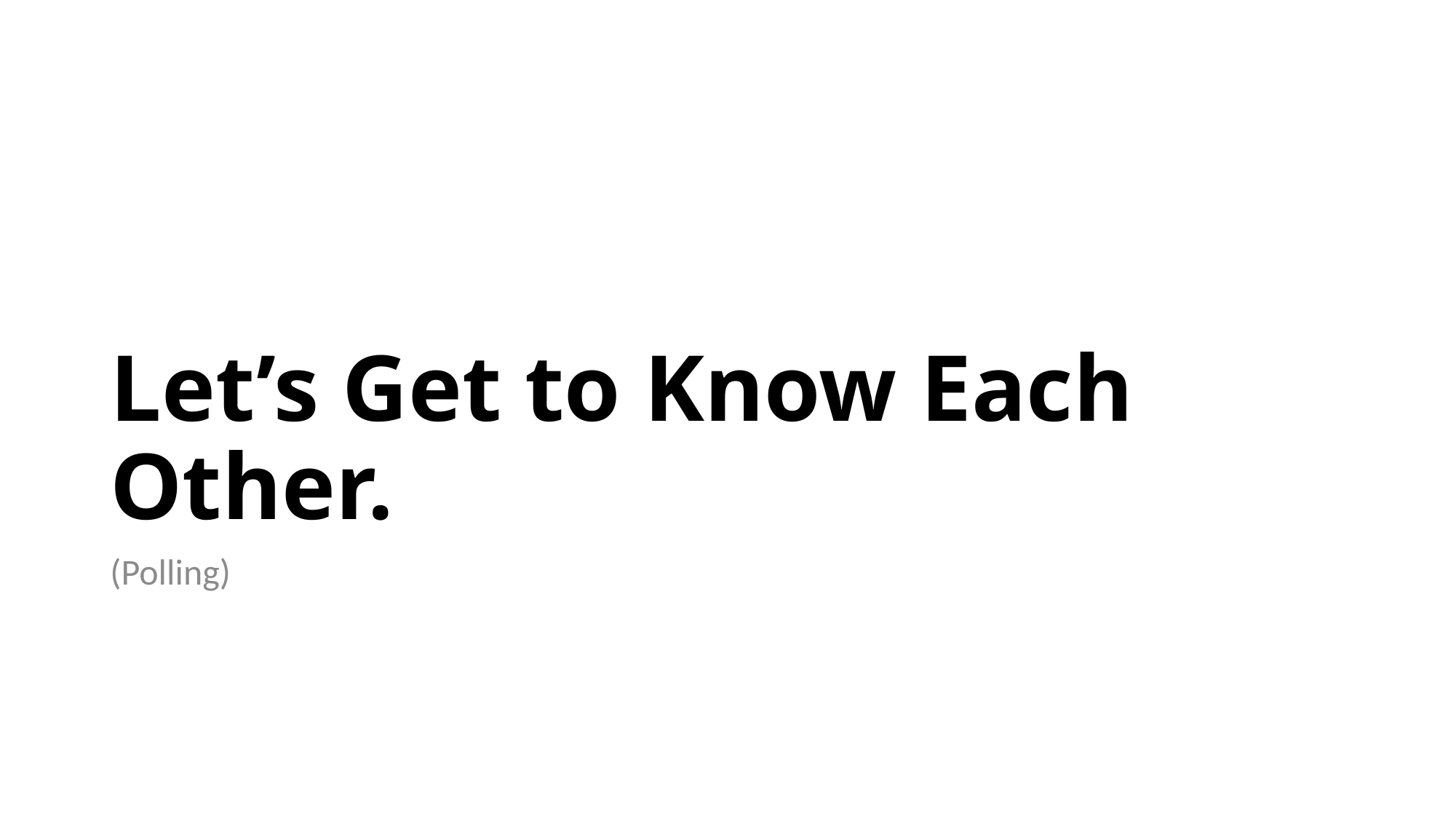

# Let’s Get to Know Each Other.
(Polling)

## Slide 6
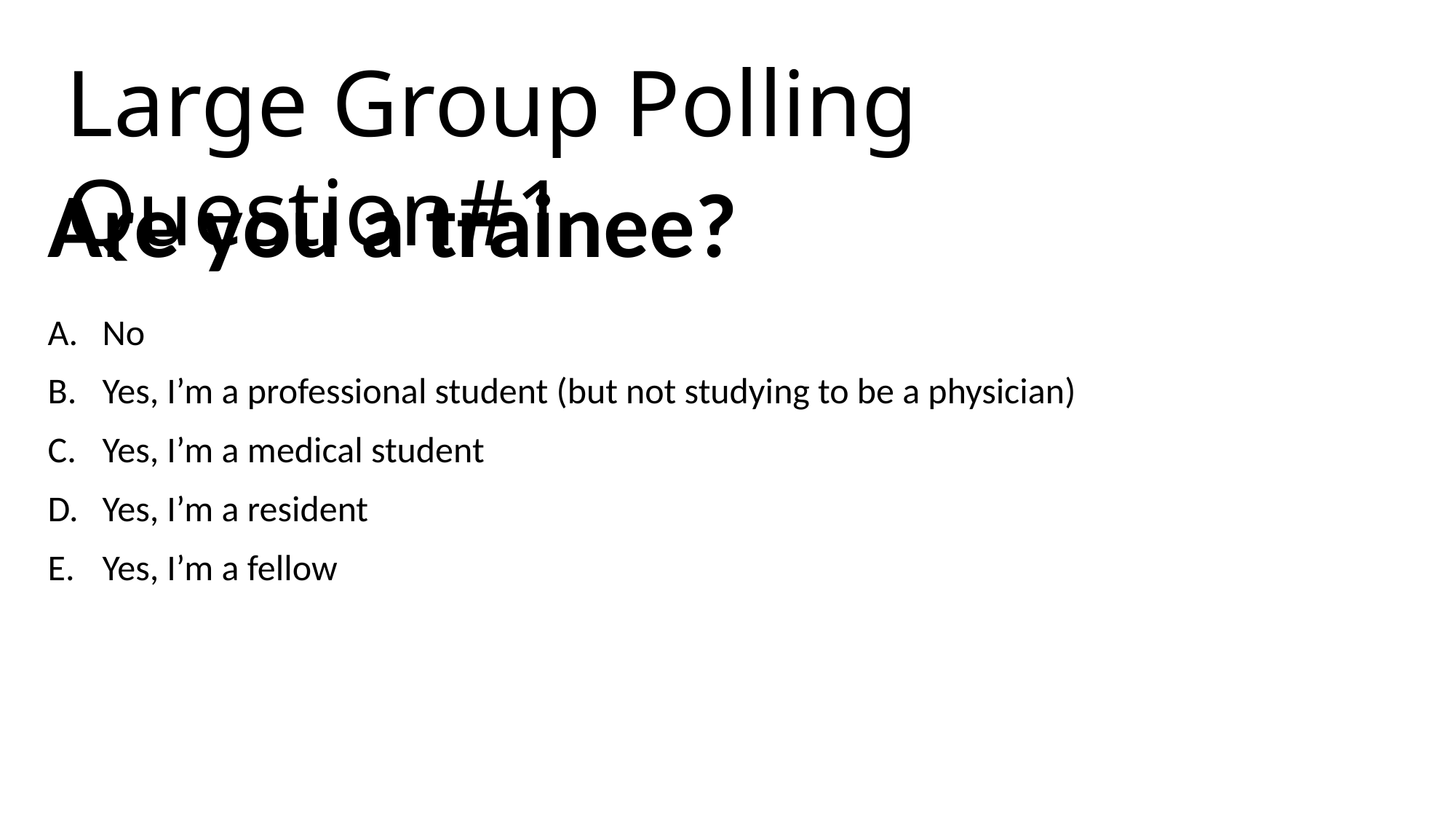

Large Group Polling Question#1
# Are you a trainee?
No
Yes, I’m a professional student (but not studying to be a physician)
Yes, I’m a medical student
Yes, I’m a resident
Yes, I’m a fellow

## Slide 7
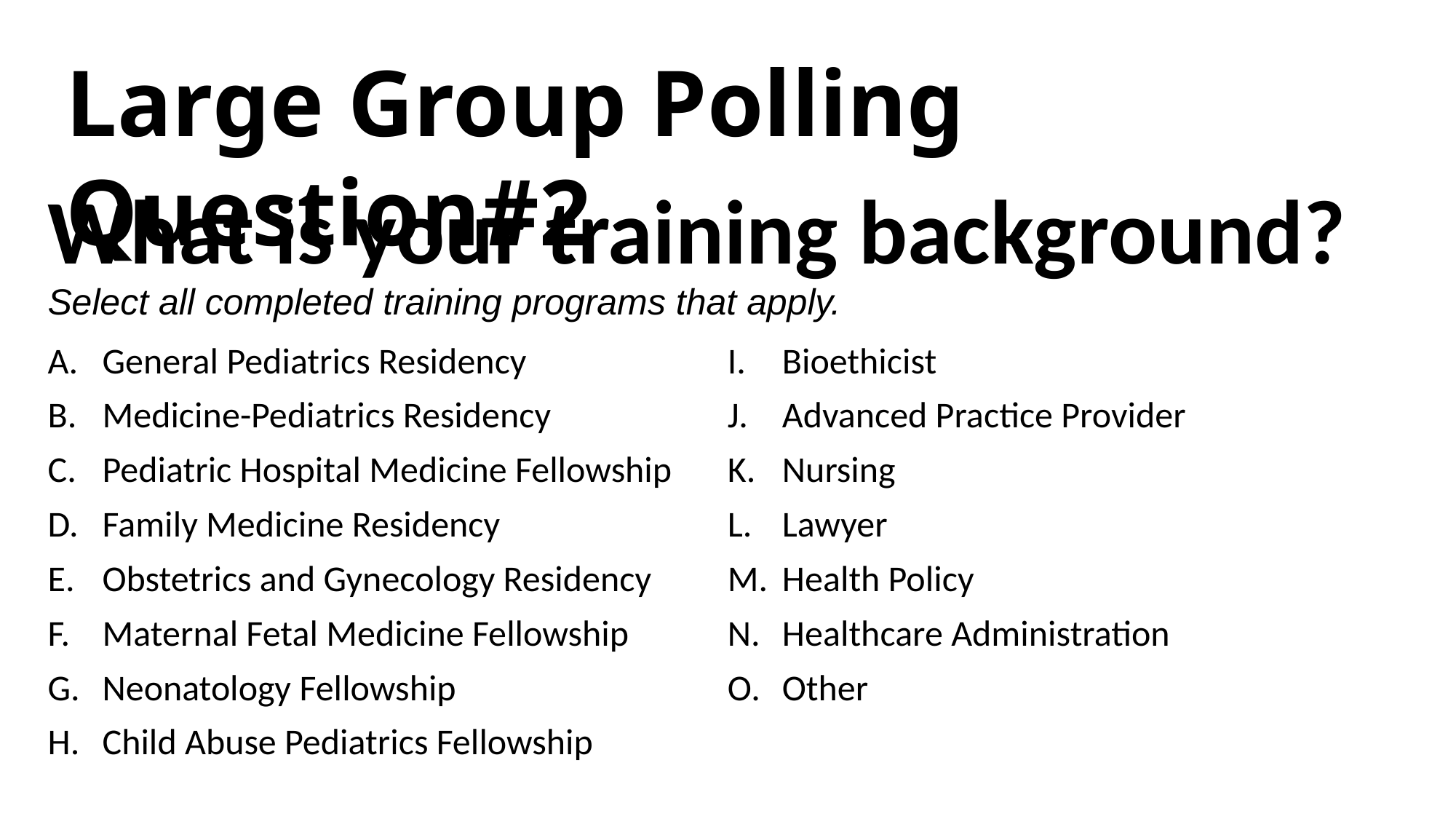

Large Group Polling Question#2
# What is your training background? Select all completed training programs that apply.
General Pediatrics Residency
Medicine-Pediatrics Residency
Pediatric Hospital Medicine Fellowship
Family Medicine Residency
Obstetrics and Gynecology Residency
Maternal Fetal Medicine Fellowship
Neonatology Fellowship
Child Abuse Pediatrics Fellowship
Bioethicist
Advanced Practice Provider
Nursing
Lawyer
Health Policy
Healthcare Administration
Other

## Slide 8
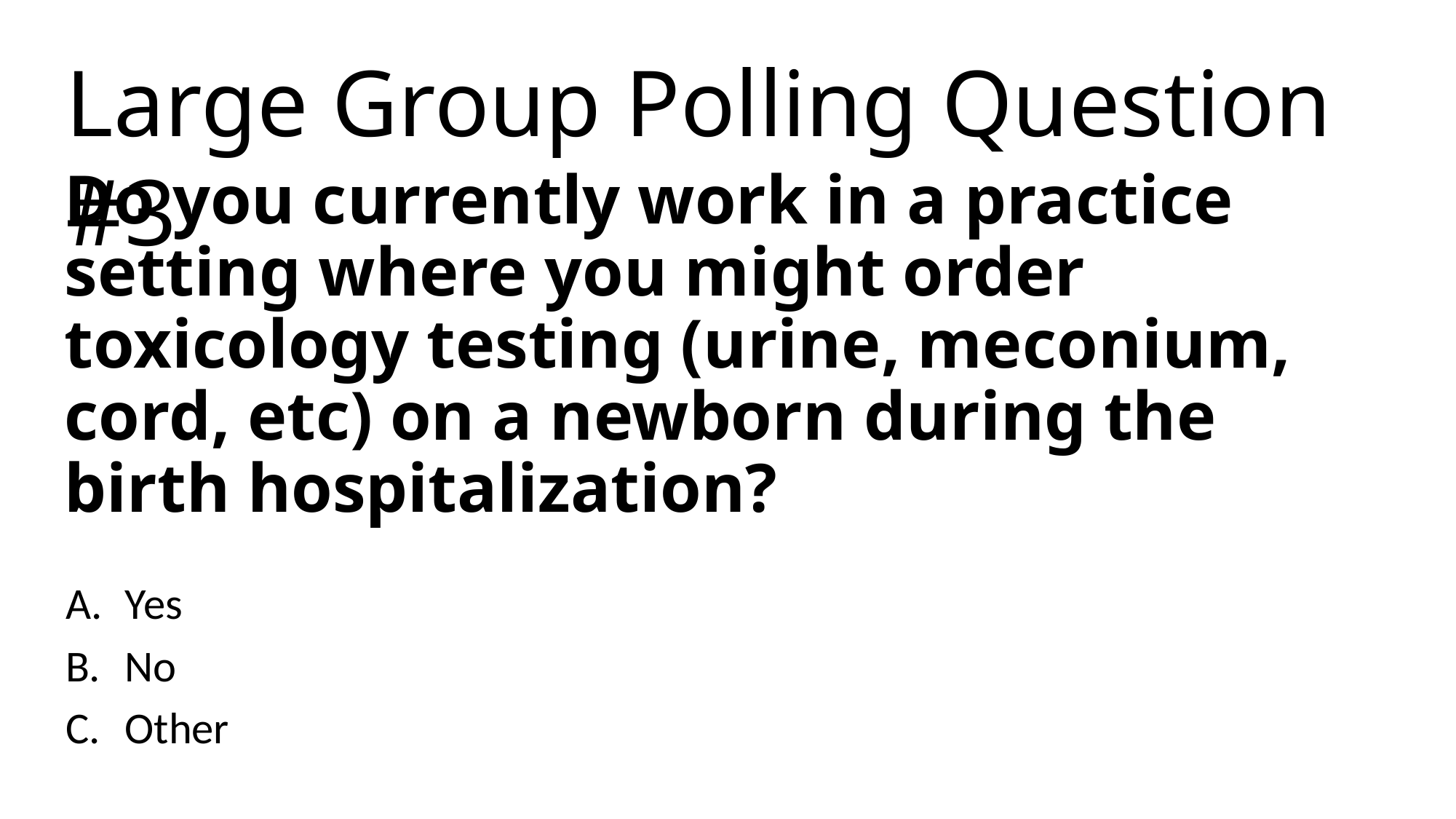

Large Group Polling Question #3
# Do you currently work in a practice setting where you might order toxicology testing (urine, meconium, cord, etc) on a newborn during the birth hospitalization?
Yes
No
Other

## Slide 9
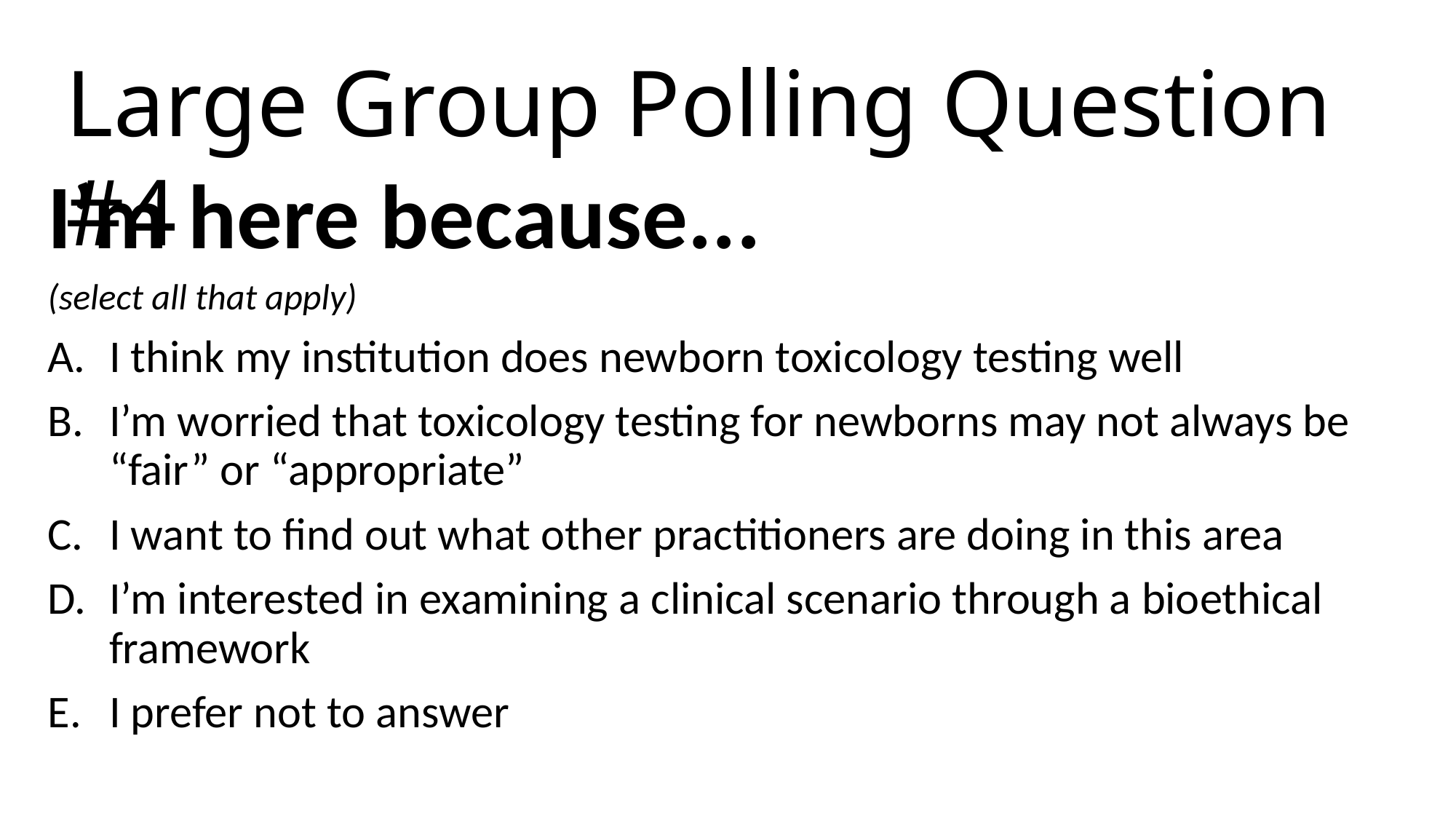

Large Group Polling Question #4
# I’m here because...
(select all that apply)
I think my institution does newborn toxicology testing well
I’m worried that toxicology testing for newborns may not always be “fair” or “appropriate”
I want to find out what other practitioners are doing in this area
I’m interested in examining a clinical scenario through a bioethical framework
I prefer not to answer

## Slide 10
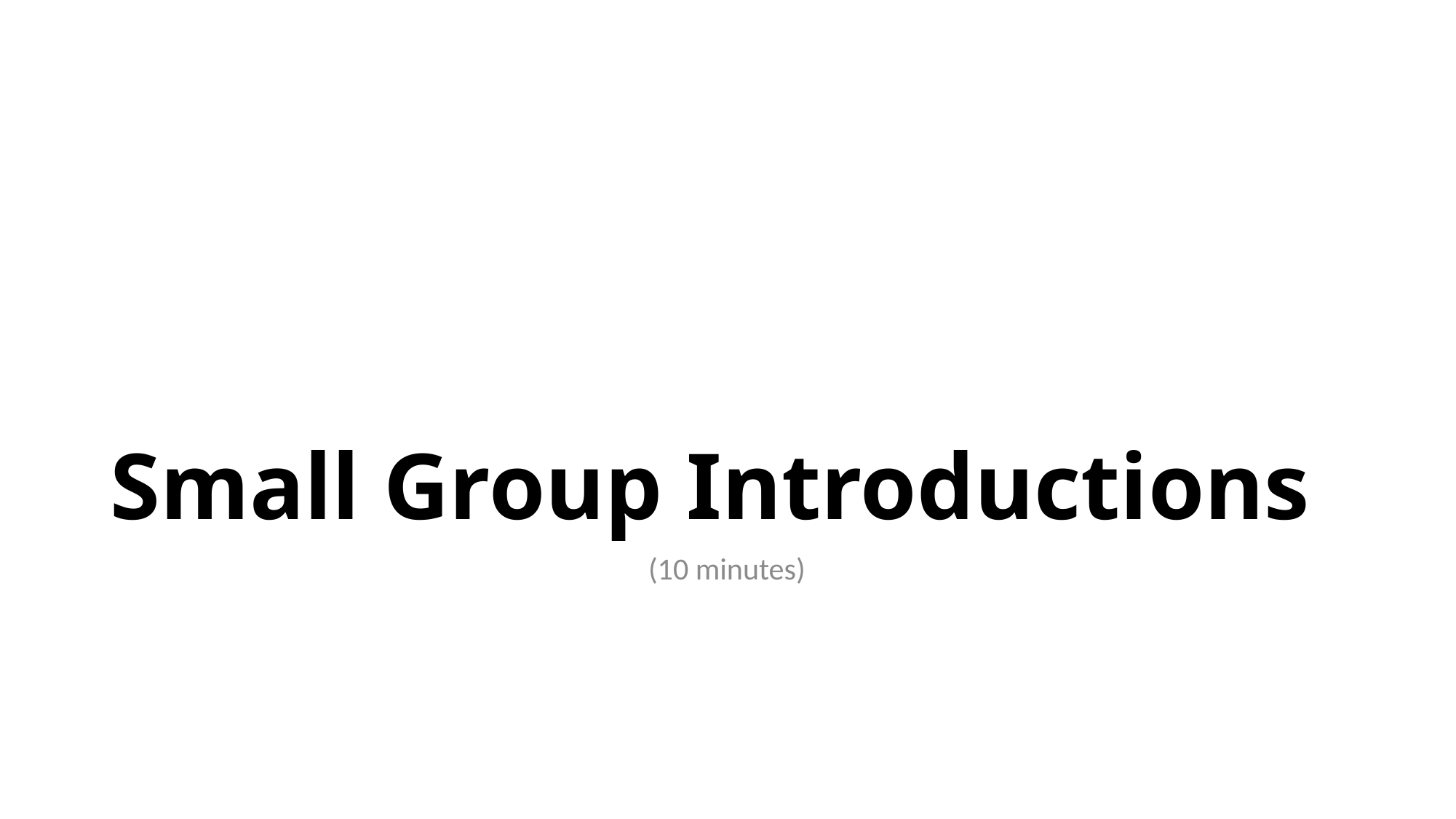

# Small Group Introductions
(10 minutes)

## Slide 11
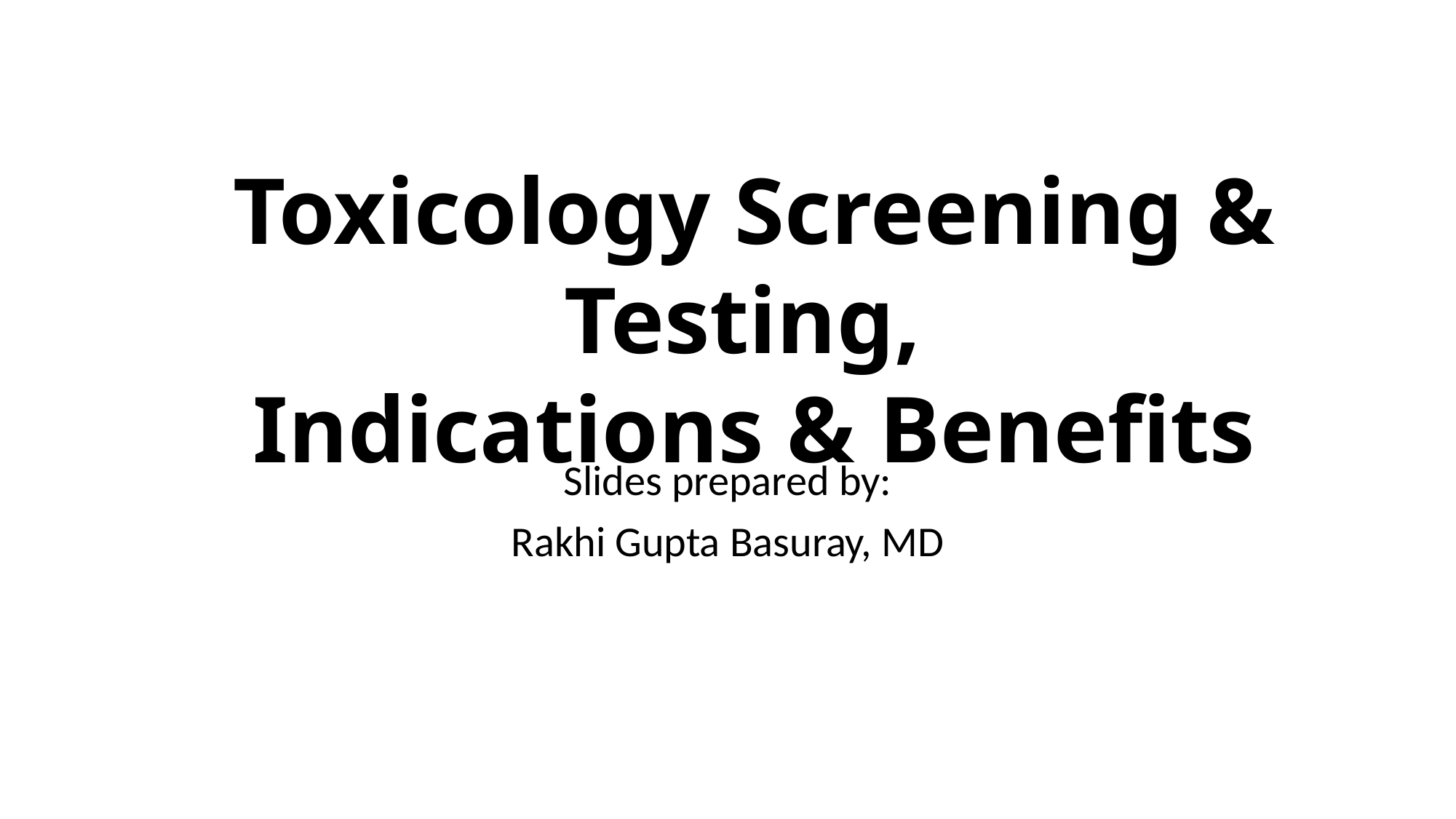

# Toxicology Screening & Testing, Indications & Benefits
Slides prepared by:
Rakhi Gupta Basuray, MD

## Slide 12
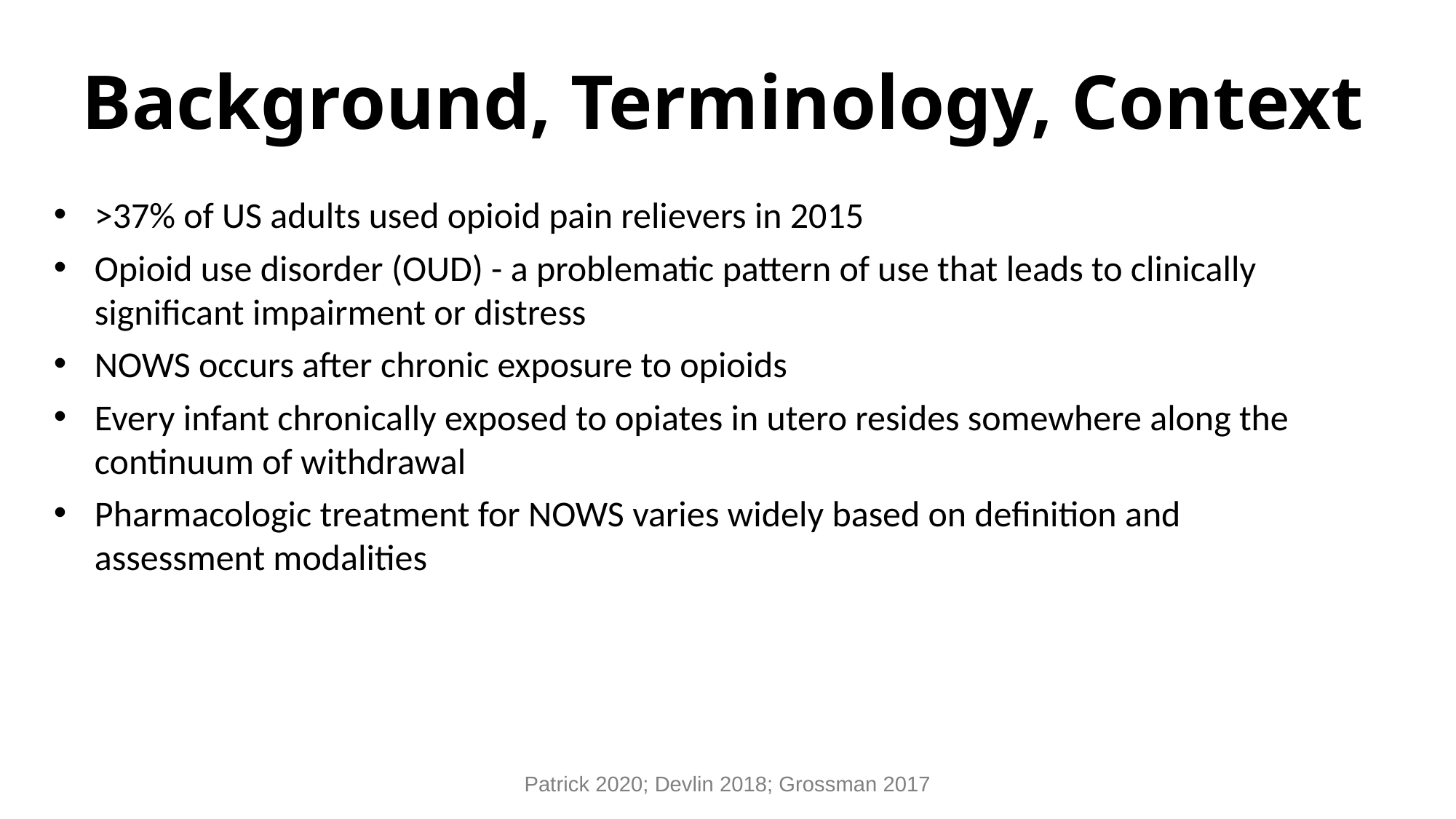

Background, Terminology, Context
>37% of US adults used opioid pain relievers in 2015
Opioid use disorder (OUD) - a problematic pattern of use that leads to clinically significant impairment or distress
NOWS occurs after chronic exposure to opioids
Every infant chronically exposed to opiates in utero resides somewhere along the continuum of withdrawal
Pharmacologic treatment for NOWS varies widely based on definition and assessment modalities
Patrick 2020; Devlin 2018; Grossman 2017

## Slide 13
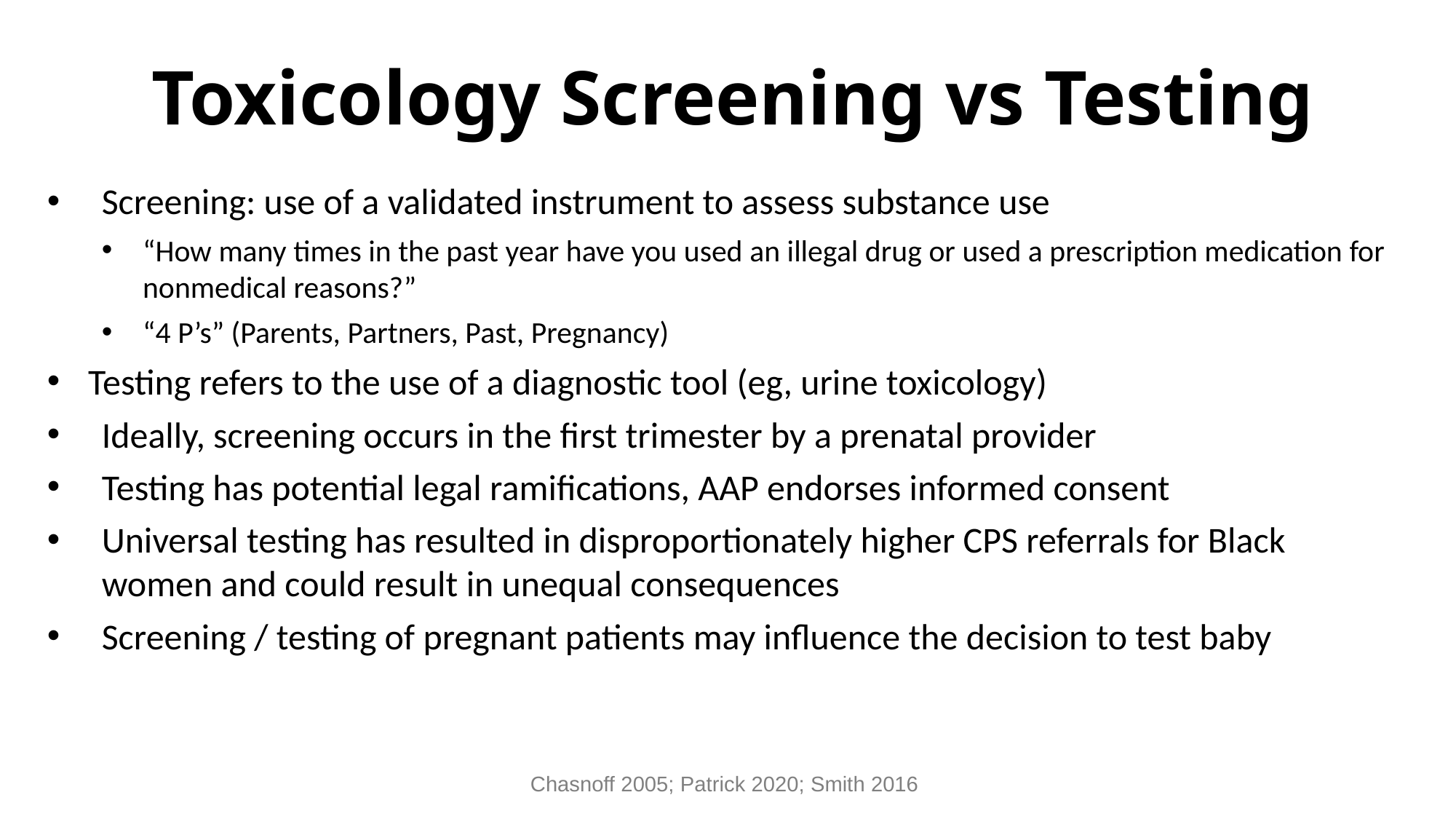

Toxicology Screening vs Testing
Screening: use of a validated instrument to assess substance use
“How many times in the past year have you used an illegal drug or used a prescription medication for nonmedical reasons?”
“4 P’s” (Parents, Partners, Past, Pregnancy)
Testing refers to the use of a diagnostic tool (eg, urine toxicology)
Ideally, screening occurs in the first trimester by a prenatal provider
Testing has potential legal ramifications, AAP endorses informed consent
Universal testing has resulted in disproportionately higher CPS referrals for Black women and could result in unequal consequences
Screening / testing of pregnant patients may influence the decision to test baby
Chasnoff 2005; Patrick 2020; Smith 2016

## Slide 14
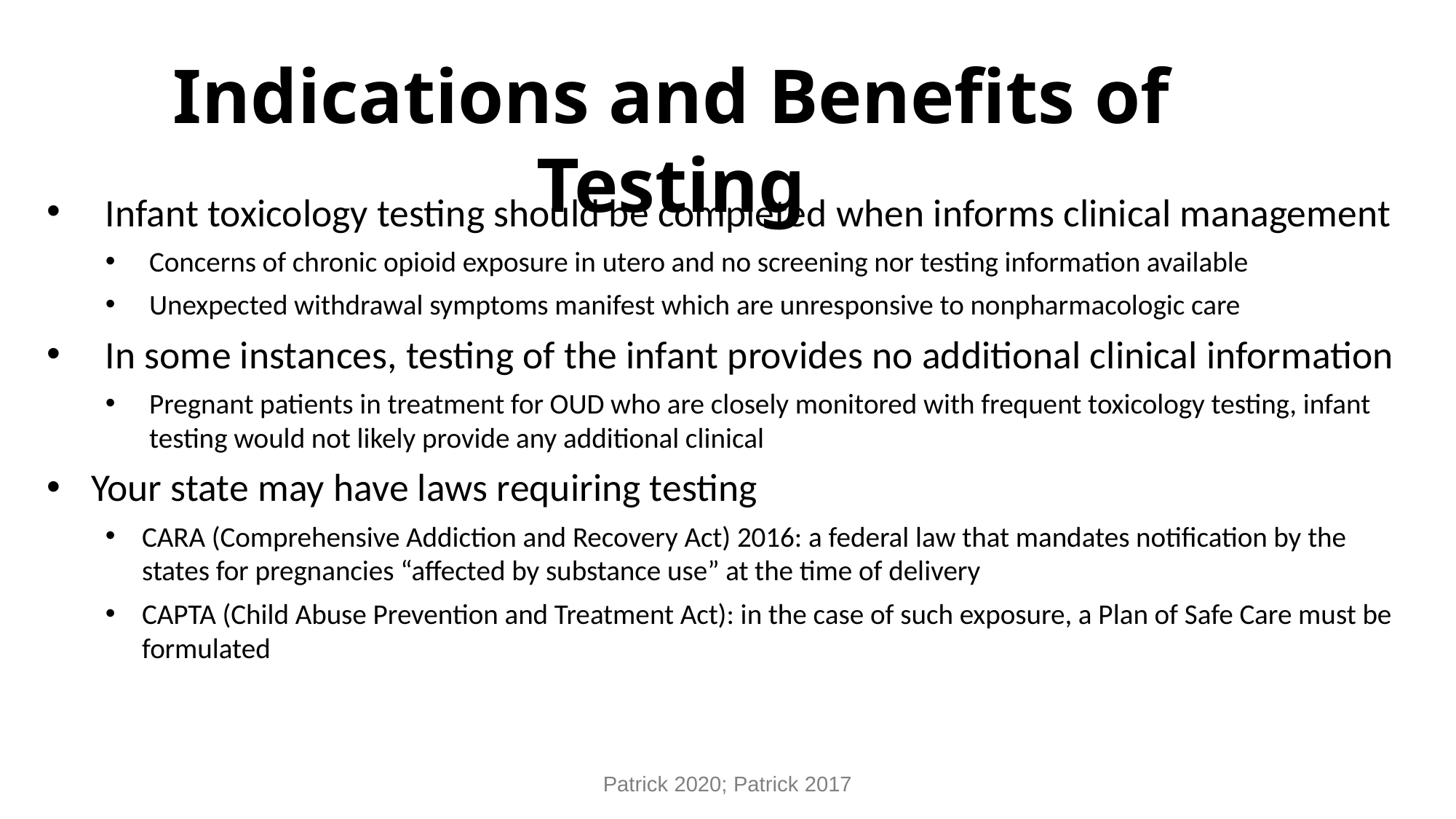

Indications and Benefits of Testing
Infant toxicology testing should be completed when informs clinical management
Concerns of chronic opioid exposure in utero and no screening nor testing information available
Unexpected withdrawal symptoms manifest which are unresponsive to nonpharmacologic care
In some instances, testing of the infant provides no additional clinical information
Pregnant patients in treatment for OUD who are closely monitored with frequent toxicology testing, infant testing would not likely provide any additional clinical
Your state may have laws requiring testing
CARA (Comprehensive Addiction and Recovery Act) 2016: a federal law that mandates notification by the states for pregnancies “affected by substance use” at the time of delivery
CAPTA (Child Abuse Prevention and Treatment Act): in the case of such exposure, a Plan of Safe Care must be formulated
Patrick 2020; Patrick 2017

## Slide 15
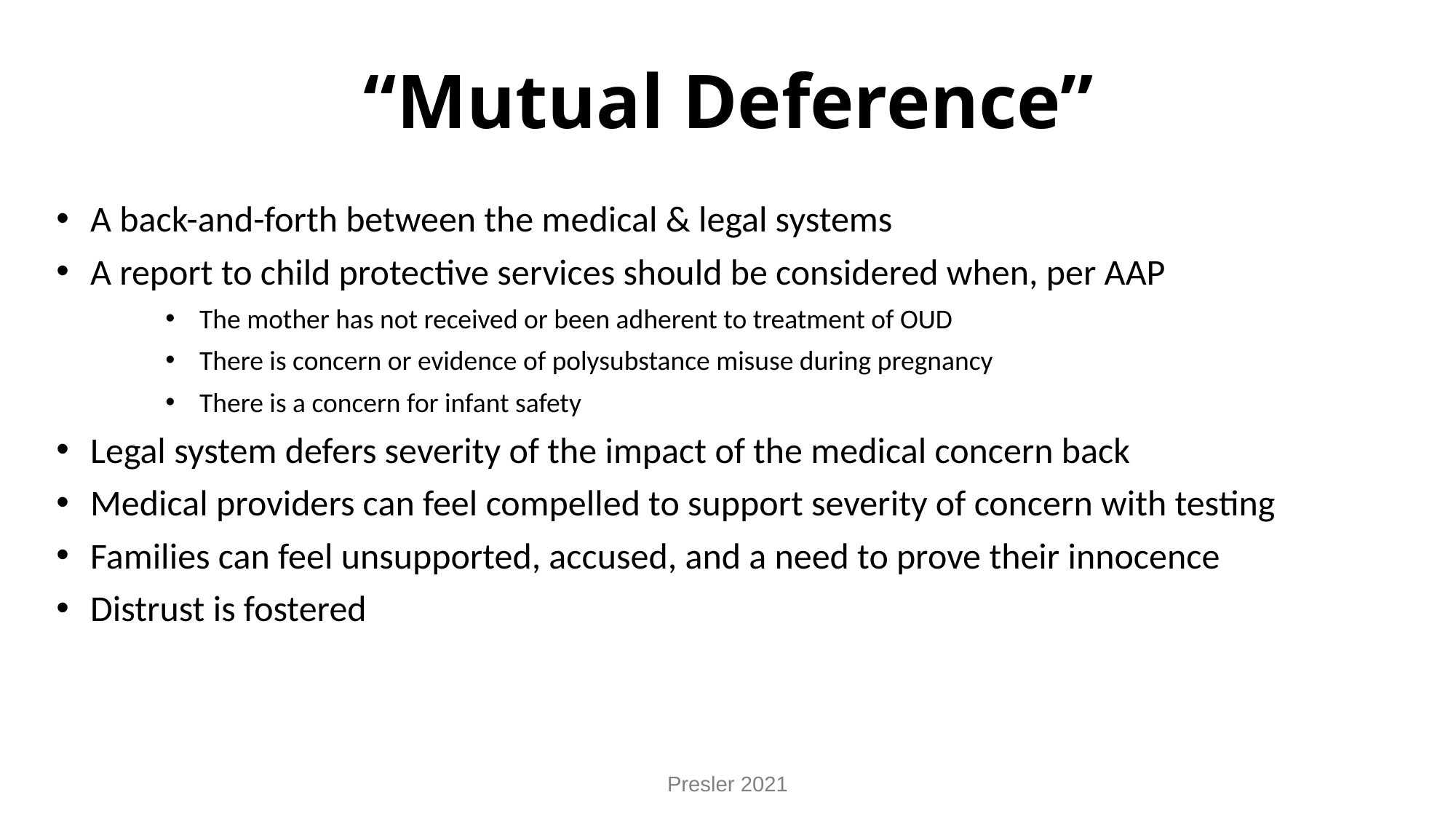

“Mutual Deference”
A back-and-forth between the medical & legal systems
A report to child protective services should be considered when, per AAP
The mother has not received or been adherent to treatment of OUD
There is concern or evidence of polysubstance misuse during pregnancy
There is a concern for infant safety
Legal system defers severity of the impact of the medical concern back
Medical providers can feel compelled to support severity of concern with testing
Families can feel unsupported, accused, and a need to prove their innocence
Distrust is fostered
Presler 2021

## Slide 16
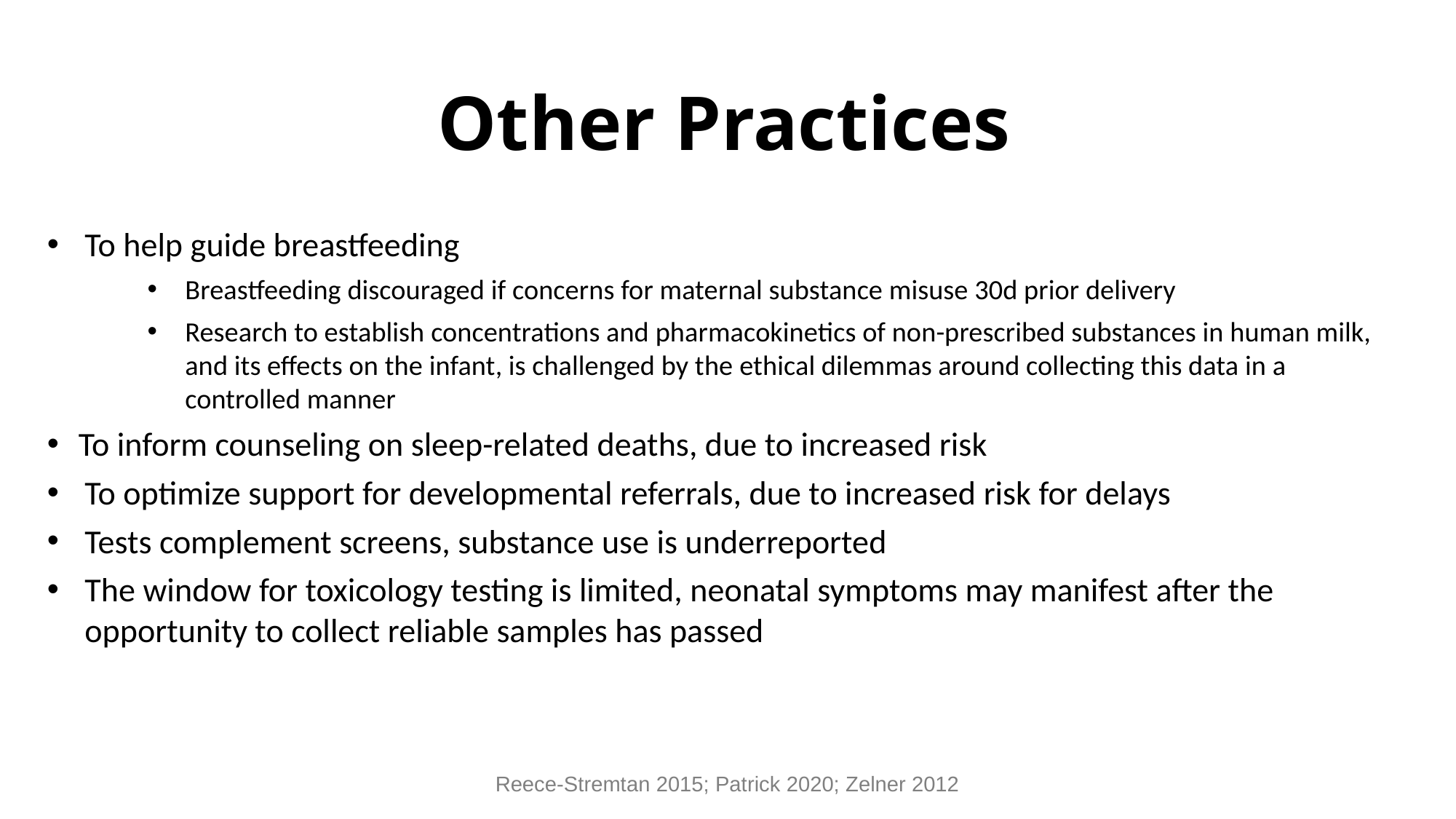

Other Practices
To help guide breastfeeding
Breastfeeding discouraged if concerns for maternal substance misuse 30d prior delivery
Research to establish concentrations and pharmacokinetics of non-prescribed substances in human milk, and its effects on the infant, is challenged by the ethical dilemmas around collecting this data in a controlled manner
To inform counseling on sleep-related deaths, due to increased risk
To optimize support for developmental referrals, due to increased risk for delays
Tests complement screens, substance use is underreported
The window for toxicology testing is limited, neonatal symptoms may manifest after the opportunity to collect reliable samples has passed
Reece-Stremtan 2015; Patrick 2020; Zelner 2012

## Slide 17
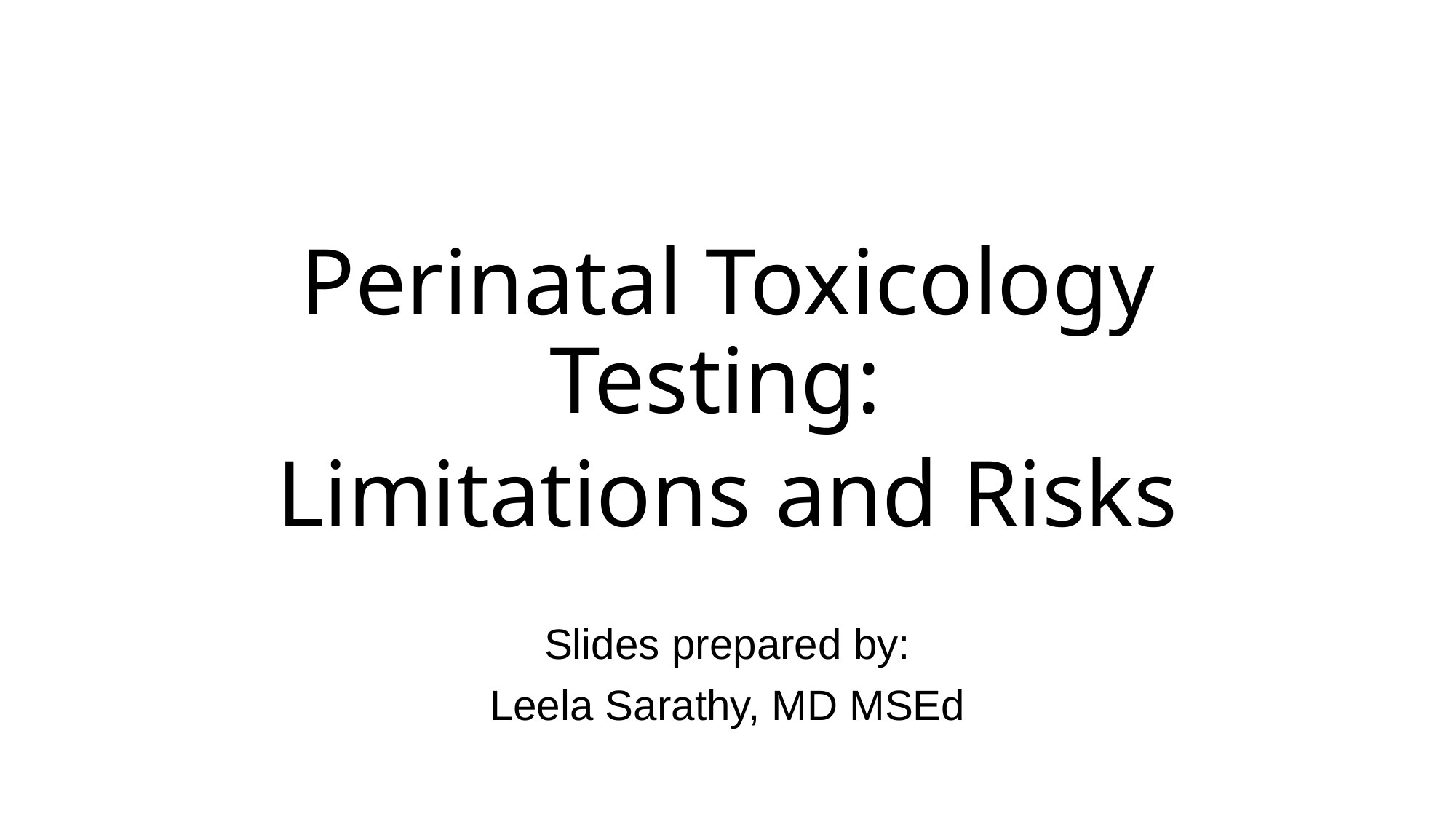

Perinatal Toxicology Testing:
Limitations and Risks
Slides prepared by:
Leela Sarathy, MD MSEd

## Slide 18
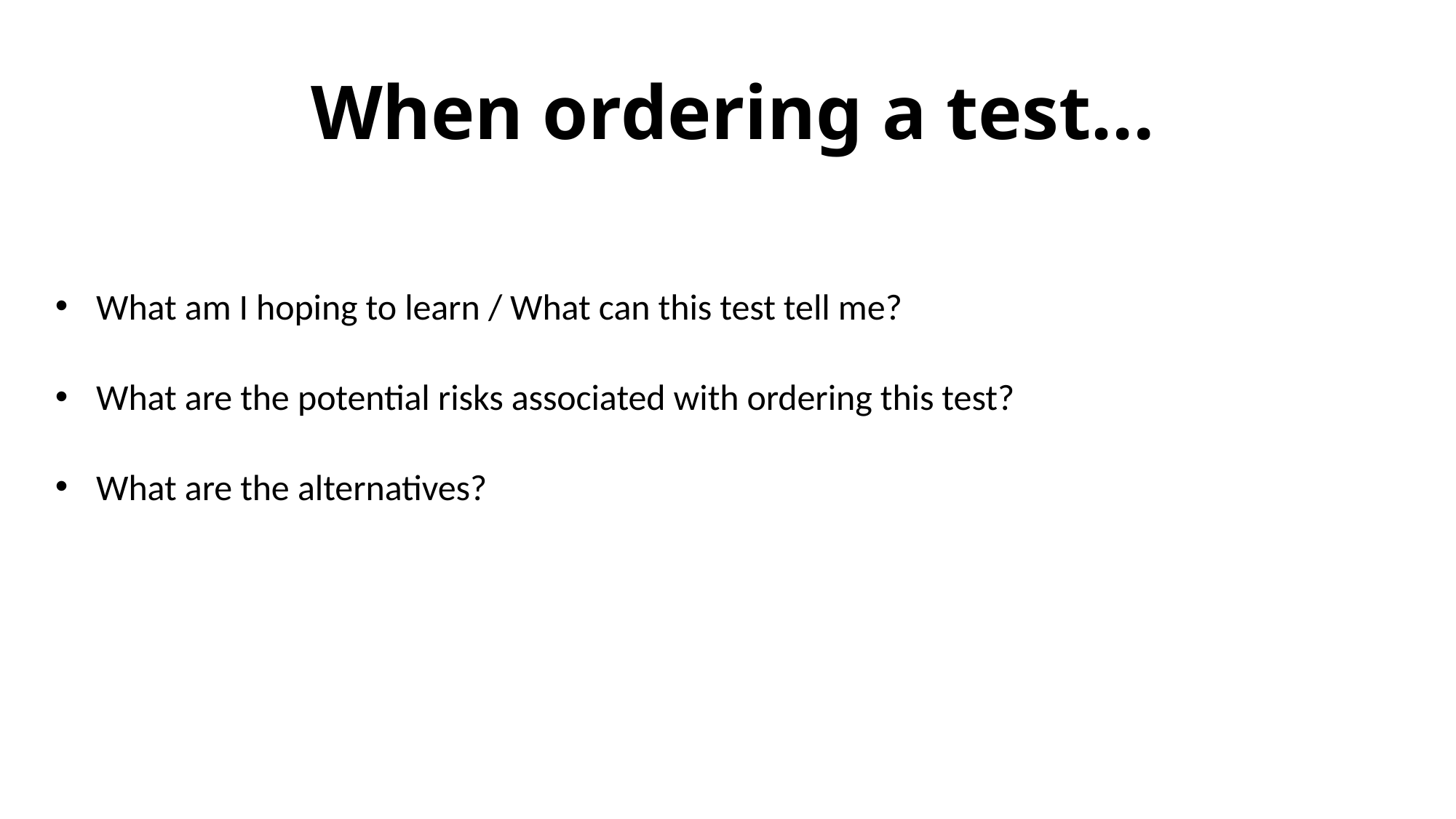

When ordering a test…
What am I hoping to learn / What can this test tell me?
What are the potential risks associated with ordering this test?
What are the alternatives?

## Slide 19
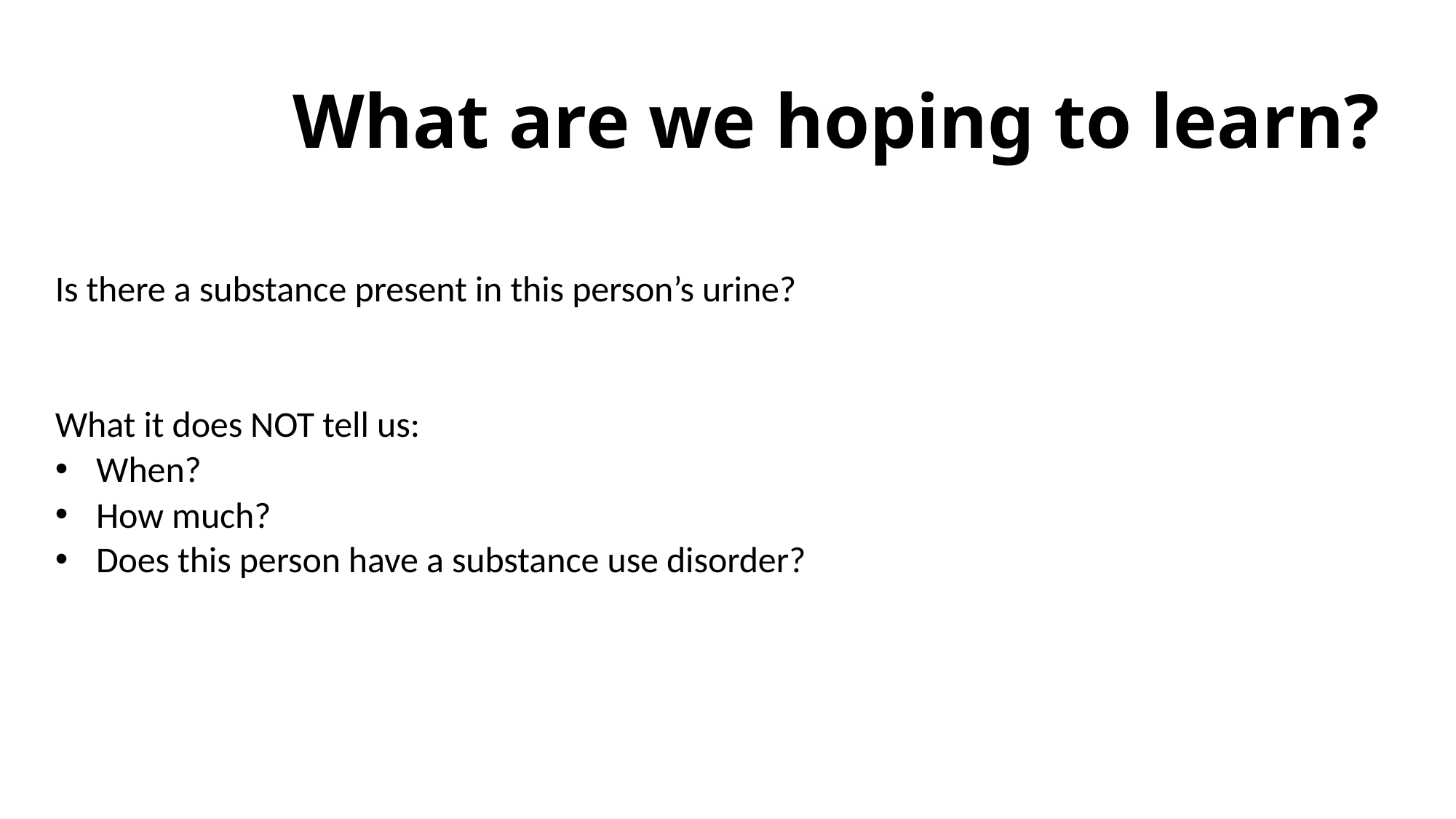

What are we hoping to learn?
Is there a substance present in this person’s urine?
What it does NOT tell us:
When?
How much?
Does this person have a substance use disorder?

## Slide 20
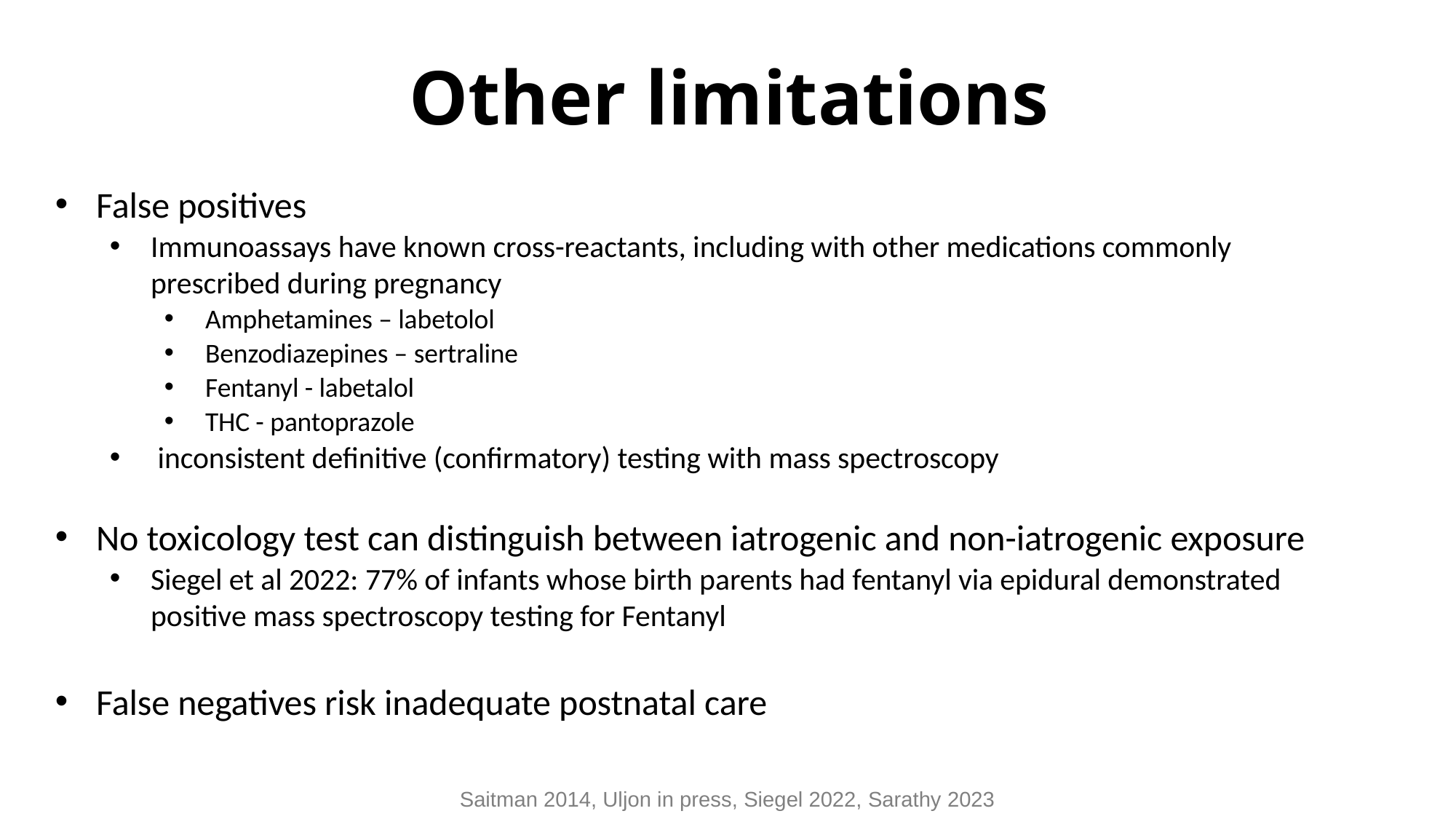

Other limitations
False positives
Immunoassays have known cross-reactants, including with other medications commonly prescribed during pregnancy
Amphetamines – labetolol
Benzodiazepines – sertraline
Fentanyl - labetalol
THC - pantoprazole
 inconsistent definitive (confirmatory) testing with mass spectroscopy
No toxicology test can distinguish between iatrogenic and non-iatrogenic exposure
Siegel et al 2022: 77% of infants whose birth parents had fentanyl via epidural demonstrated positive mass spectroscopy testing for Fentanyl
False negatives risk inadequate postnatal care
Saitman 2014, Uljon in press, Siegel 2022, Sarathy 2023

## Slide 21
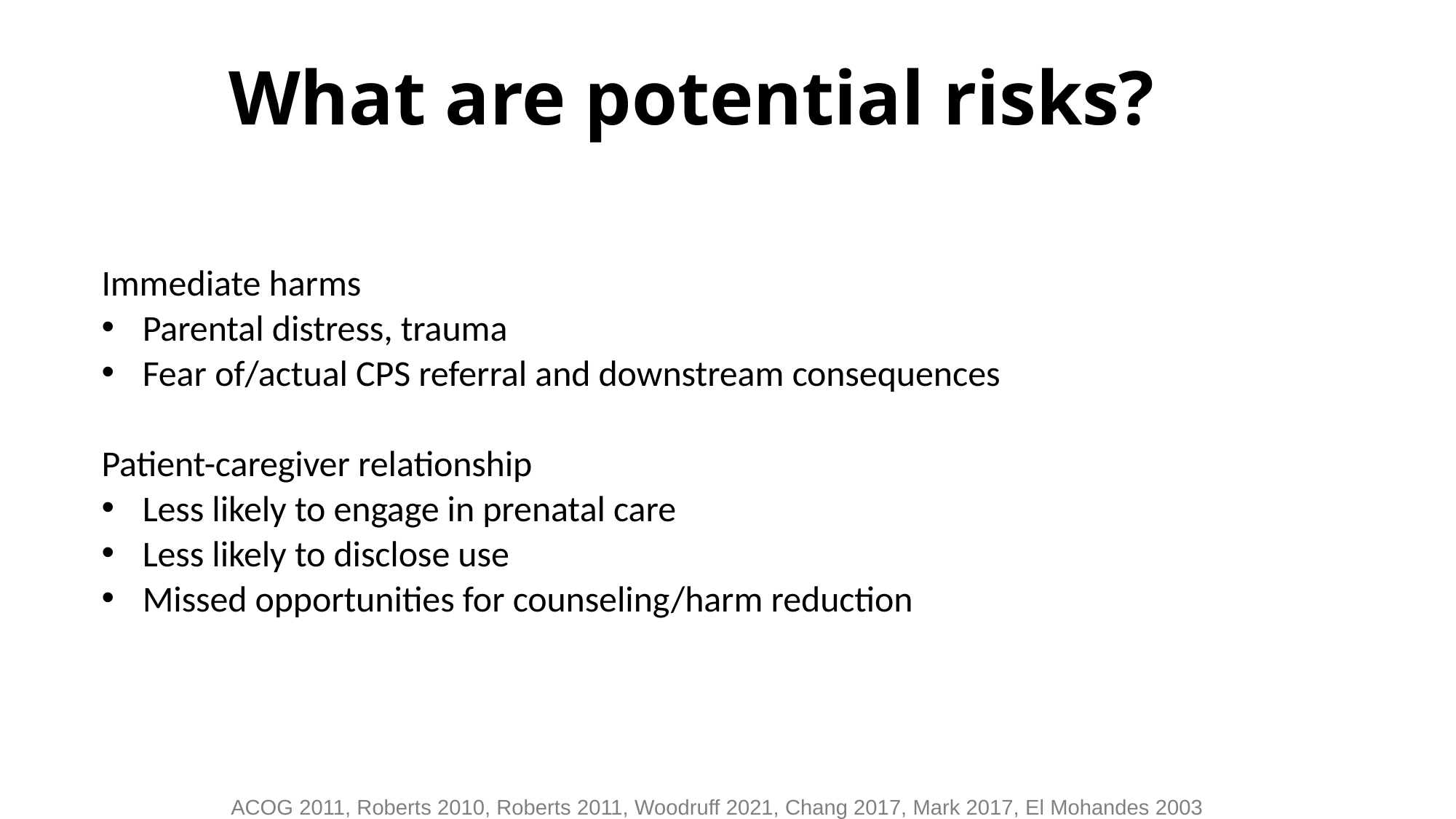

What are potential risks?
Immediate harms
Parental distress, trauma
Fear of/actual CPS referral and downstream consequences
Patient-caregiver relationship
Less likely to engage in prenatal care
Less likely to disclose use
Missed opportunities for counseling/harm reduction
ACOG 2011, Roberts 2010, Roberts 2011, Woodruff 2021, Chang 2017, Mark 2017, El Mohandes 2003

## Slide 22
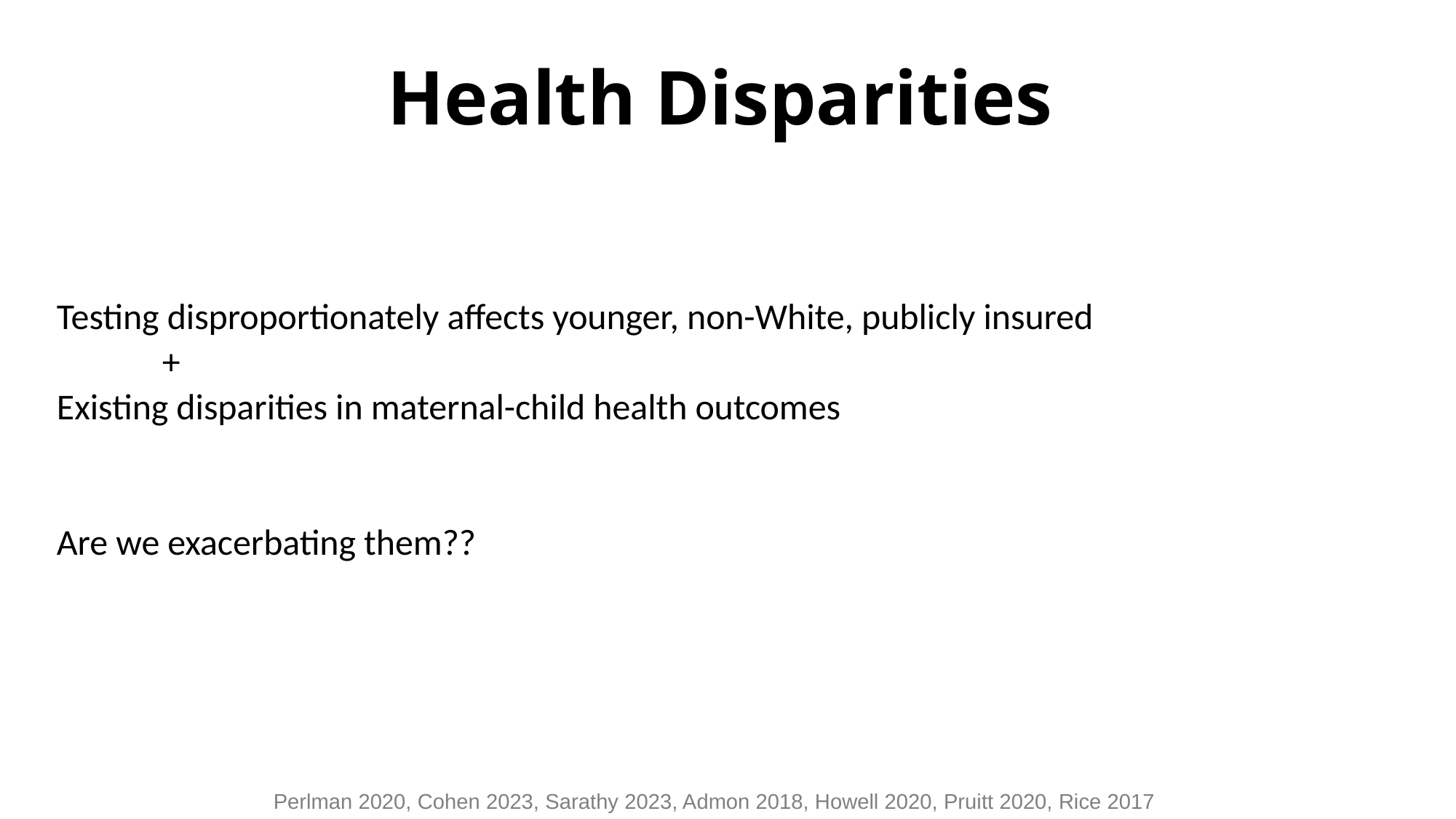

Health Disparities
Testing disproportionately affects younger, non-White, publicly insured
 +
Existing disparities in maternal-child health outcomes
Are we exacerbating them??
Perlman 2020, Cohen 2023, Sarathy 2023, Admon 2018, Howell 2020, Pruitt 2020, Rice 2017

## Slide 23
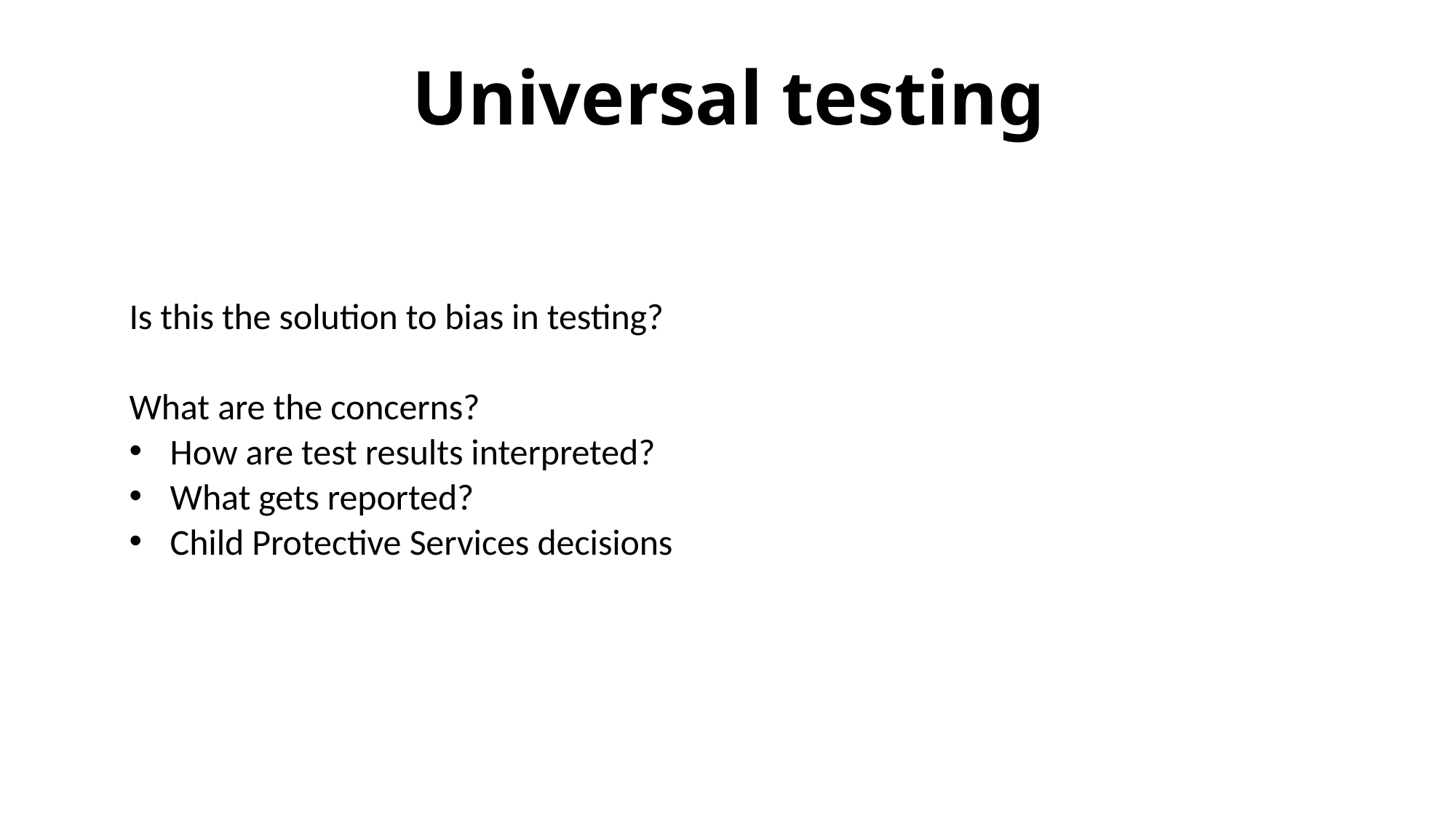

Universal testing
Is this the solution to bias in testing?
What are the concerns?
How are test results interpreted?
What gets reported?
Child Protective Services decisions

## Slide 24
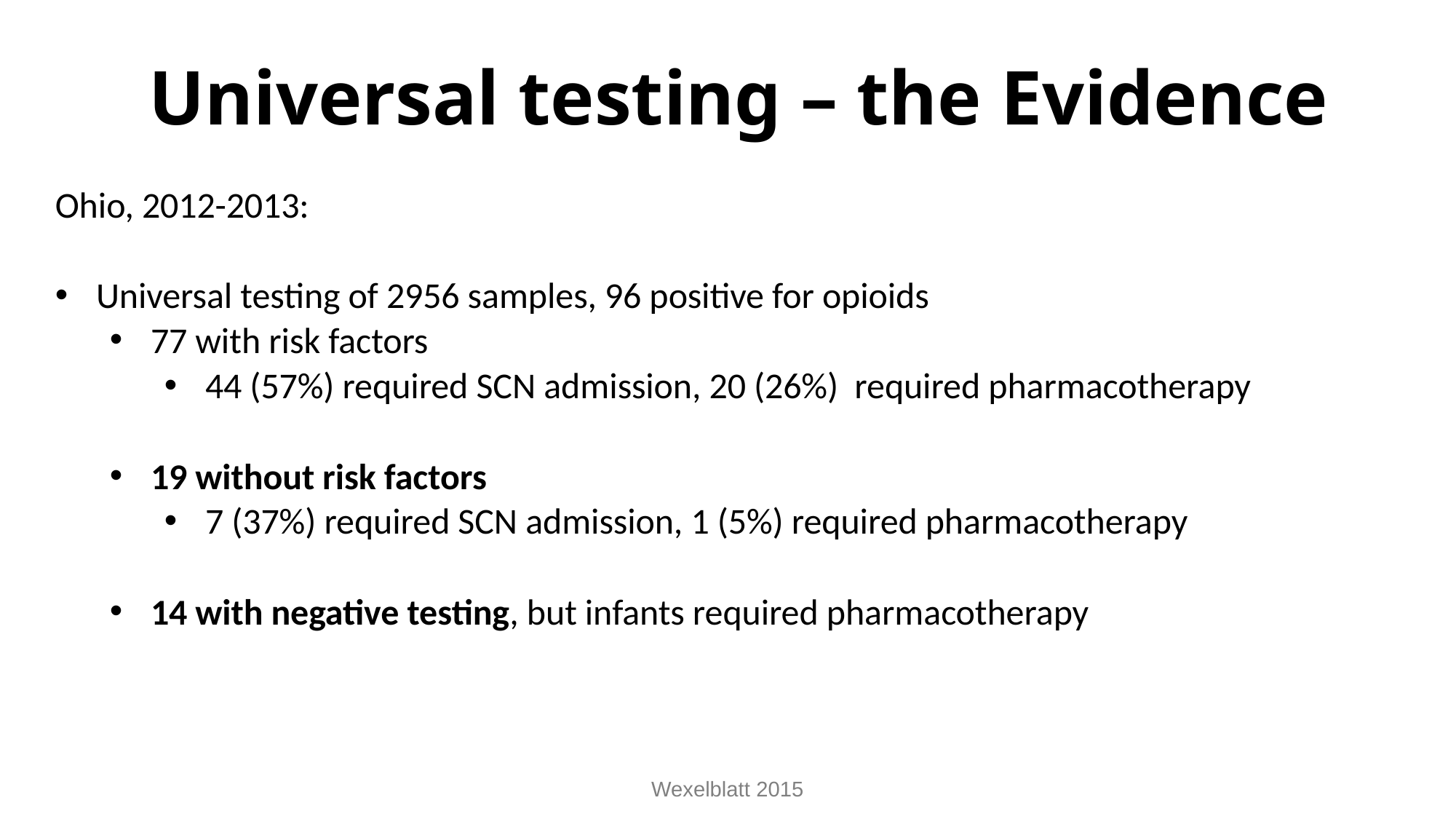

Universal testing – the Evidence
Ohio, 2012-2013:
Universal testing of 2956 samples, 96 positive for opioids
77 with risk factors
44 (57%) required SCN admission, 20 (26%) required pharmacotherapy
19 without risk factors
7 (37%) required SCN admission, 1 (5%) required pharmacotherapy
14 with negative testing, but infants required pharmacotherapy
Wexelblatt 2015

## Slide 25
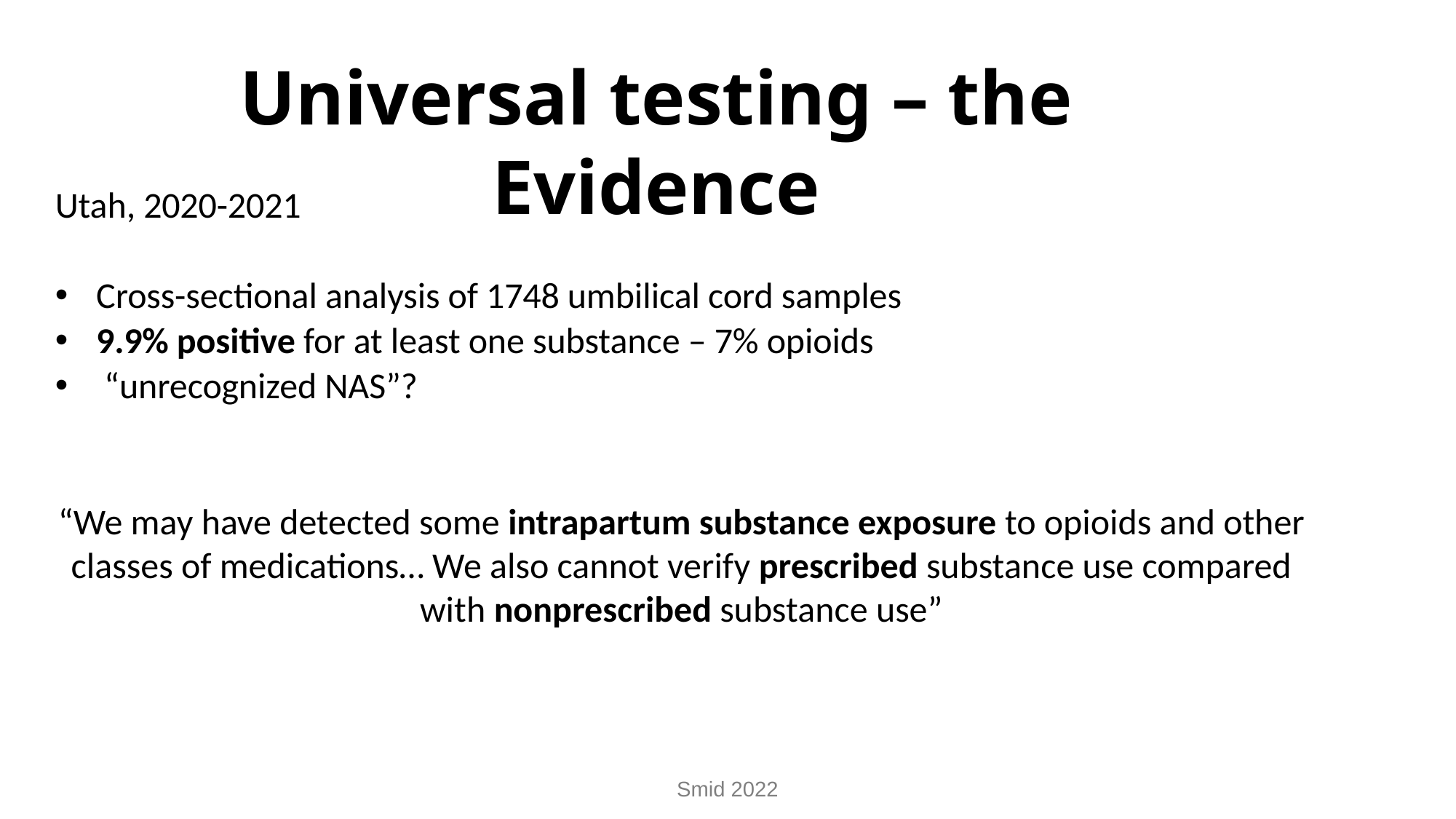

Universal testing – the Evidence
Utah, 2020-2021
Cross-sectional analysis of 1748 umbilical cord samples
9.9% positive for at least one substance – 7% opioids
 “unrecognized NAS”?
“We may have detected some intrapartum substance exposure to opioids and other classes of medications… We also cannot verify prescribed substance use compared with nonprescribed substance use”
Smid 2022

## Slide 26
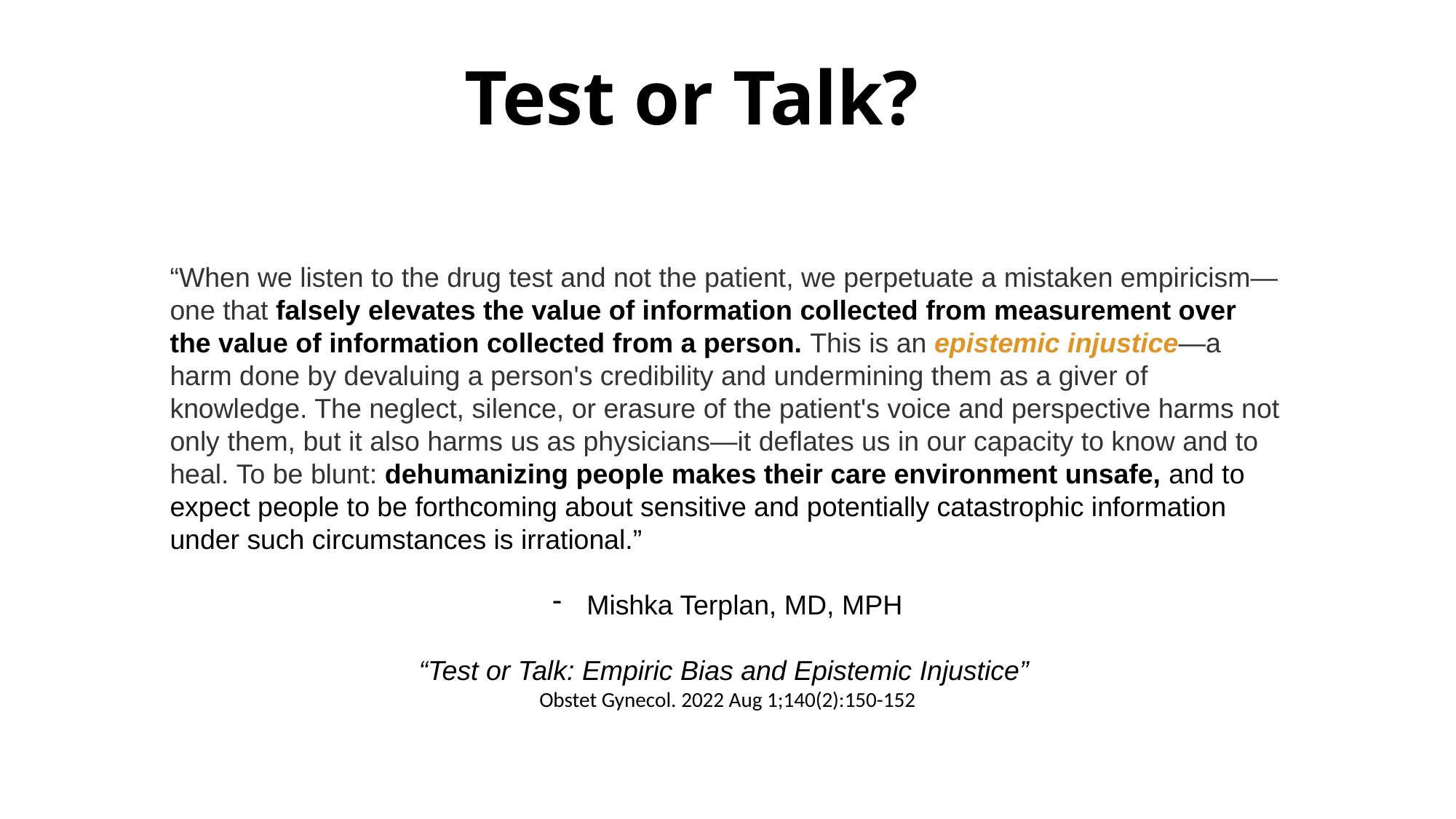

Test or Talk?
“When we listen to the drug test and not the patient, we perpetuate a mistaken empiricism—one that falsely elevates the value of information collected from measurement over the value of information collected from a person. This is an epistemic injustice—a harm done by devaluing a person's credibility and undermining them as a giver of knowledge. The neglect, silence, or erasure of the patient's voice and perspective harms not only them, but it also harms us as physicians—it deflates us in our capacity to know and to heal. To be blunt: dehumanizing people makes their care environment unsafe, and to expect people to be forthcoming about sensitive and potentially catastrophic information under such circumstances is irrational.”
Mishka Terplan, MD, MPH
“Test or Talk: Empiric Bias and Epistemic Injustice”
Obstet Gynecol. 2022 Aug 1;140(2):150-152

## Slide 27
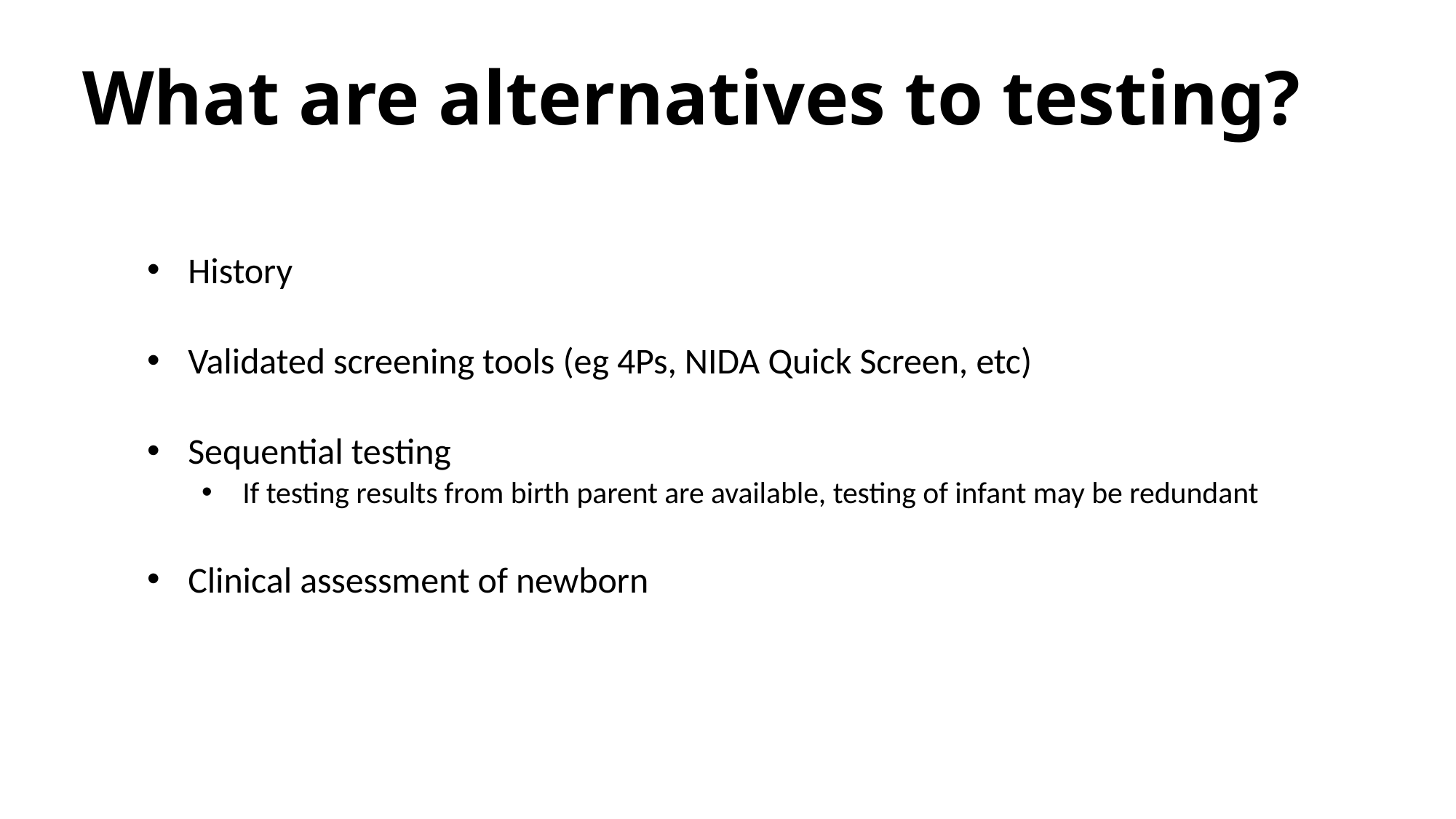

What are alternatives to testing?
History
Validated screening tools (eg 4Ps, NIDA Quick Screen, etc)
Sequential testing
If testing results from birth parent are available, testing of infant may be redundant
Clinical assessment of newborn

## Slide 28
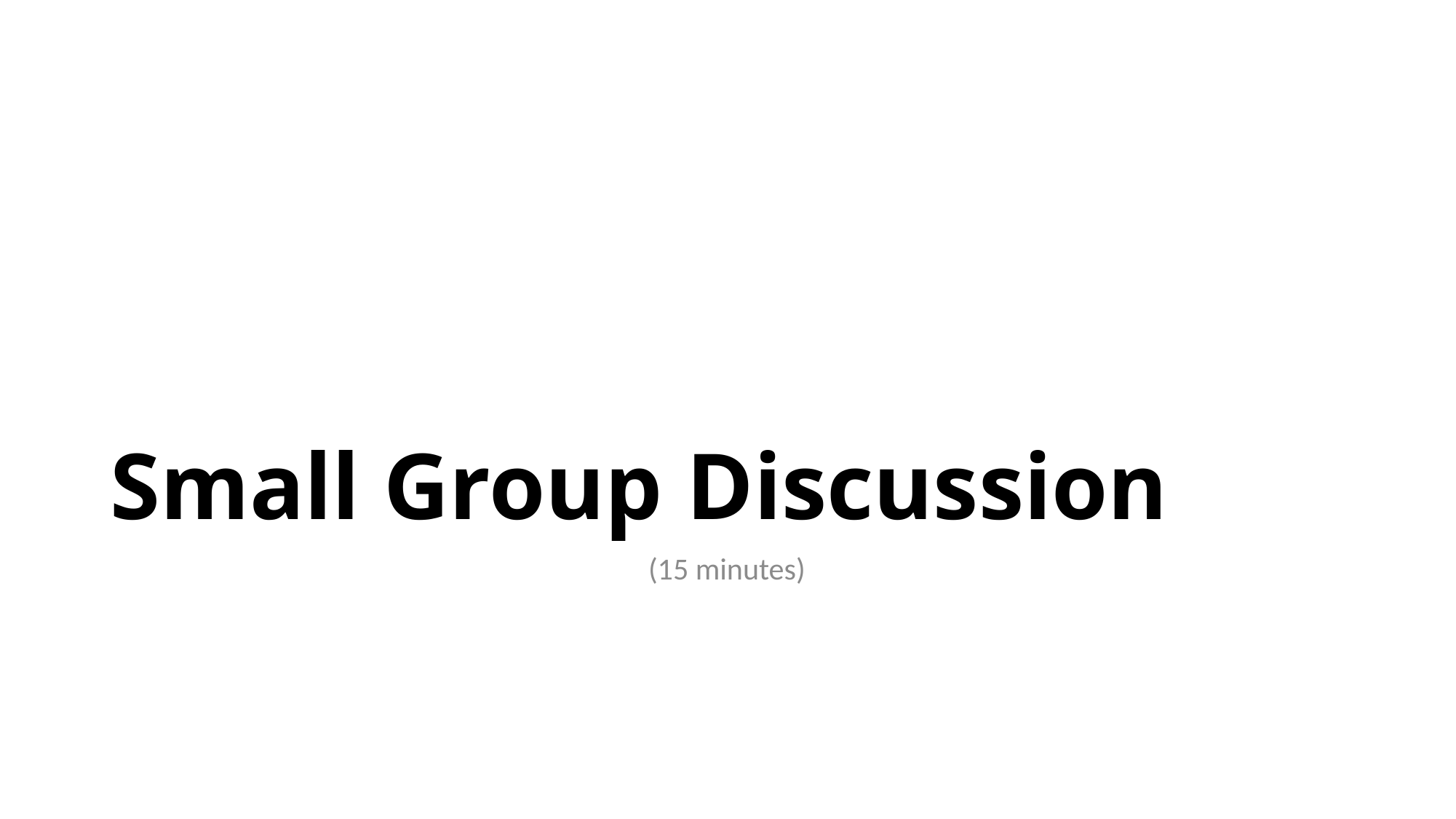

# Small Group Discussion
(15 minutes)

## Slide 29
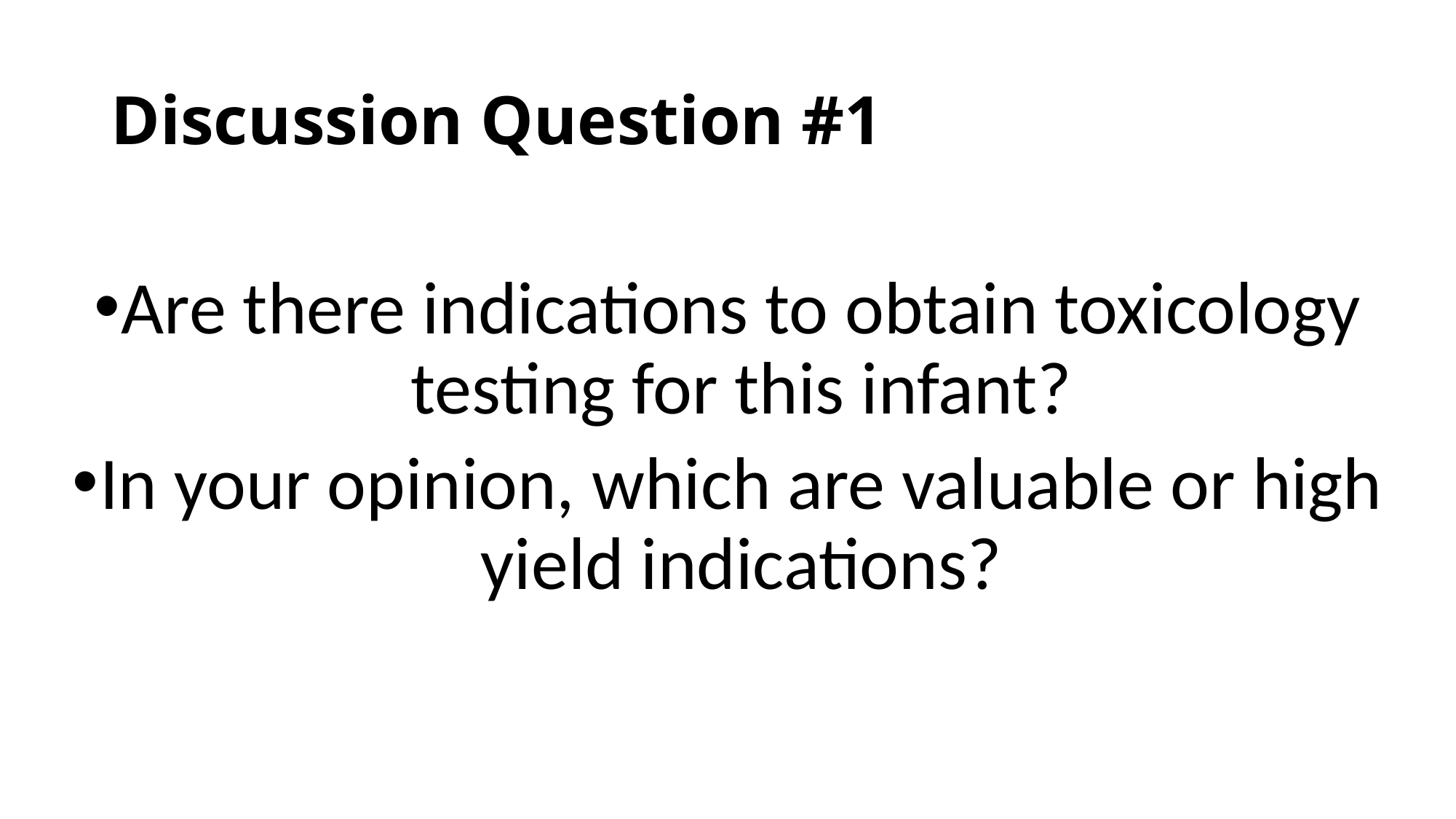

# Discussion Question #1
Are there indications to obtain toxicology testing for this infant?
In your opinion, which are valuable or high yield indications?

## Slide 30
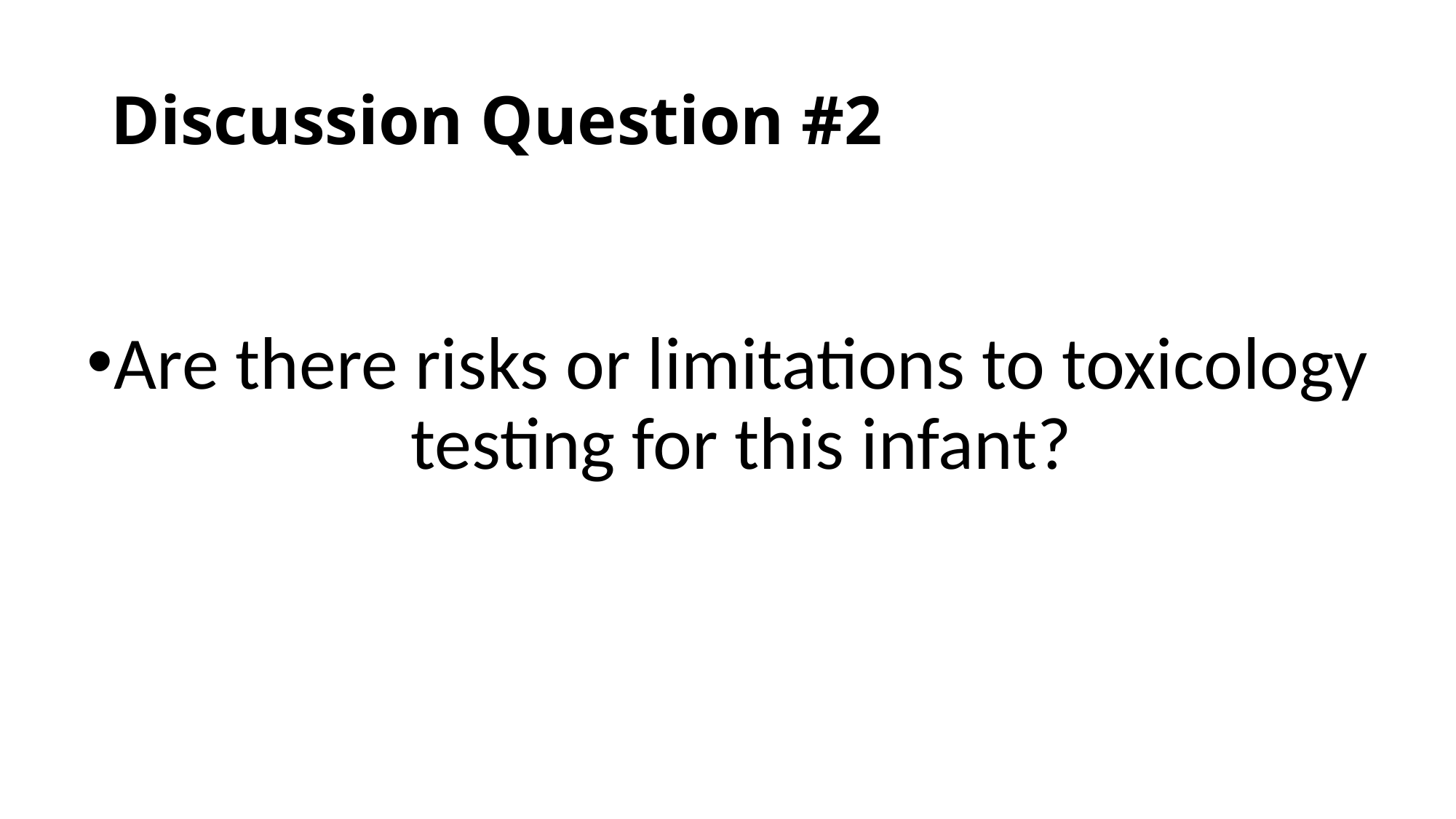

# Discussion Question #2
Are there risks or limitations to toxicology testing for this infant?

## Slide 31
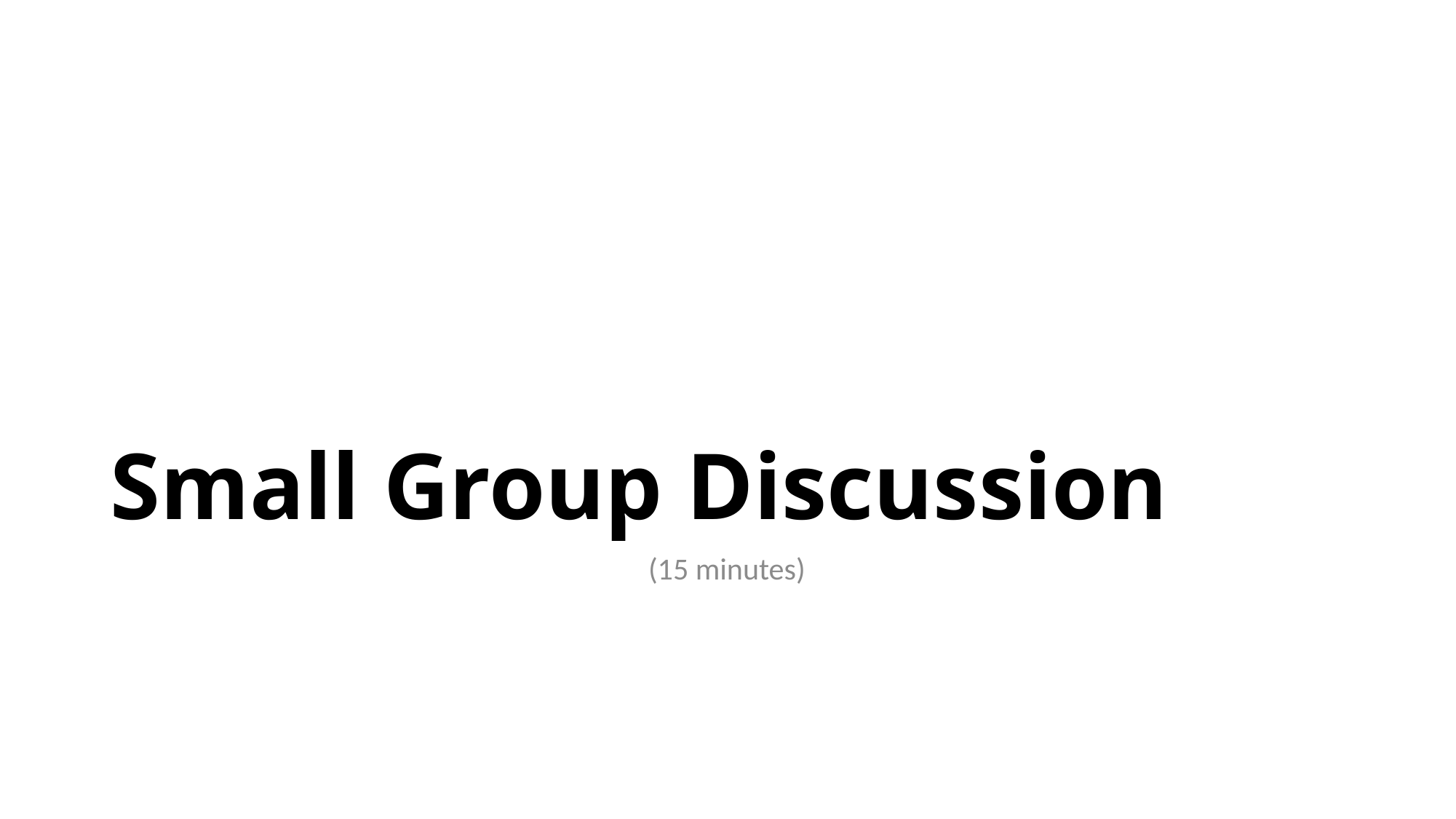

# Small Group Discussion
(15 minutes)

## Slide 32
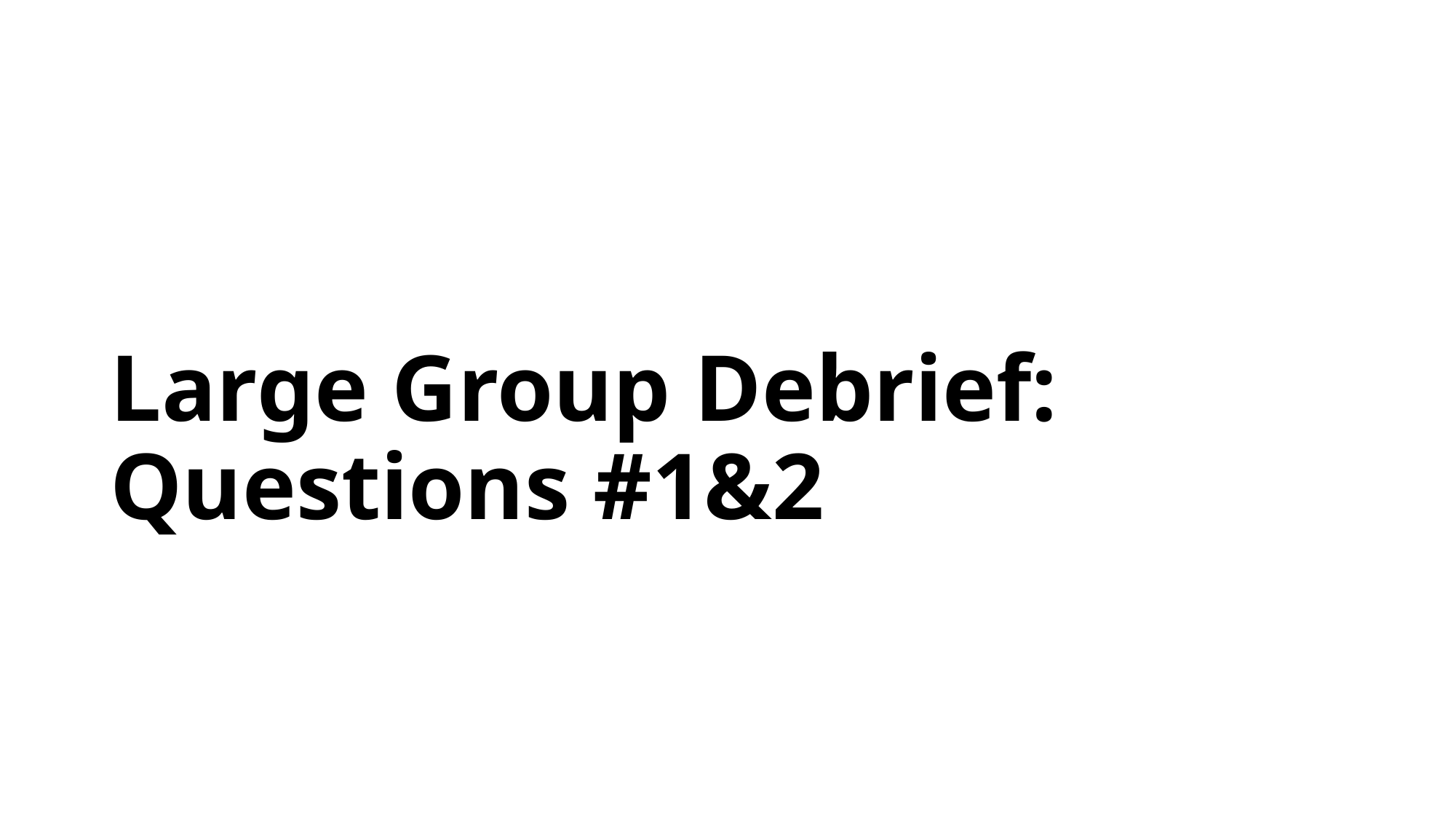

# Large Group Debrief: Questions #1&2

## Slide 33
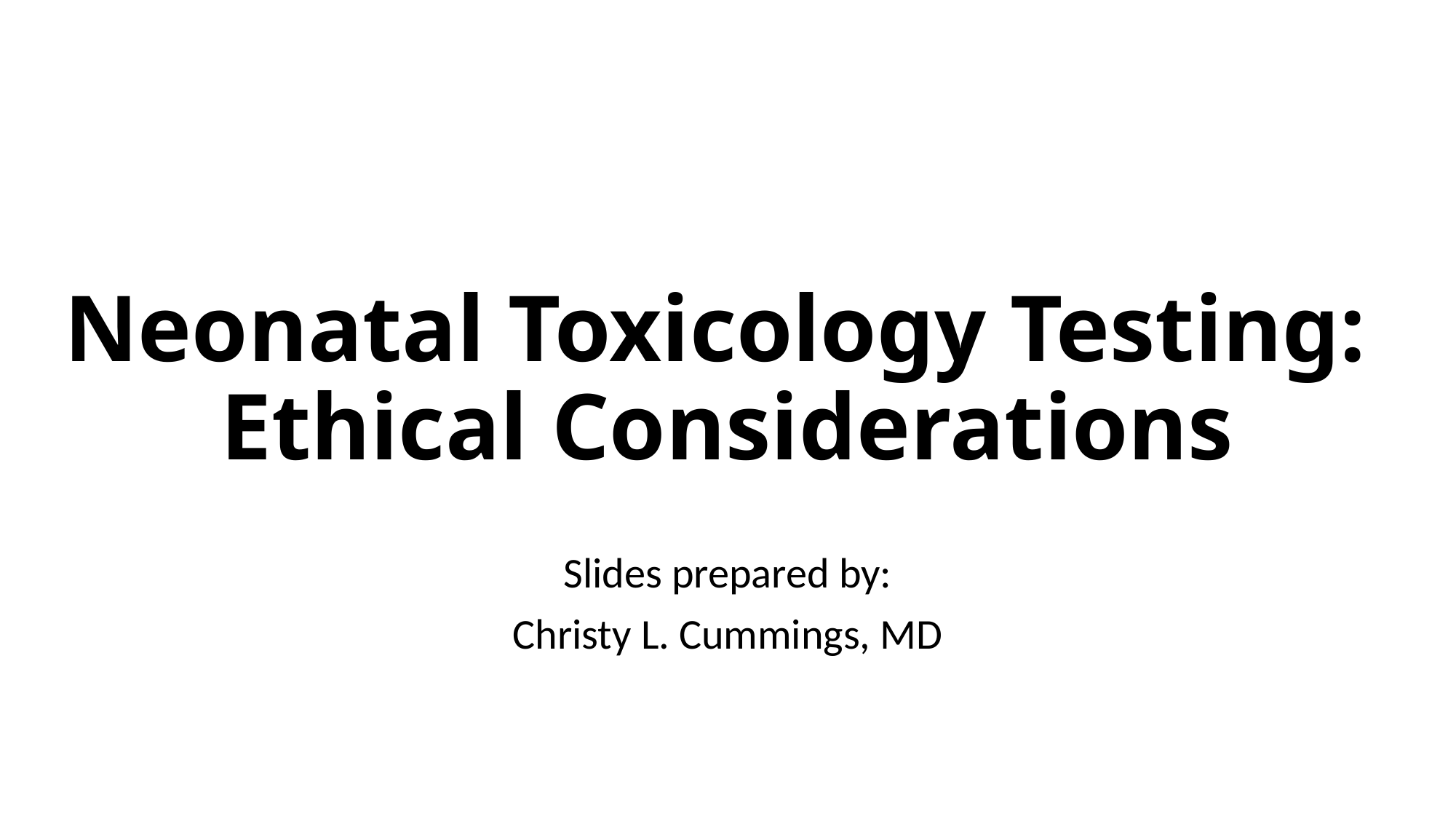

# Neonatal Toxicology Testing: Ethical Considerations
Slides prepared by:
Christy L. Cummings, MD

## Slide 34
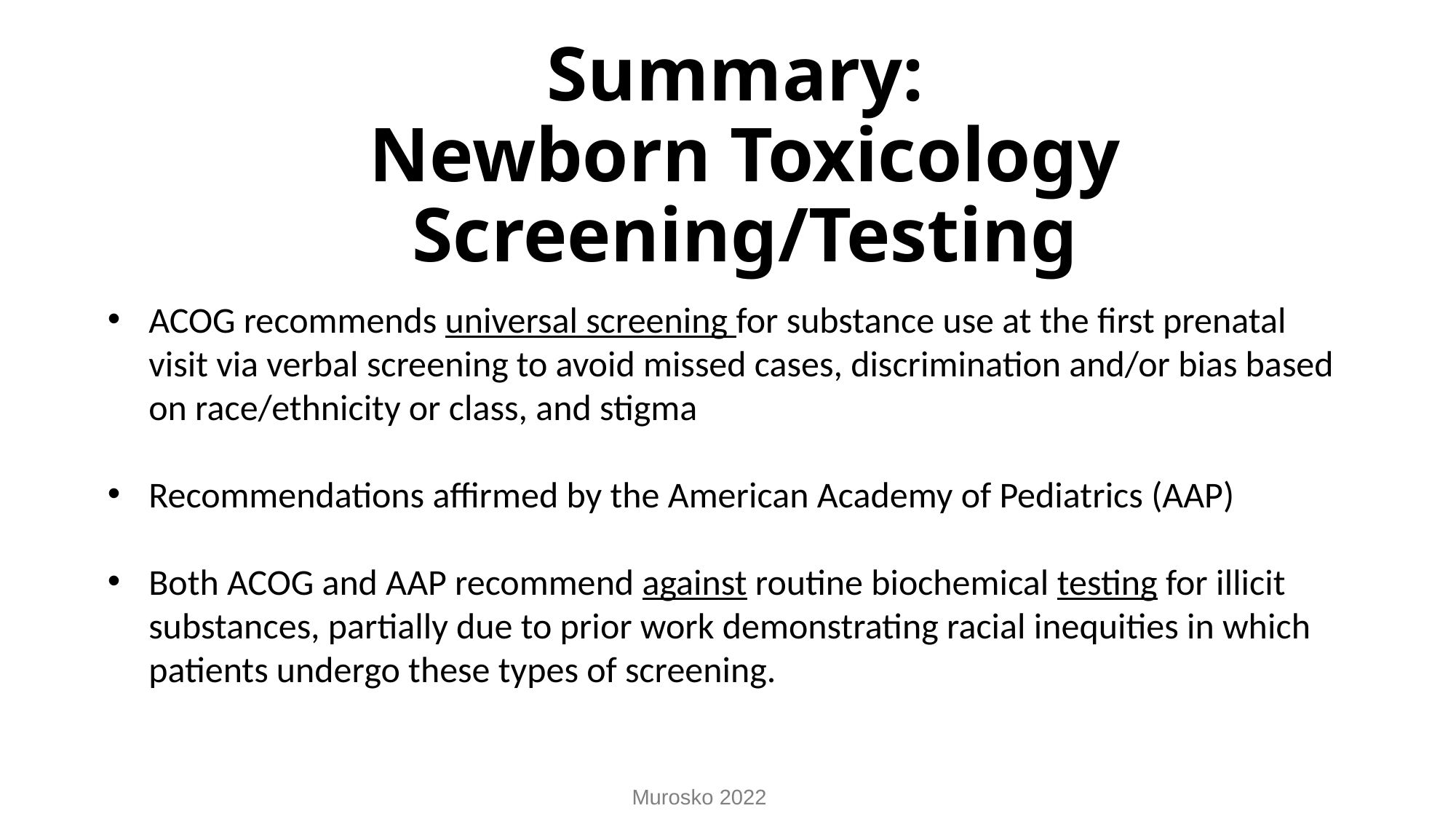

# Summary: Newborn Toxicology Screening/Testing
ACOG recommends universal screening for substance use at the first prenatal visit via verbal screening to avoid missed cases, discrimination and/or bias based on race/ethnicity or class, and stigma
Recommendations affirmed by the American Academy of Pediatrics (AAP)
Both ACOG and AAP recommend against routine biochemical testing for illicit substances, partially due to prior work demonstrating racial inequities in which patients undergo these types of screening.
Murosko 2022

## Slide 35
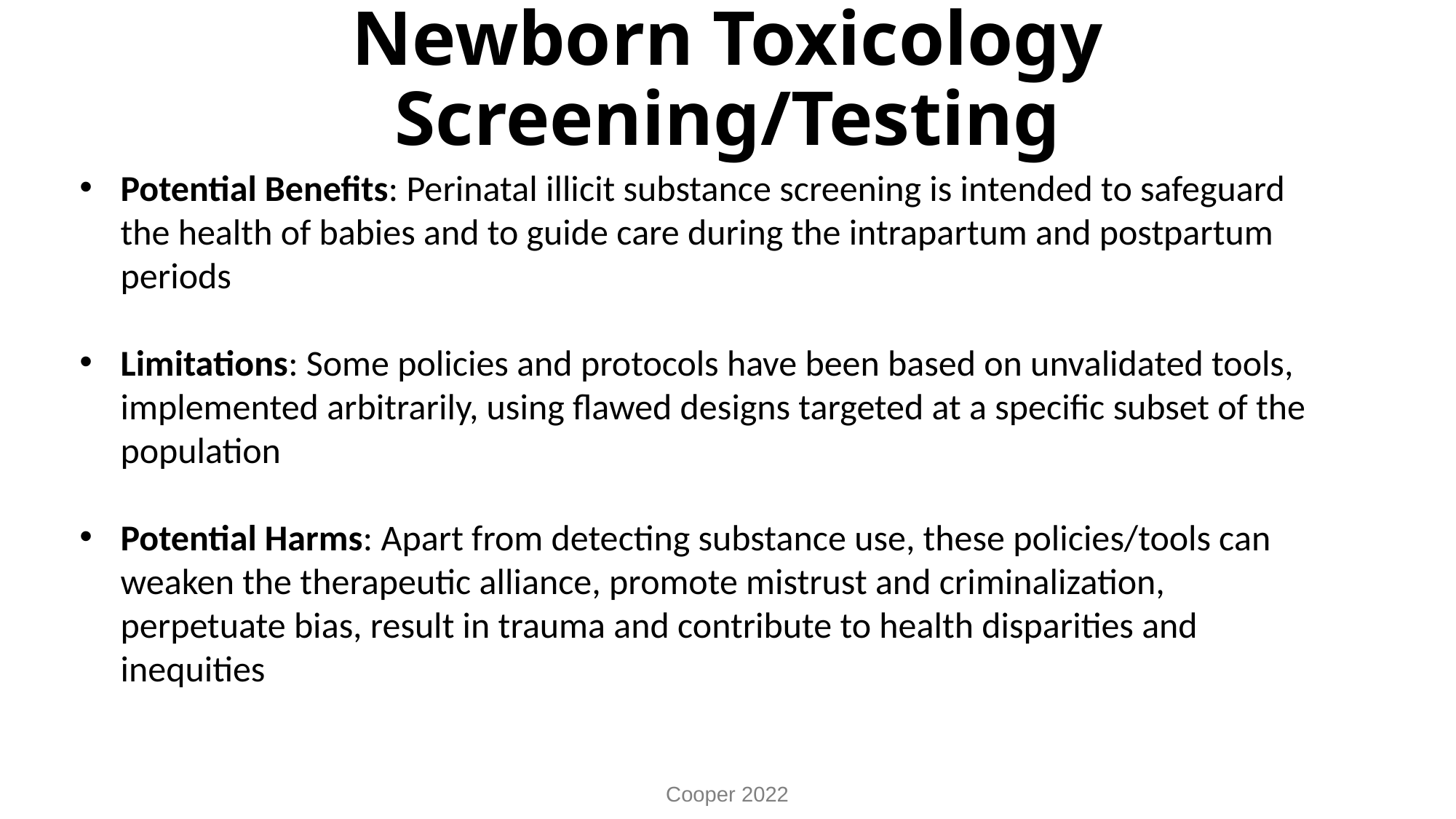

# Newborn Toxicology Screening/Testing
Potential Benefits: Perinatal illicit substance screening is intended to safeguard the health of babies and to guide care during the intrapartum and postpartum periods
Limitations: Some policies and protocols have been based on unvalidated tools, implemented arbitrarily, using flawed designs targeted at a specific subset of the population
Potential Harms: Apart from detecting substance use, these policies/tools can weaken the therapeutic alliance, promote mistrust and criminalization, perpetuate bias, result in trauma and contribute to health disparities and inequities
Cooper 2022

## Slide 36
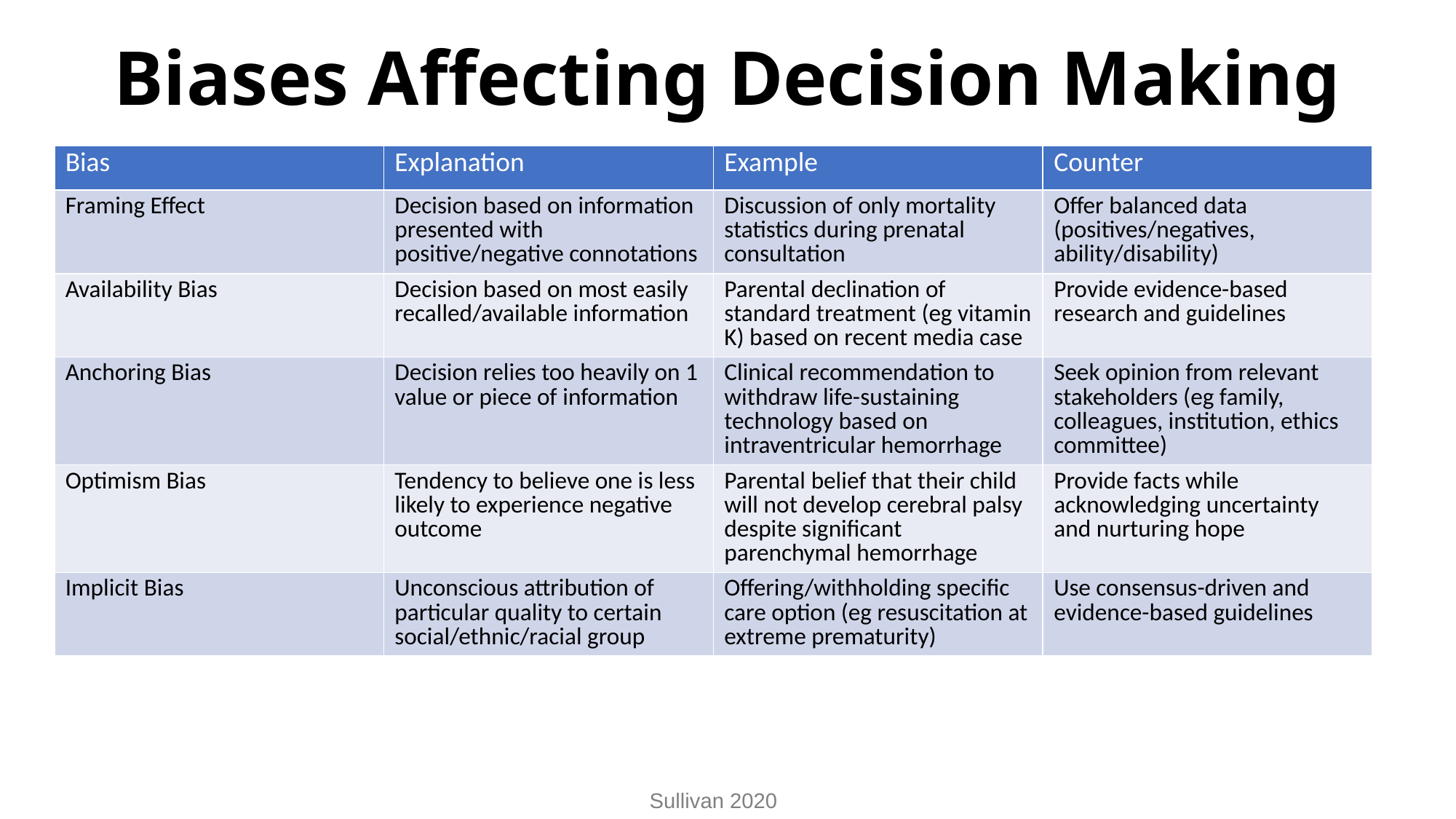

# Biases Affecting Decision Making
| Bias | Explanation | Example | Counter |
| --- | --- | --- | --- |
| Framing Effect | Decision based on information presented with positive/negative connotations | Discussion of only mortality statistics during prenatal consultation | Offer balanced data (positives/negatives, ability/disability) |
| Availability Bias | Decision based on most easily recalled/available information | Parental declination of standard treatment (eg vitamin K) based on recent media case | Provide evidence-based research and guidelines |
| Anchoring Bias | Decision relies too heavily on 1 value or piece of information | Clinical recommendation to withdraw life-sustaining technology based on intraventricular hemorrhage | Seek opinion from relevant stakeholders (eg family, colleagues, institution, ethics committee) |
| Optimism Bias | Tendency to believe one is less likely to experience negative outcome | Parental belief that their child will not develop cerebral palsy despite significant parenchymal hemorrhage | Provide facts while acknowledging uncertainty and nurturing hope |
| Implicit Bias | Unconscious attribution of particular quality to certain social/ethnic/racial group | Offering/withholding specific care option (eg resuscitation at extreme prematurity) | Use consensus-driven and evidence-based guidelines |
Sullivan 2020

## Slide 37
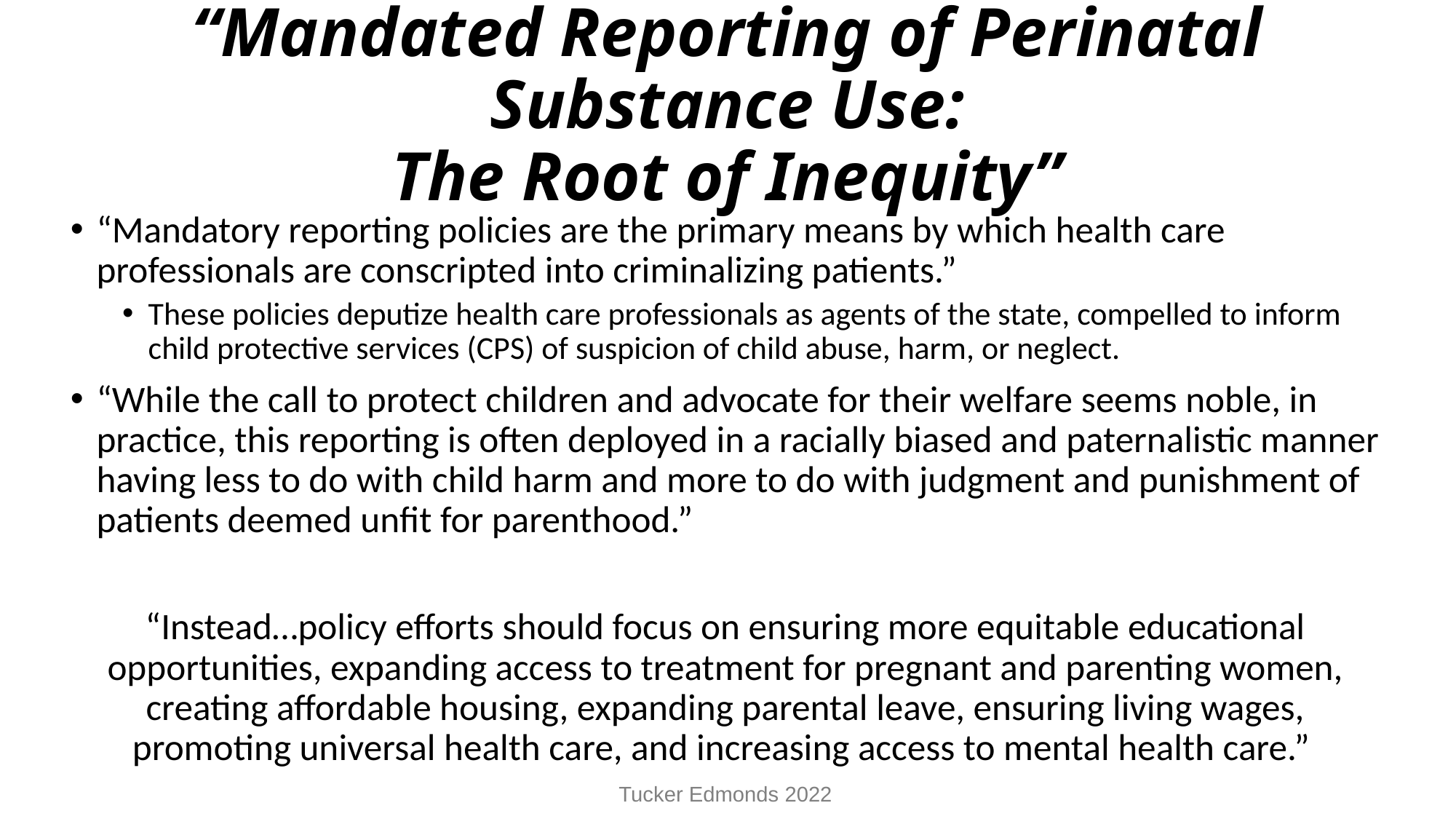

# “Mandated Reporting of Perinatal Substance Use: The Root of Inequity”
“Mandatory reporting policies are the primary means by which health care professionals are conscripted into criminalizing patients.”
These policies deputize health care professionals as agents of the state, compelled to inform child protective services (CPS) of suspicion of child abuse, harm, or neglect.
“While the call to protect children and advocate for their welfare seems noble, in practice, this reporting is often deployed in a racially biased and paternalistic manner having less to do with child harm and more to do with judgment and punishment of patients deemed unfit for parenthood.”
“Instead…policy efforts should focus on ensuring more equitable educational opportunities, expanding access to treatment for pregnant and parenting women, creating affordable housing, expanding parental leave, ensuring living wages, promoting universal health care, and increasing access to mental health care.”
Tucker Edmonds 2022

## Slide 38
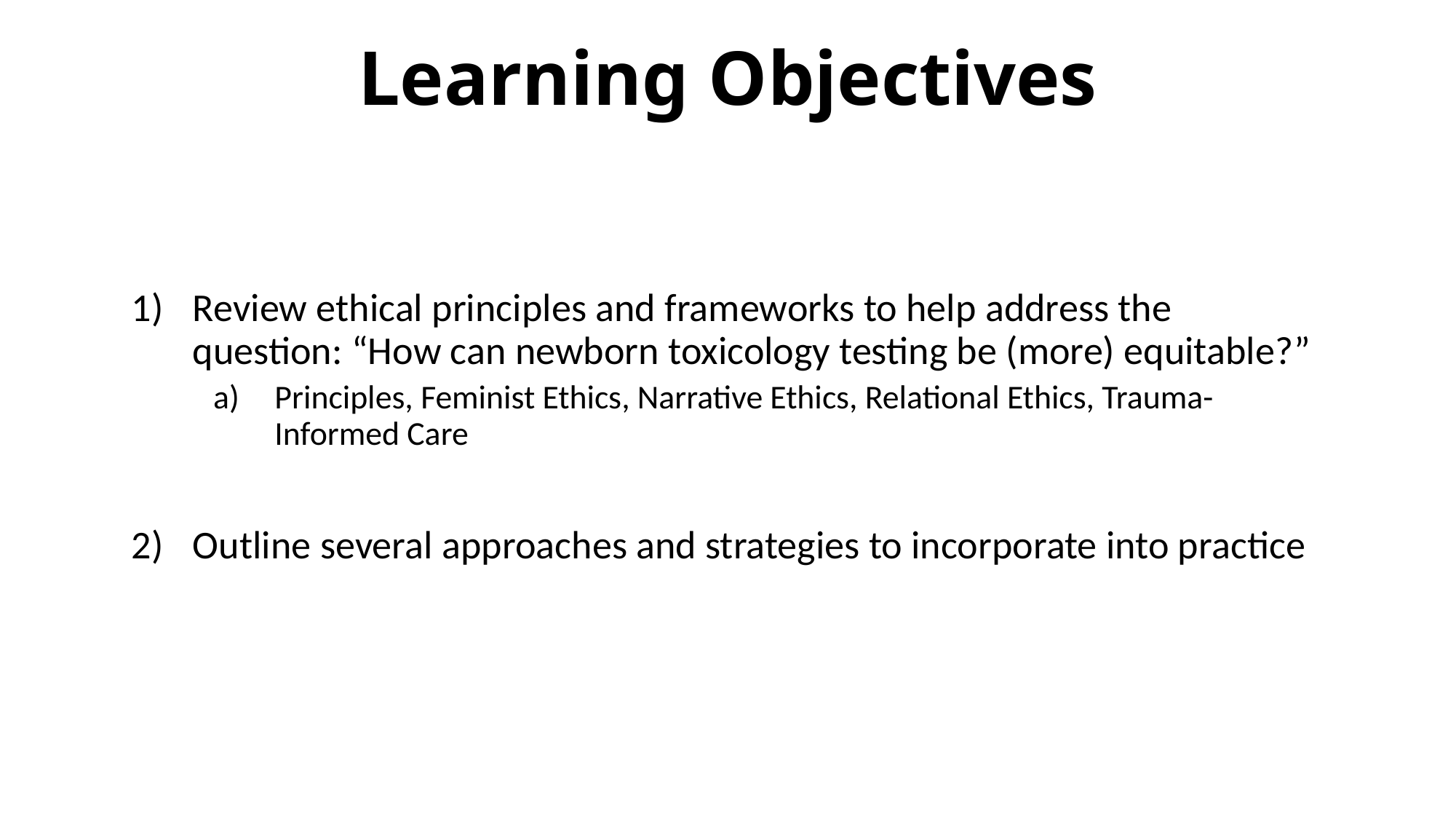

# Learning Objectives
Review ethical principles and frameworks to help address the question: “How can newborn toxicology testing be (more) equitable?”
Principles, Feminist Ethics, Narrative Ethics, Relational Ethics, Trauma-Informed Care
Outline several approaches and strategies to incorporate into practice

## Slide 39
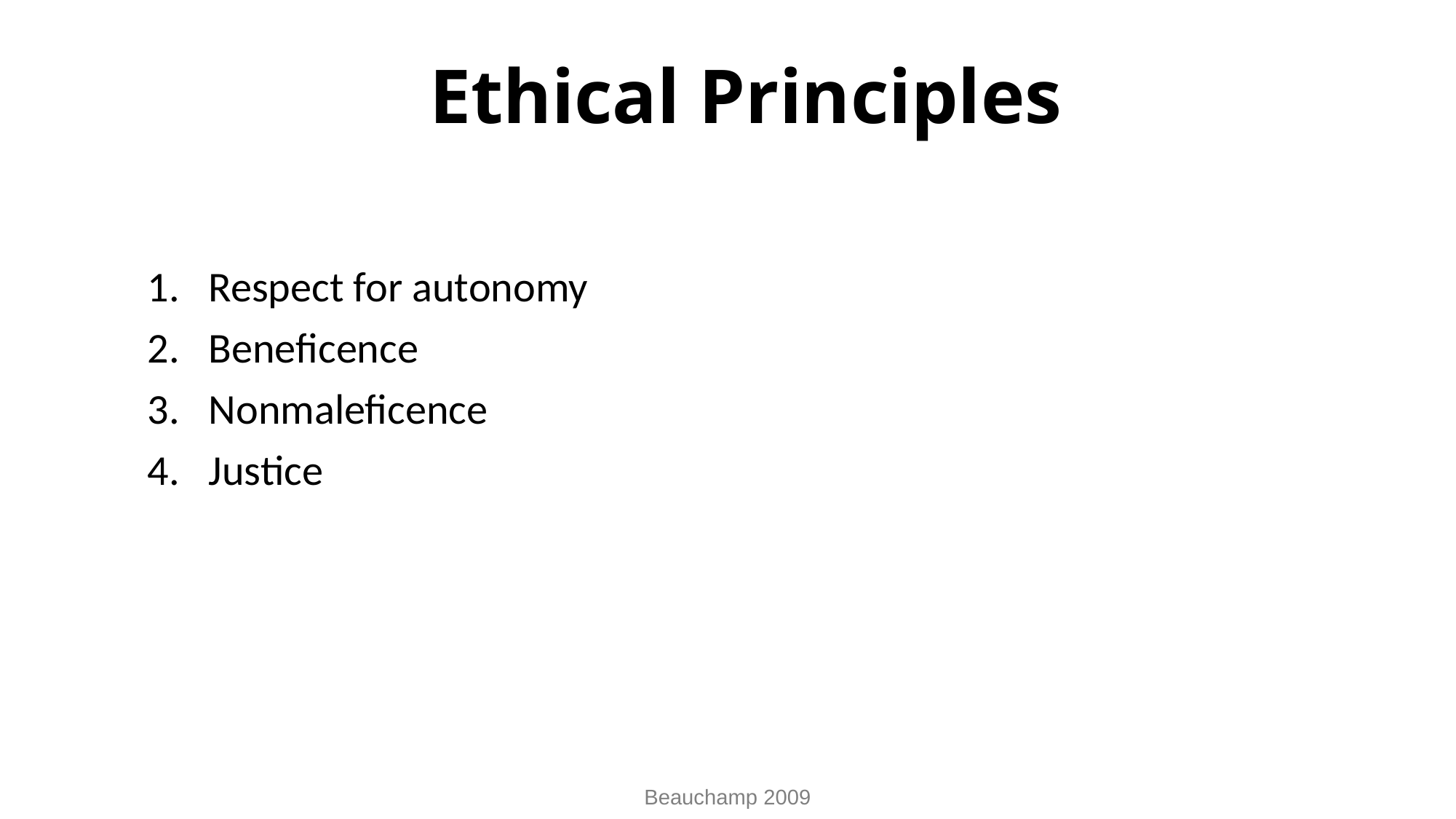

Ethical Principles
Respect for autonomy
Beneficence
Nonmaleficence
Justice
Beauchamp 2009

## Slide 40
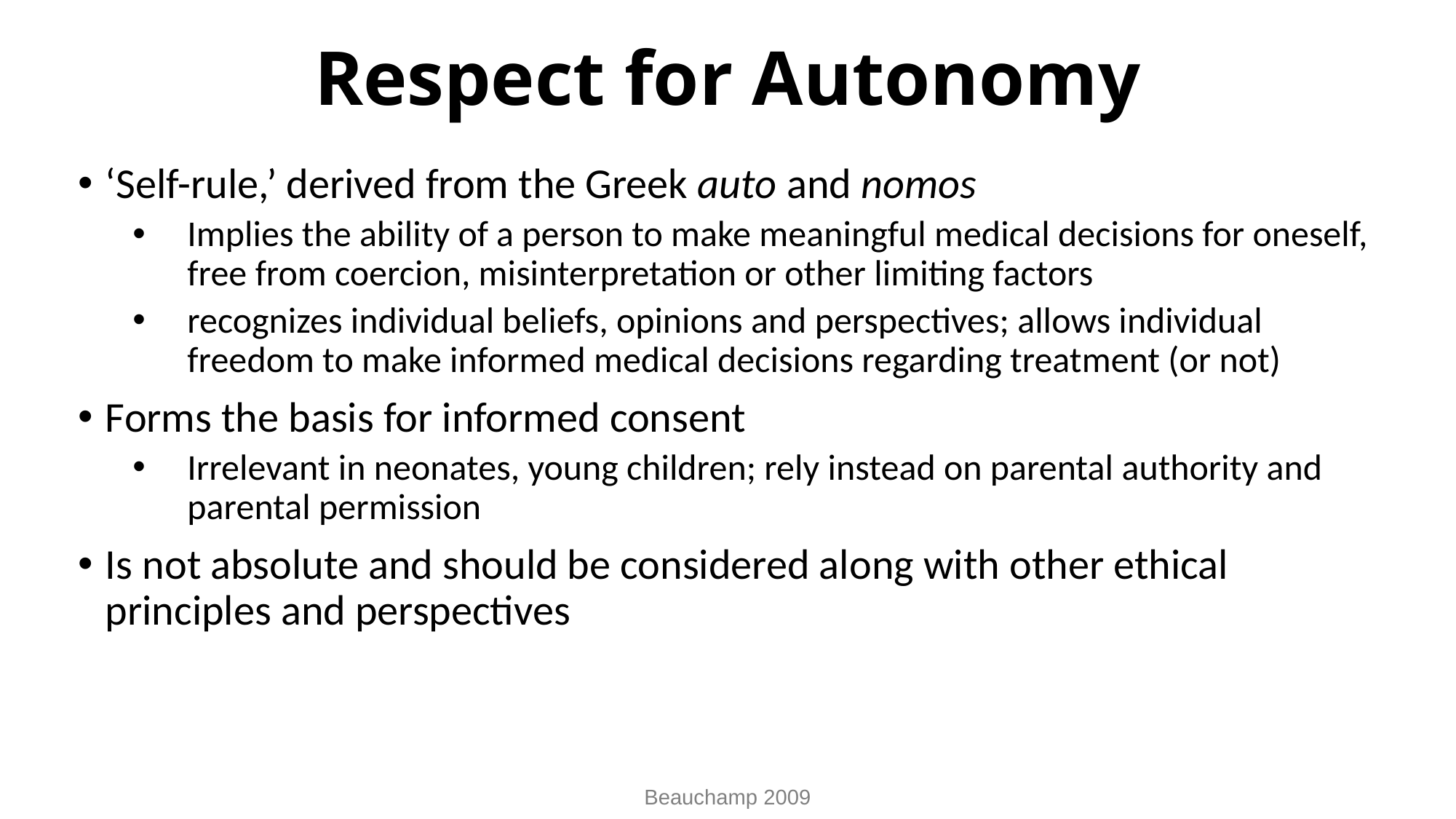

# Respect for Autonomy
‘Self-rule,’ derived from the Greek auto and nomos
Implies the ability of a person to make meaningful medical decisions for oneself, free from coercion, misinterpretation or other limiting factors
recognizes individual beliefs, opinions and perspectives; allows individual freedom to make informed medical decisions regarding treatment (or not)
Forms the basis for informed consent
Irrelevant in neonates, young children; rely instead on parental authority and parental permission
Is not absolute and should be considered along with other ethical principles and perspectives
Beauchamp 2009

## Slide 41
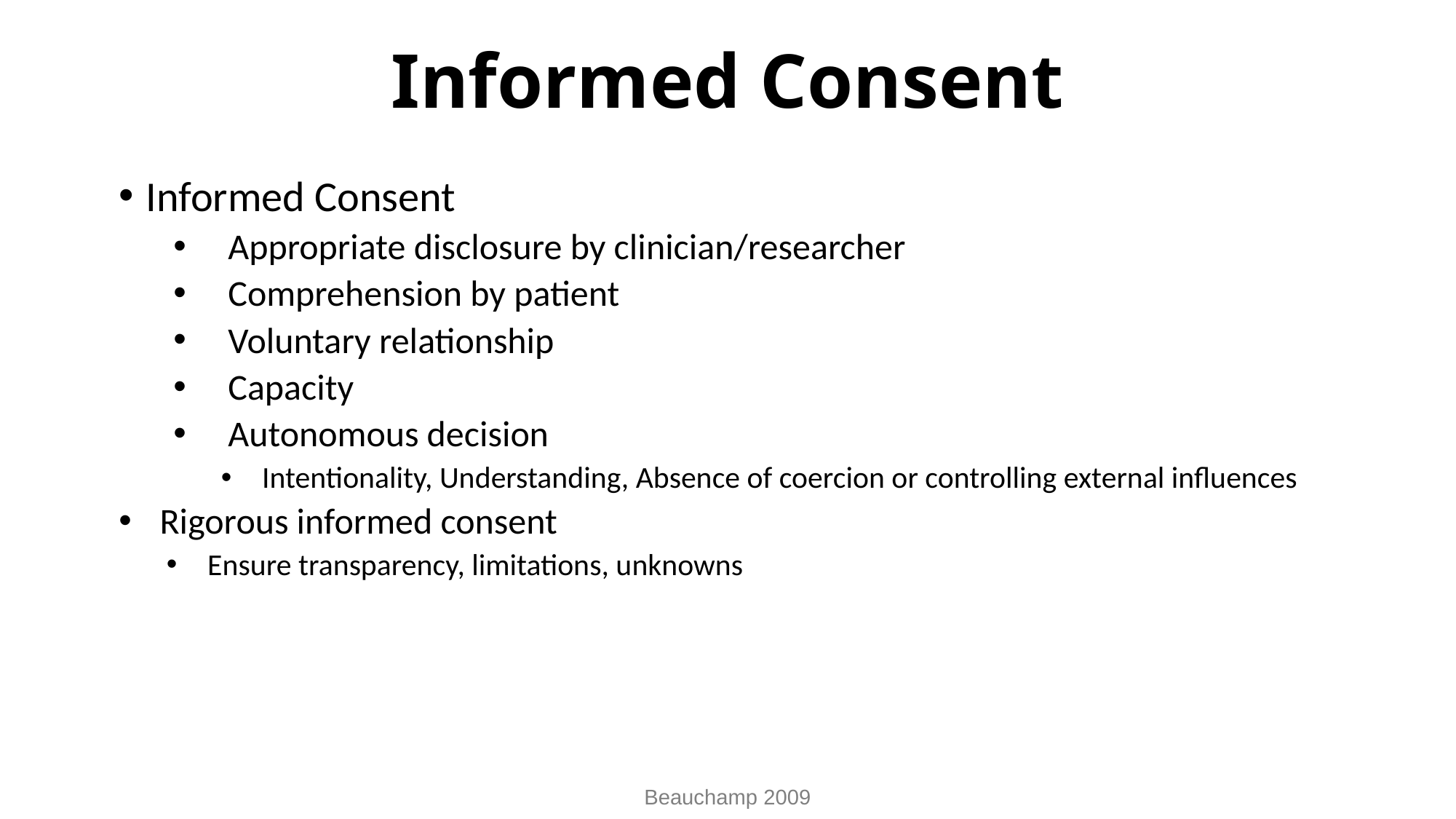

# Informed Consent
Informed Consent
Appropriate disclosure by clinician/researcher
Comprehension by patient
Voluntary relationship
Capacity
Autonomous decision
Intentionality, Understanding, Absence of coercion or controlling external influences
Rigorous informed consent
Ensure transparency, limitations, unknowns
Beauchamp 2009

## Slide 42
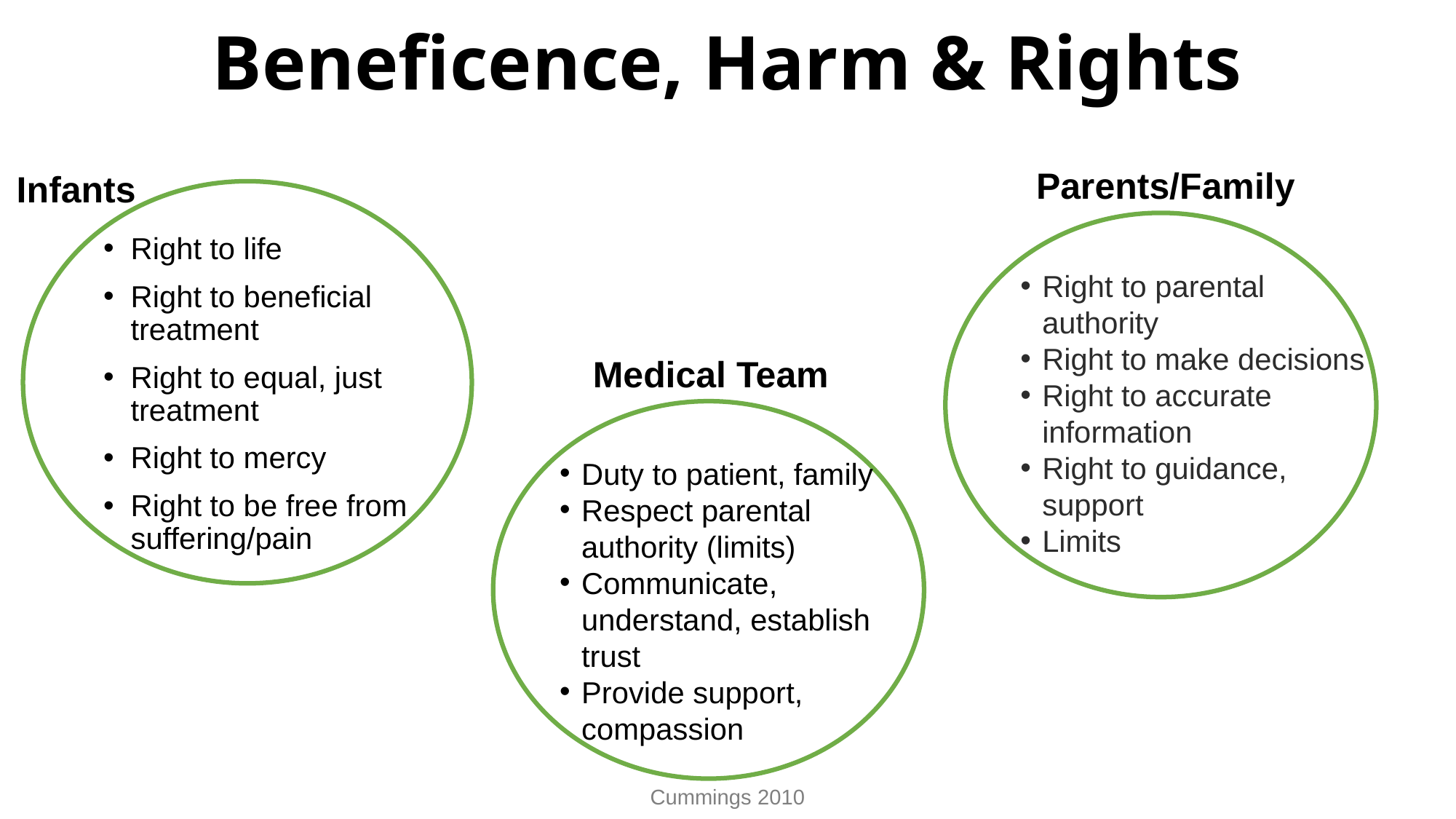

# Beneficence, Harm & Rights
Parents/Family
Infants
Right to life
Right to beneficial treatment
Right to equal, just treatment
Right to mercy
Right to be free from suffering/pain
Right to parental authority
Right to make decisions
Right to accurate information
Right to guidance, support
Limits
Medical Team
Duty to patient, family
Respect parental authority (limits)
Communicate, understand, establish trust
Provide support, compassion
Cummings 2010

## Slide 43
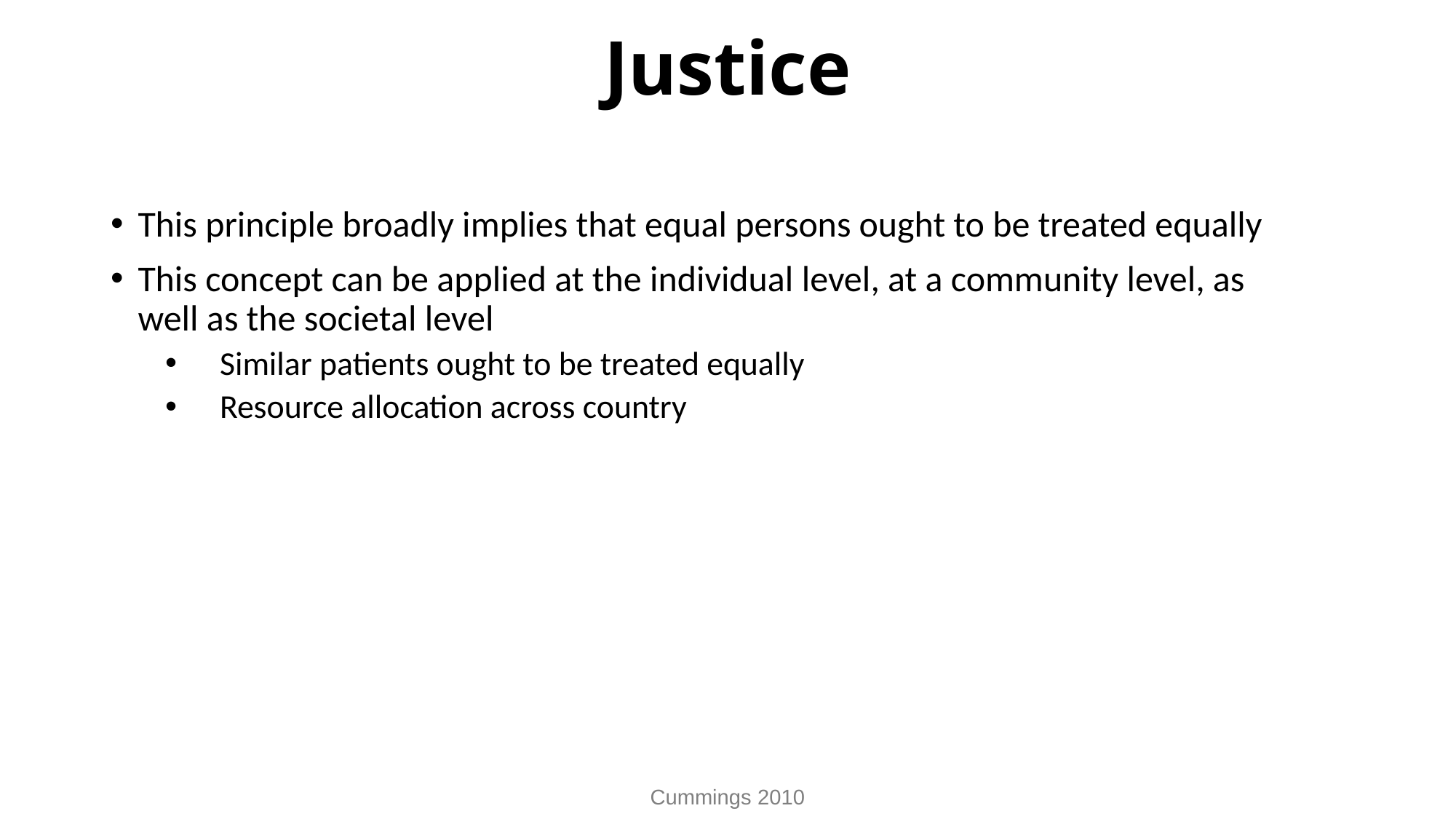

# Justice
This principle broadly implies that equal persons ought to be treated equally
This concept can be applied at the individual level, at a community level, as well as the societal level
Similar patients ought to be treated equally
Resource allocation across country
Cummings 2010

## Slide 44
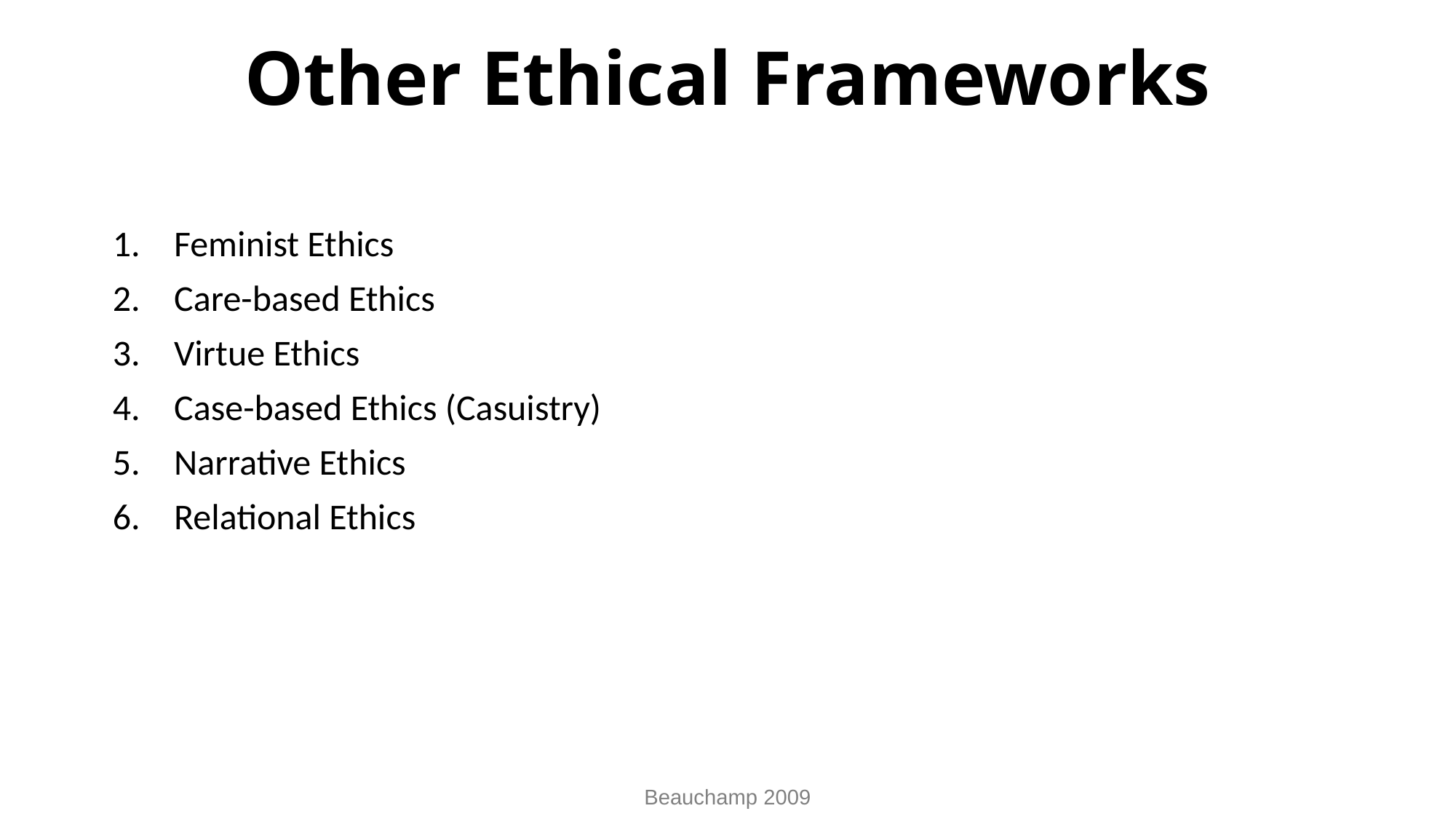

# Other Ethical Frameworks
Feminist Ethics
Care-based Ethics
Virtue Ethics
Case-based Ethics (Casuistry)
Narrative Ethics
Relational Ethics
Beauchamp 2009

## Slide 45
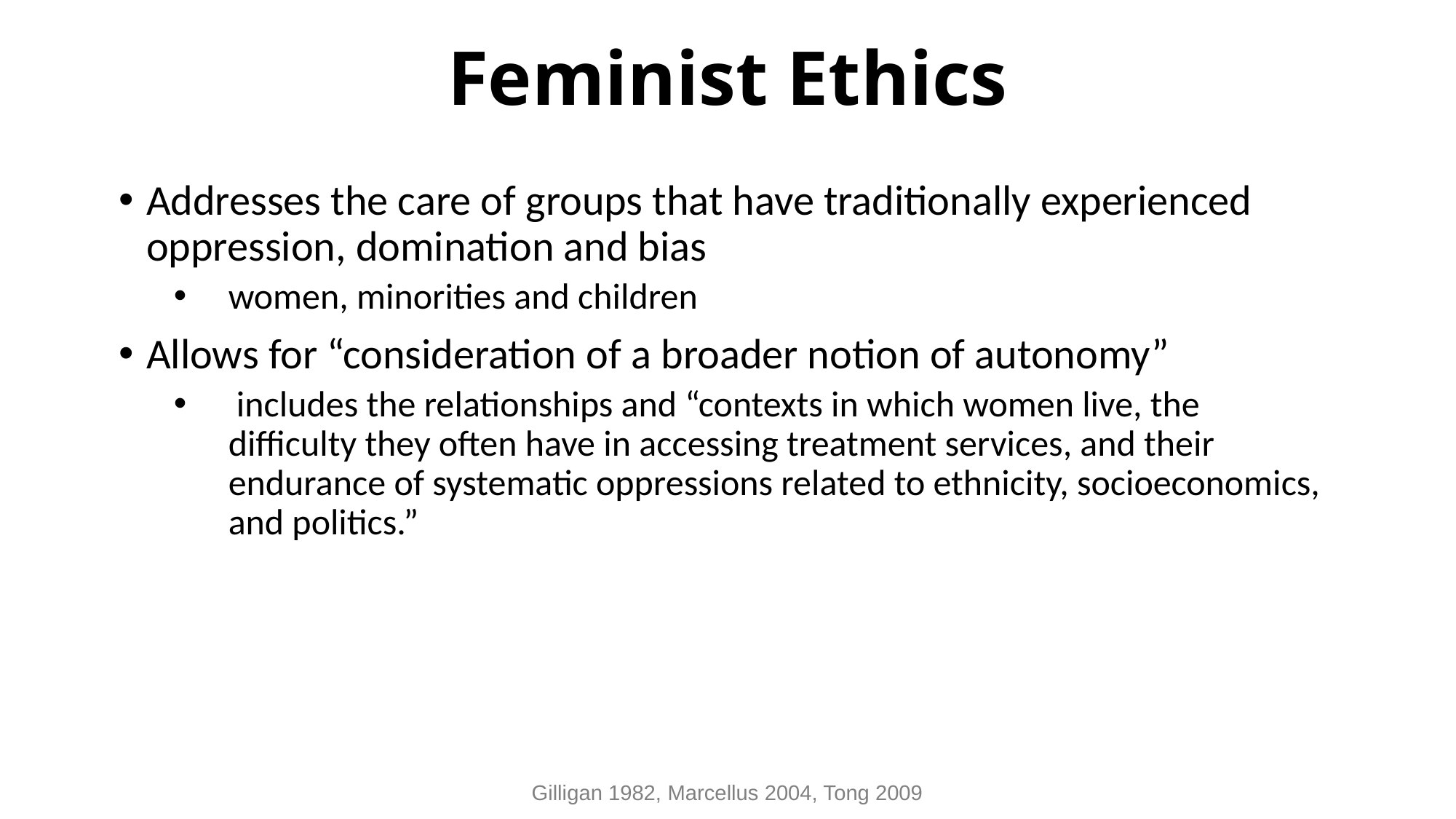

# Feminist Ethics
Addresses the care of groups that have traditionally experienced oppression, domination and bias
women, minorities and children
Allows for “consideration of a broader notion of autonomy”
 includes the relationships and “contexts in which women live, the difficulty they often have in accessing treatment services, and their endurance of systematic oppressions related to ethnicity, socioeconomics, and politics.”
Gilligan 1982, Marcellus 2004, Tong 2009

## Slide 46
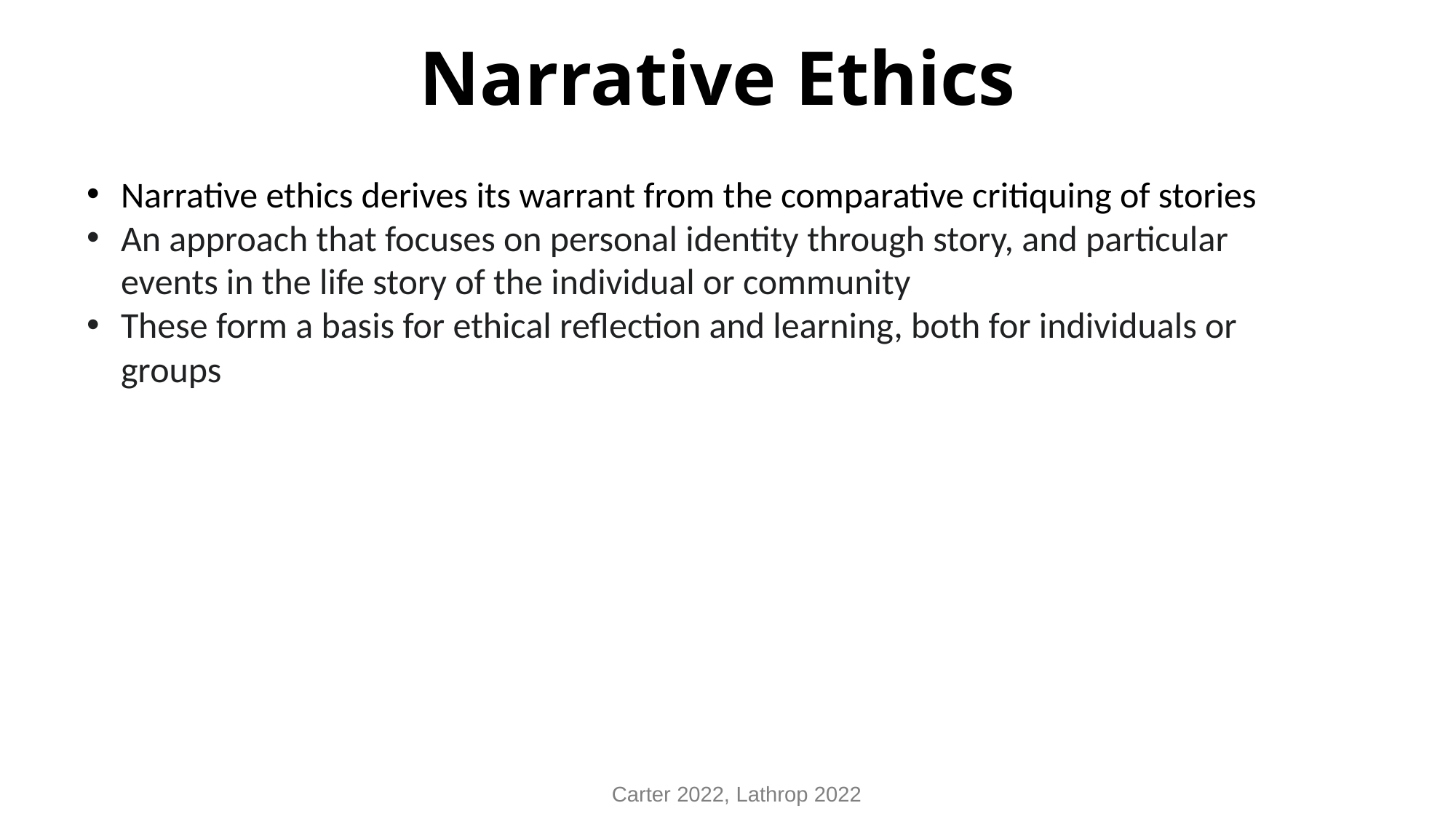

# Narrative Ethics
Narrative ethics derives its warrant from the comparative critiquing of stories
An approach that focuses on personal identity through story, and particular events in the life story of the individual or community
These form a basis for ethical reflection and learning, both for individuals or groups
Carter 2022, Lathrop 2022

## Slide 47
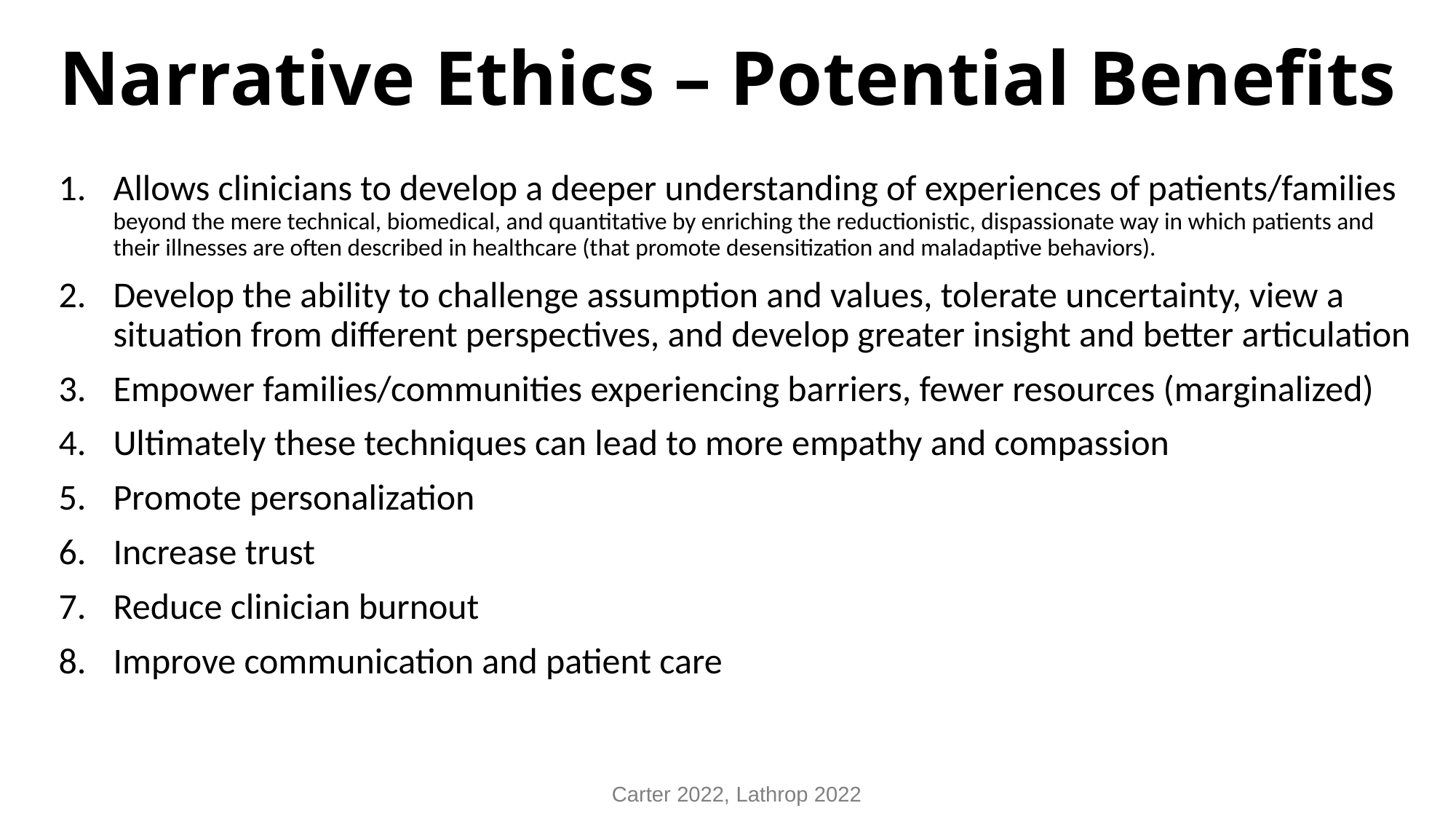

# Narrative Ethics – Potential Benefits
Allows clinicians to develop a deeper understanding of experiences of patients/families beyond the mere technical, biomedical, and quantitative by enriching the reductionistic, dispassionate way in which patients and their illnesses are often described in healthcare (that promote desensitization and maladaptive behaviors).
Develop the ability to challenge assumption and values, tolerate uncertainty, view a situation from different perspectives, and develop greater insight and better articulation
Empower families/communities experiencing barriers, fewer resources (marginalized)
Ultimately these techniques can lead to more empathy and compassion
Promote personalization
Increase trust
Reduce clinician burnout
Improve communication and patient care
Carter 2022, Lathrop 2022

## Slide 48
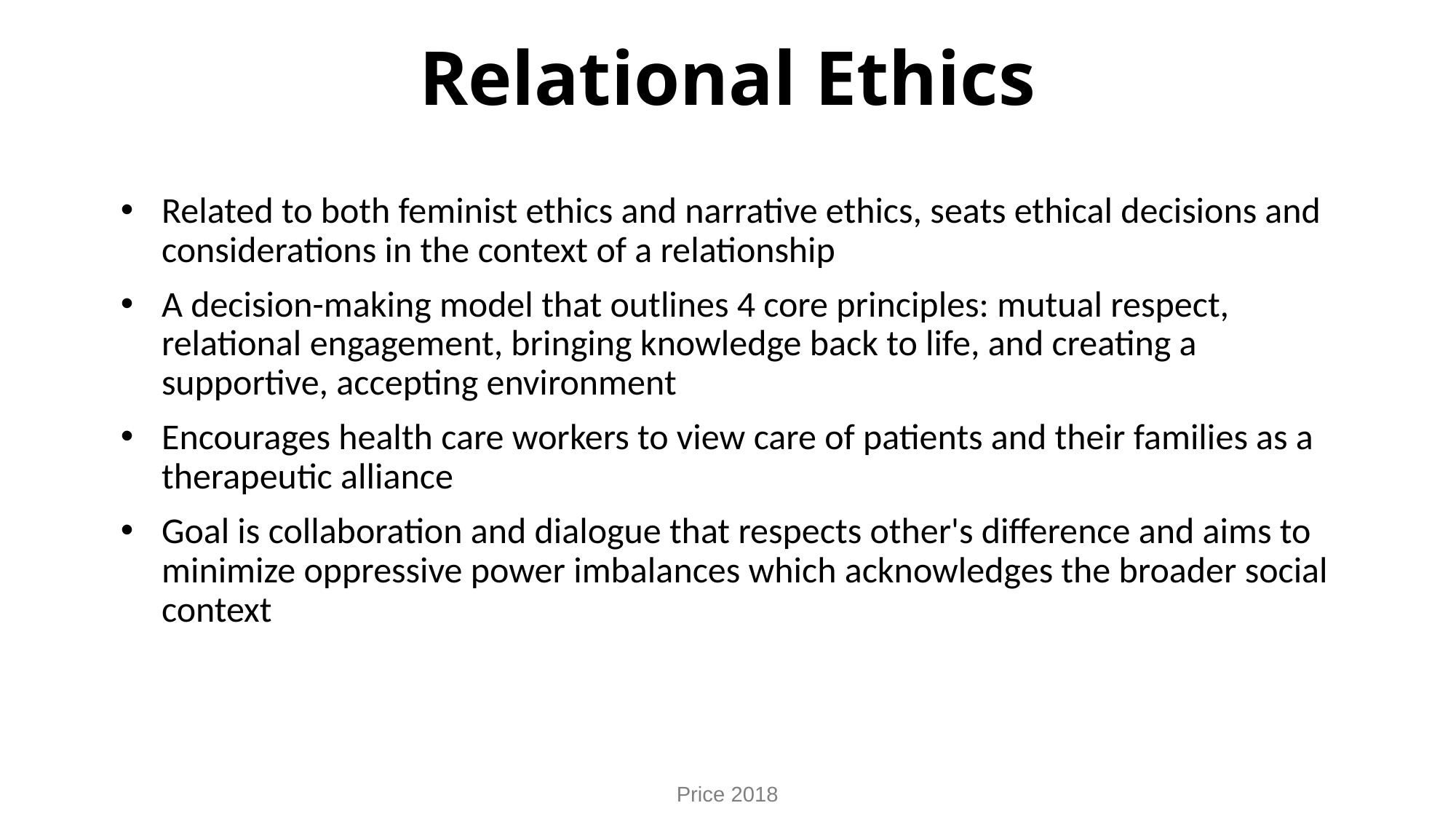

# Relational Ethics
Related to both feminist ethics and narrative ethics, seats ethical decisions and considerations in the context of a relationship
A decision-making model that outlines 4 core principles: mutual respect, relational engagement, bringing knowledge back to life, and creating a supportive, accepting environment
Encourages health care workers to view care of patients and their families as a therapeutic alliance
Goal is collaboration and dialogue that respects other's difference and aims to minimize oppressive power imbalances which acknowledges the broader social context
Price 2018

## Slide 49
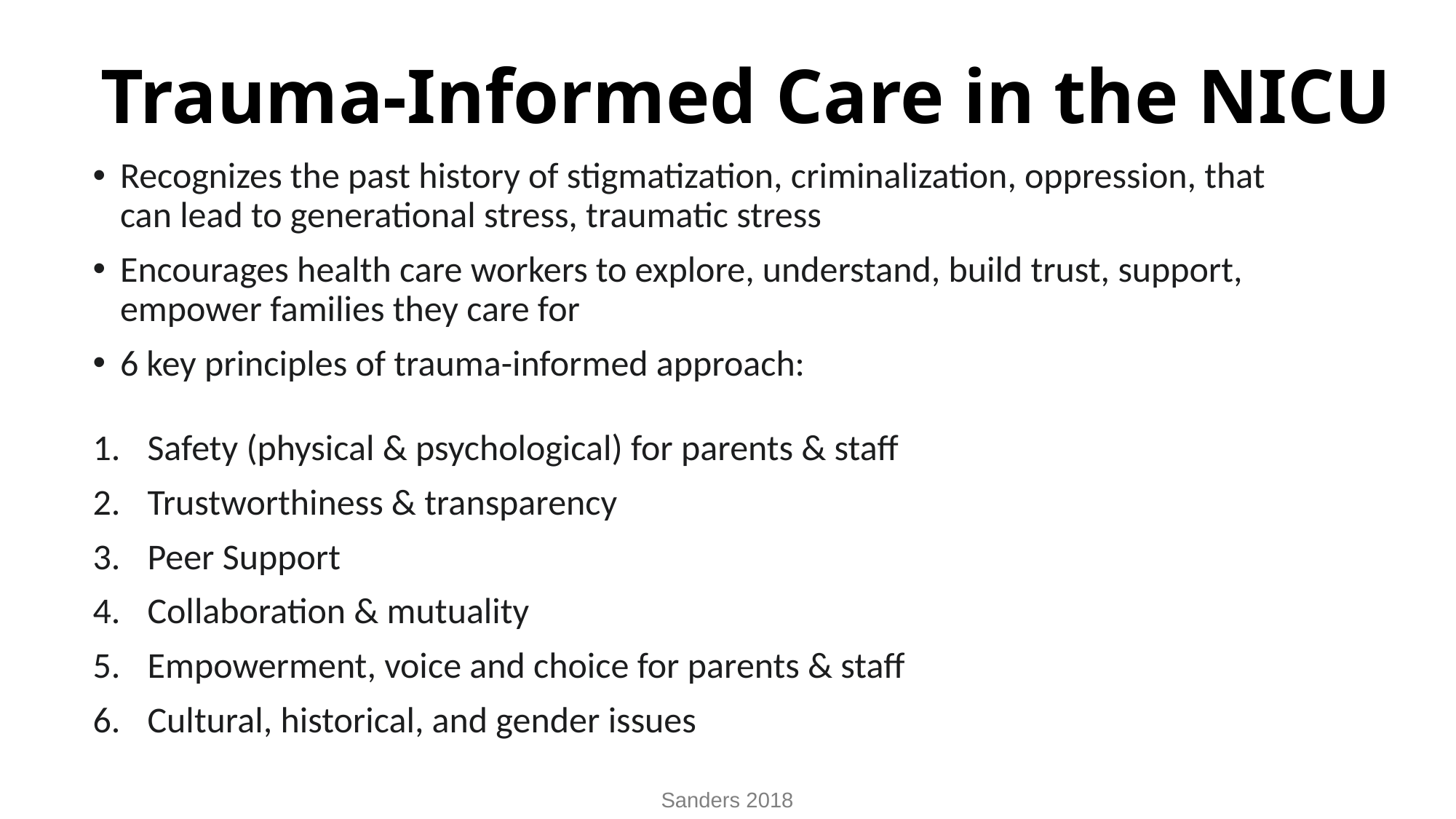

# Traumatic Stress and Trauma-Informed Care
Trauma-Informed Care in the NICU
Recognizes the past history of stigmatization, criminalization, oppression, that can lead to generational stress, traumatic stress
Encourages health care workers to explore, understand, build trust, support, empower families they care for
6 key principles of trauma-informed approach:
Safety (physical & psychological) for parents & staff
Trustworthiness & transparency
Peer Support
Collaboration & mutuality
Empowerment, voice and choice for parents & staff
Cultural, historical, and gender issues
Sanders 2018

## Slide 50
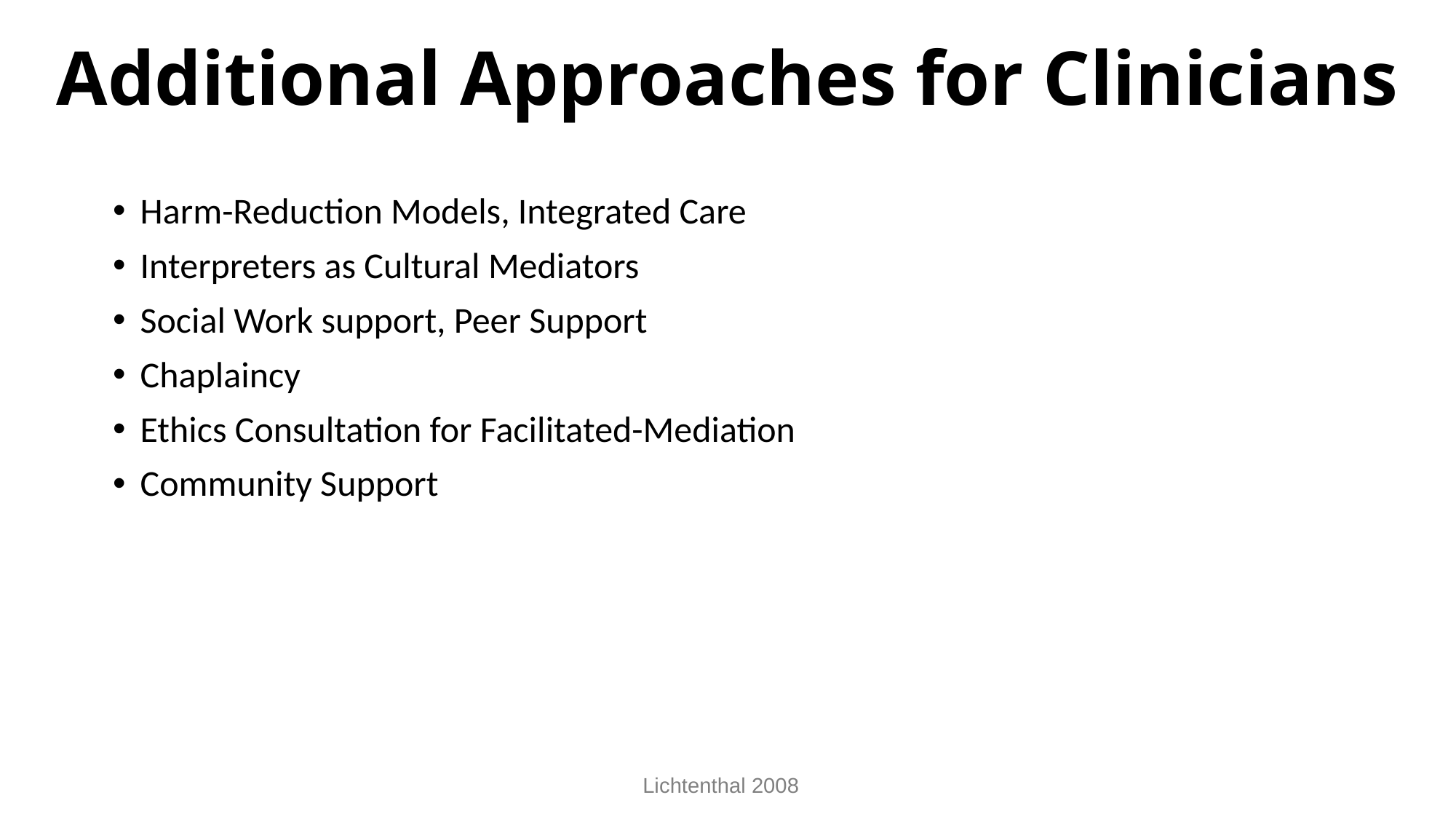

# Additional Approaches for Clinicians
Harm-Reduction Models, Integrated Care
Interpreters as Cultural Mediators
Social Work support, Peer Support
Chaplaincy
Ethics Consultation for Facilitated-Mediation
Community Support
Lichtenthal 2008

## Slide 51
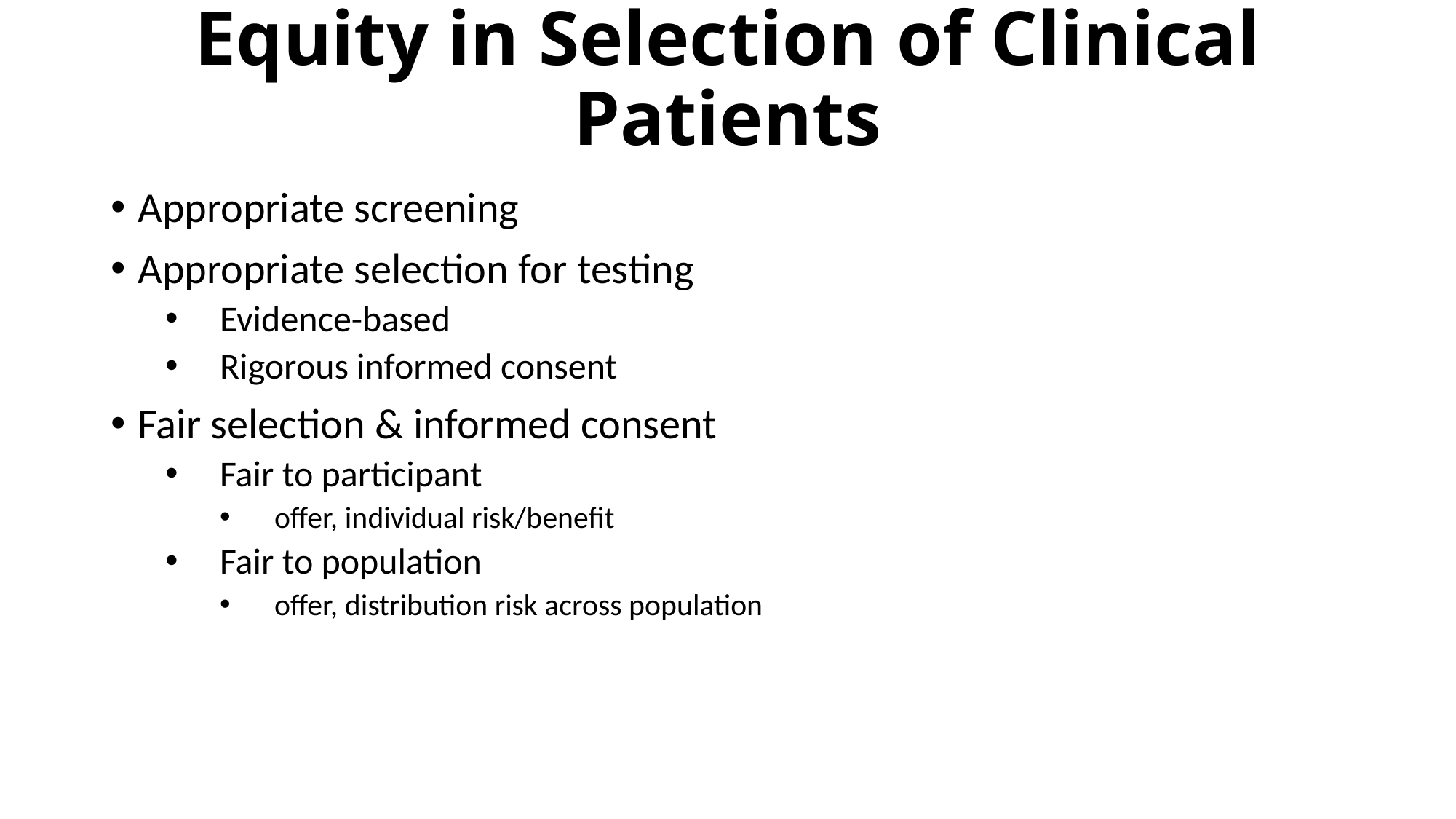

# Equity in Selection of Clinical Patients
Appropriate screening
Appropriate selection for testing
Evidence-based
Rigorous informed consent
Fair selection & informed consent
Fair to participant
offer, individual risk/benefit
Fair to population
offer, distribution risk across population

## Slide 52
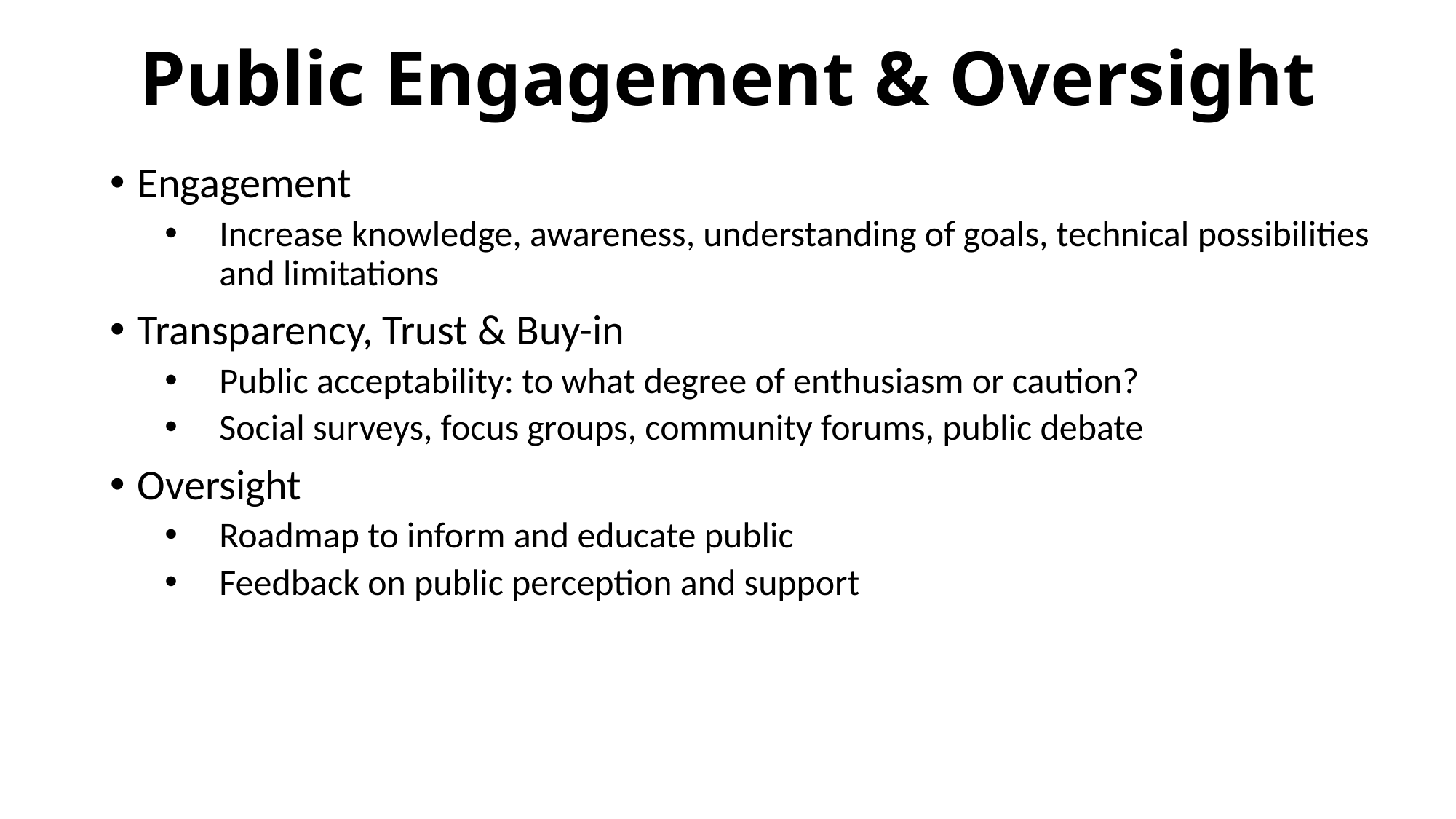

# Public Engagement & Oversight
Engagement
Increase knowledge, awareness, understanding of goals, technical possibilities and limitations
Transparency, Trust & Buy-in
Public acceptability: to what degree of enthusiasm or caution?
Social surveys, focus groups, community forums, public debate
Oversight
Roadmap to inform and educate public
Feedback on public perception and support

## Slide 53
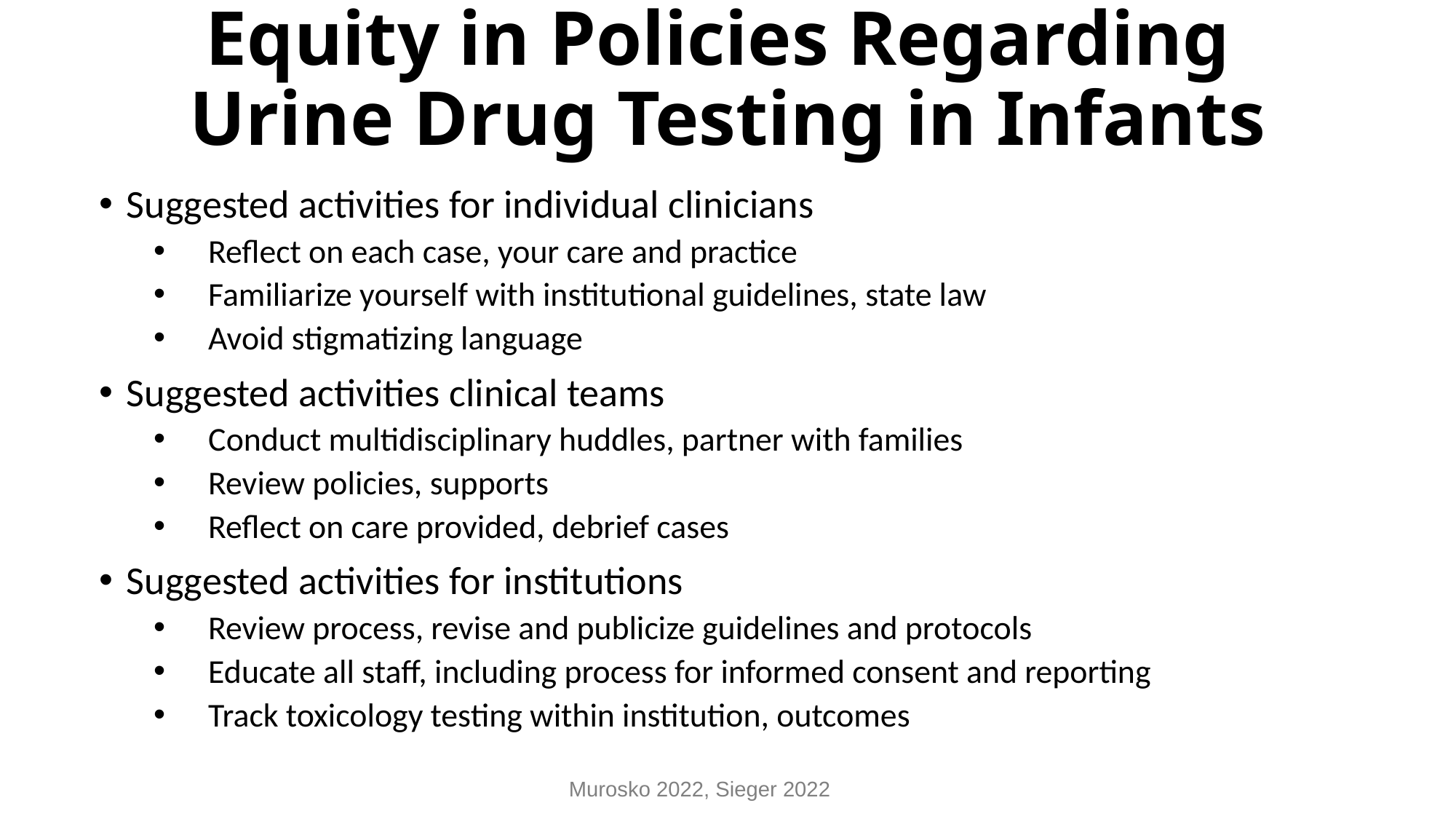

# Equity in Policies Regarding Urine Drug Testing in Infants
Suggested activities for individual clinicians
Reflect on each case, your care and practice
Familiarize yourself with institutional guidelines, state law
Avoid stigmatizing language
Suggested activities clinical teams
Conduct multidisciplinary huddles, partner with families
Review policies, supports
Reflect on care provided, debrief cases
Suggested activities for institutions
Review process, revise and publicize guidelines and protocols
Educate all staff, including process for informed consent and reporting
Track toxicology testing within institution, outcomes
Murosko 2022, Sieger 2022

## Slide 54
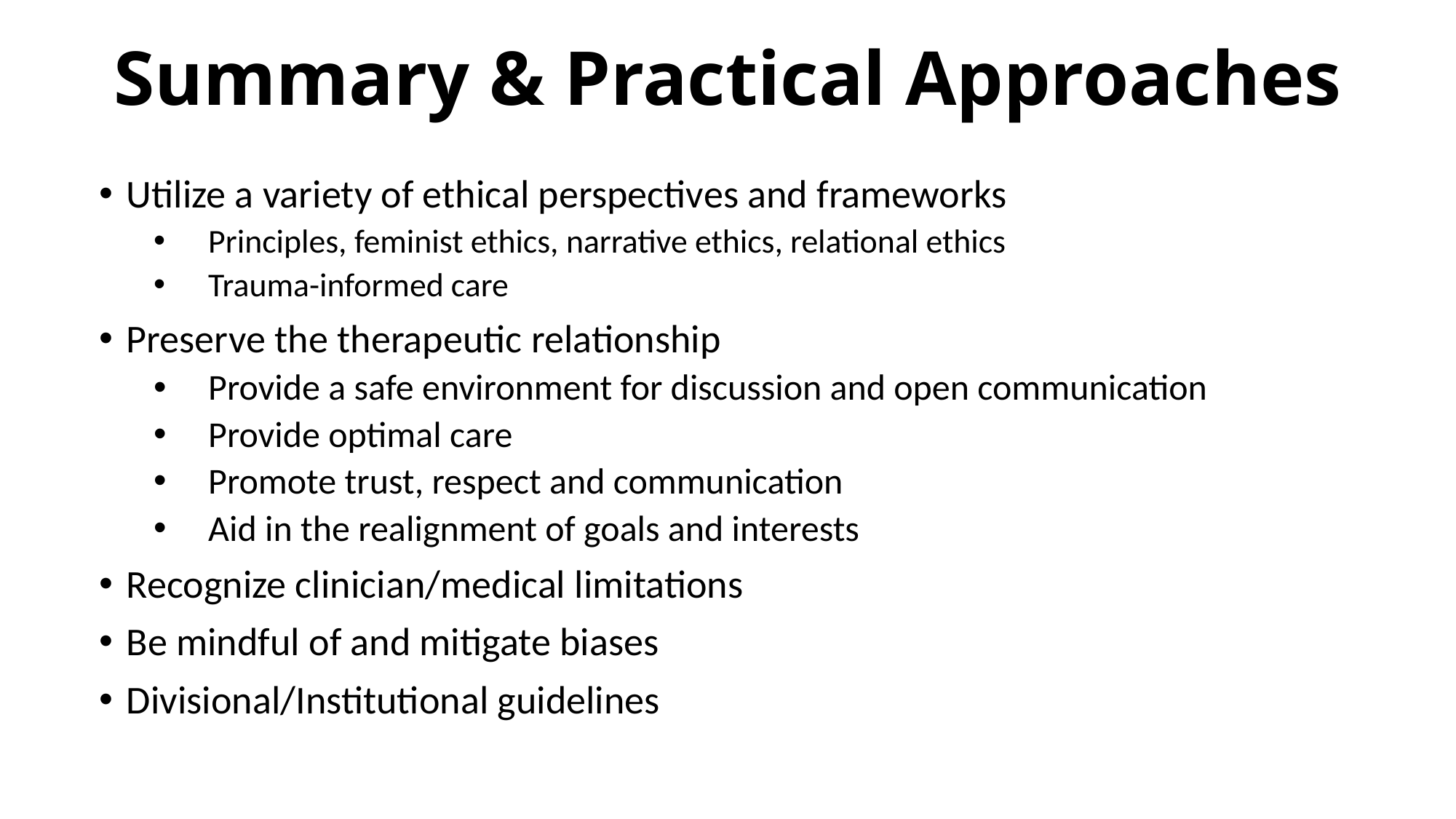

# Summary & Practical Approaches
Utilize a variety of ethical perspectives and frameworks
Principles, feminist ethics, narrative ethics, relational ethics
Trauma-informed care
Preserve the therapeutic relationship
Provide a safe environment for discussion and open communication
Provide optimal care
Promote trust, respect and communication
Aid in the realignment of goals and interests
Recognize clinician/medical limitations
Be mindful of and mitigate biases
Divisional/Institutional guidelines

## Slide 55
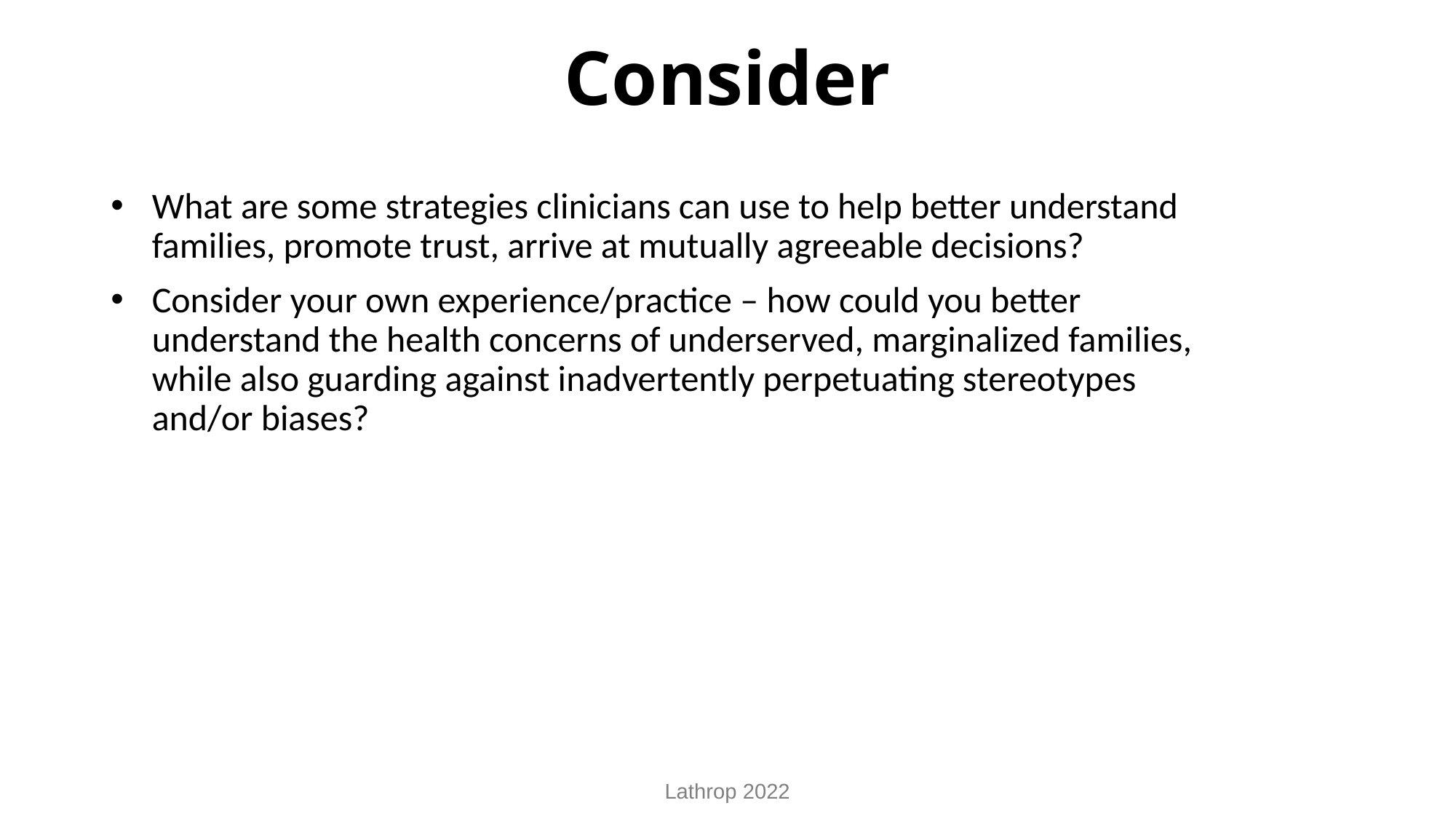

# Consider
What are some strategies clinicians can use to help better understand families, promote trust, arrive at mutually agreeable decisions?
Consider your own experience/practice – how could you better understand the health concerns of underserved, marginalized families, while also guarding against inadvertently perpetuating stereotypes and/or biases?
Lathrop 2022

## Slide 56
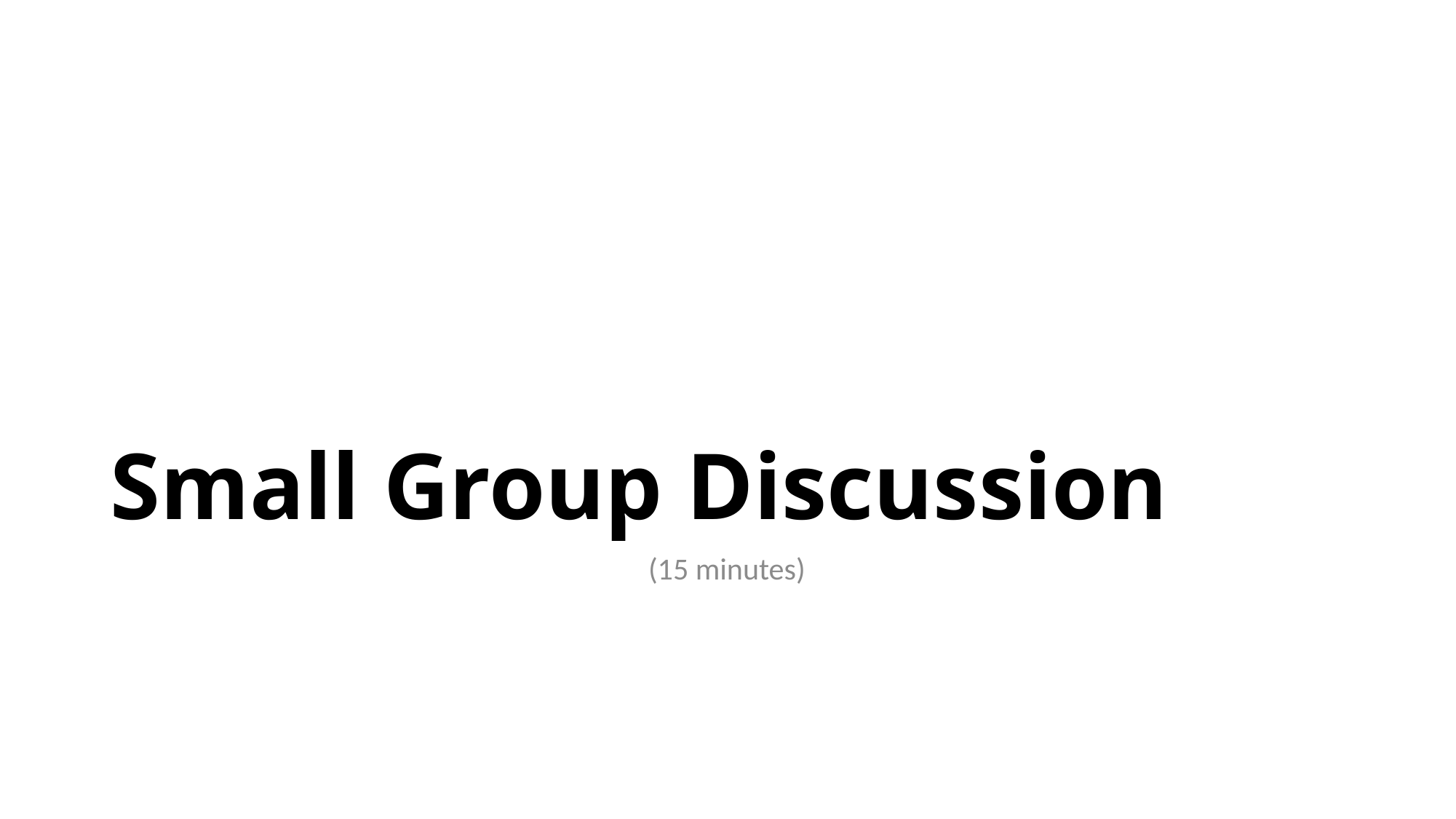

# Small Group Discussion
(15 minutes)

## Slide 57
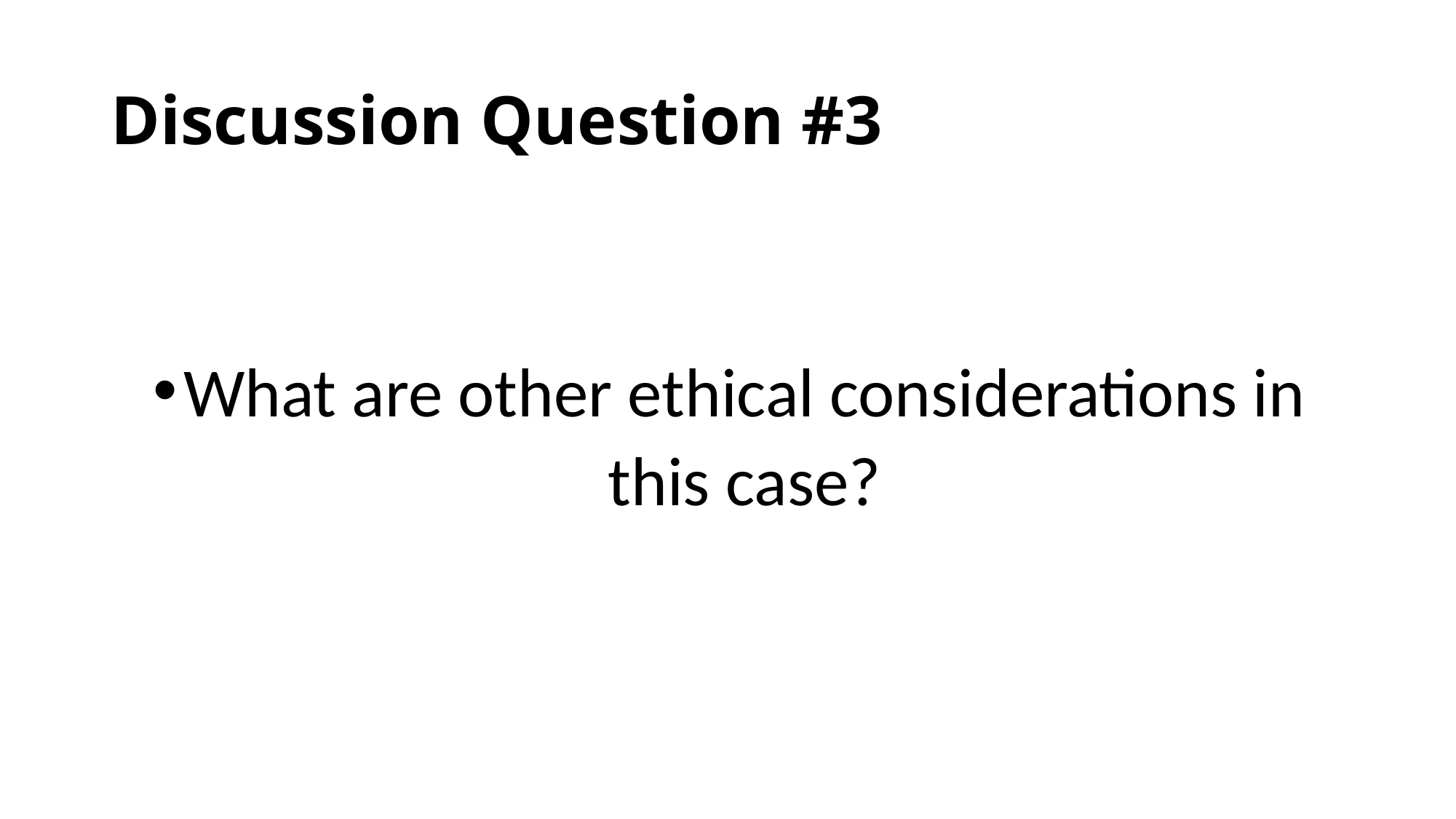

# Discussion Question #3
What are other ethical considerations in this case?

## Slide 58
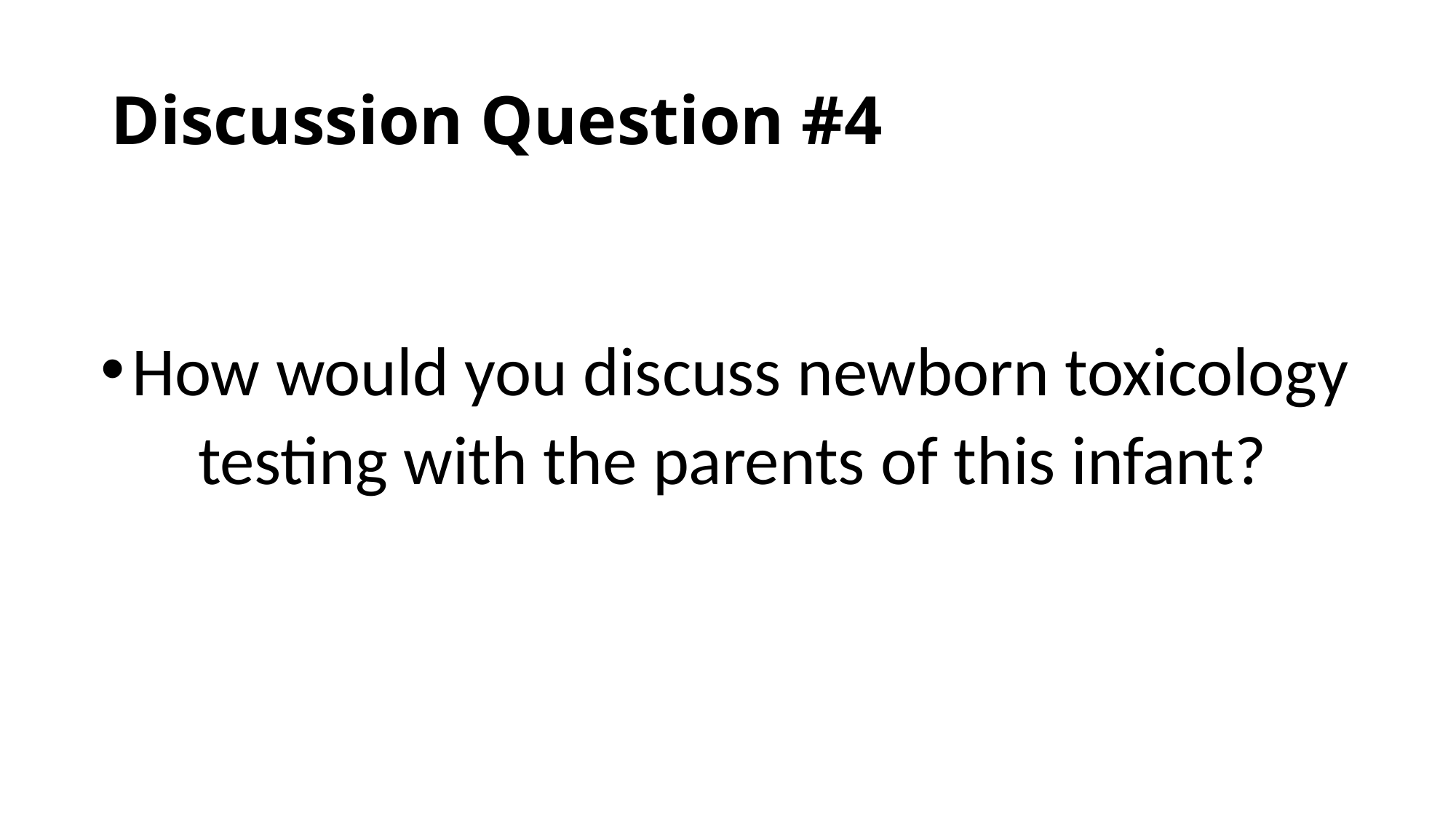

# Discussion Question #4
How would you discuss newborn toxicology testing with the parents of this infant?

## Slide 59
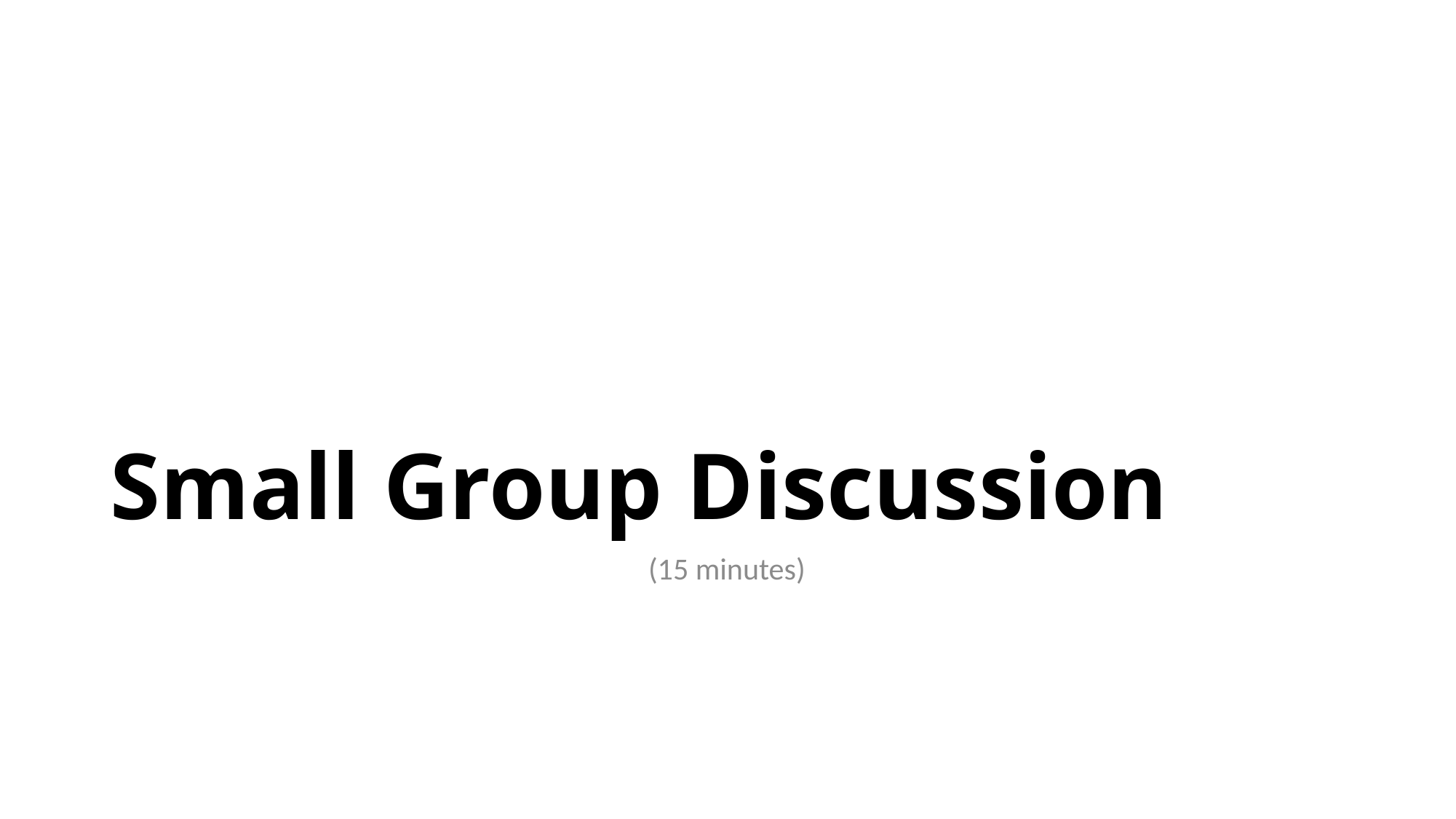

# Small Group Discussion
(15 minutes)

## Slide 60
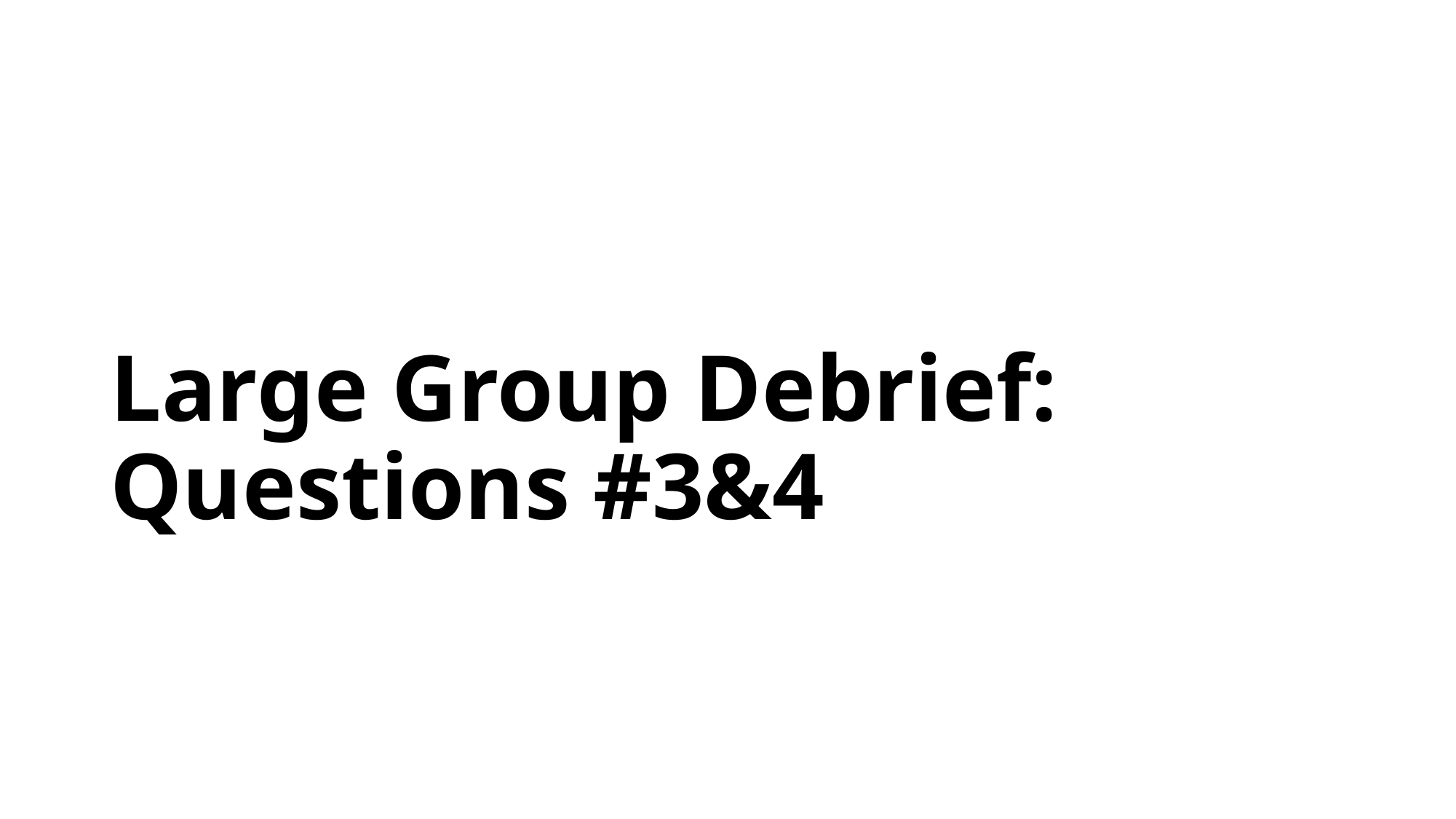

# Large Group Debrief: Questions #3&4

## Slide 61
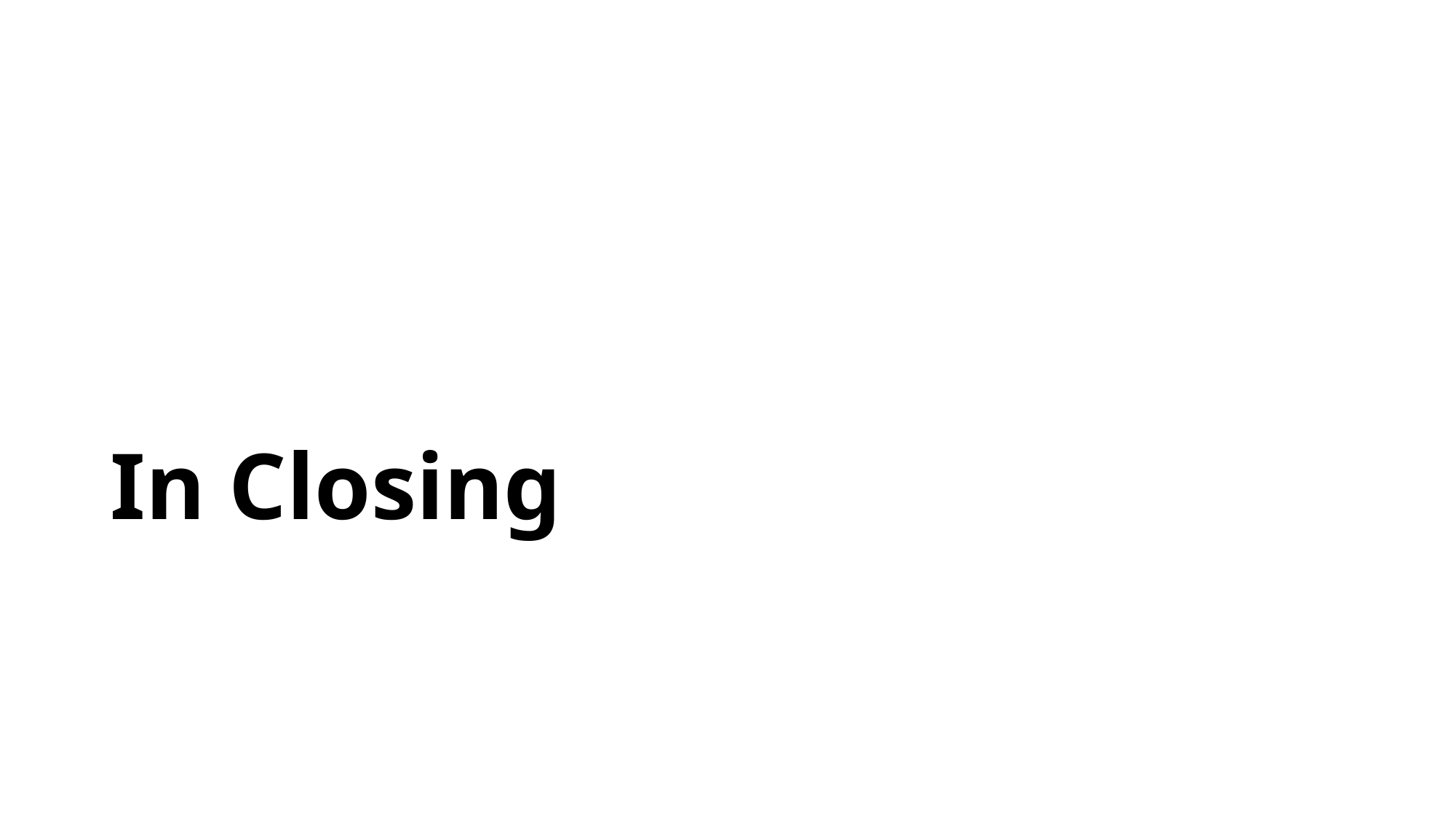

# In Closing

## Slide 62
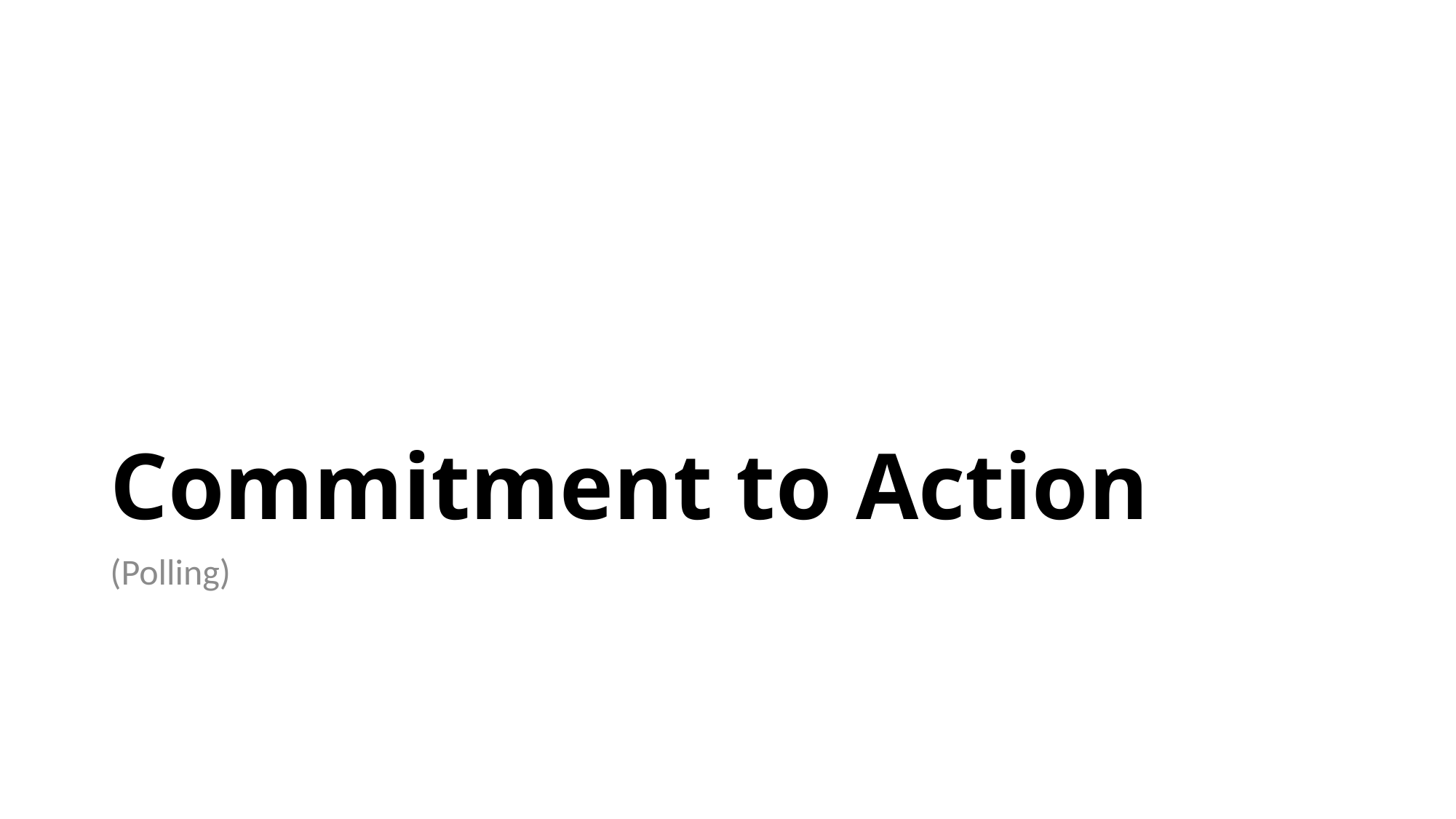

# Commitment to Action
(Polling)

## Slide 63
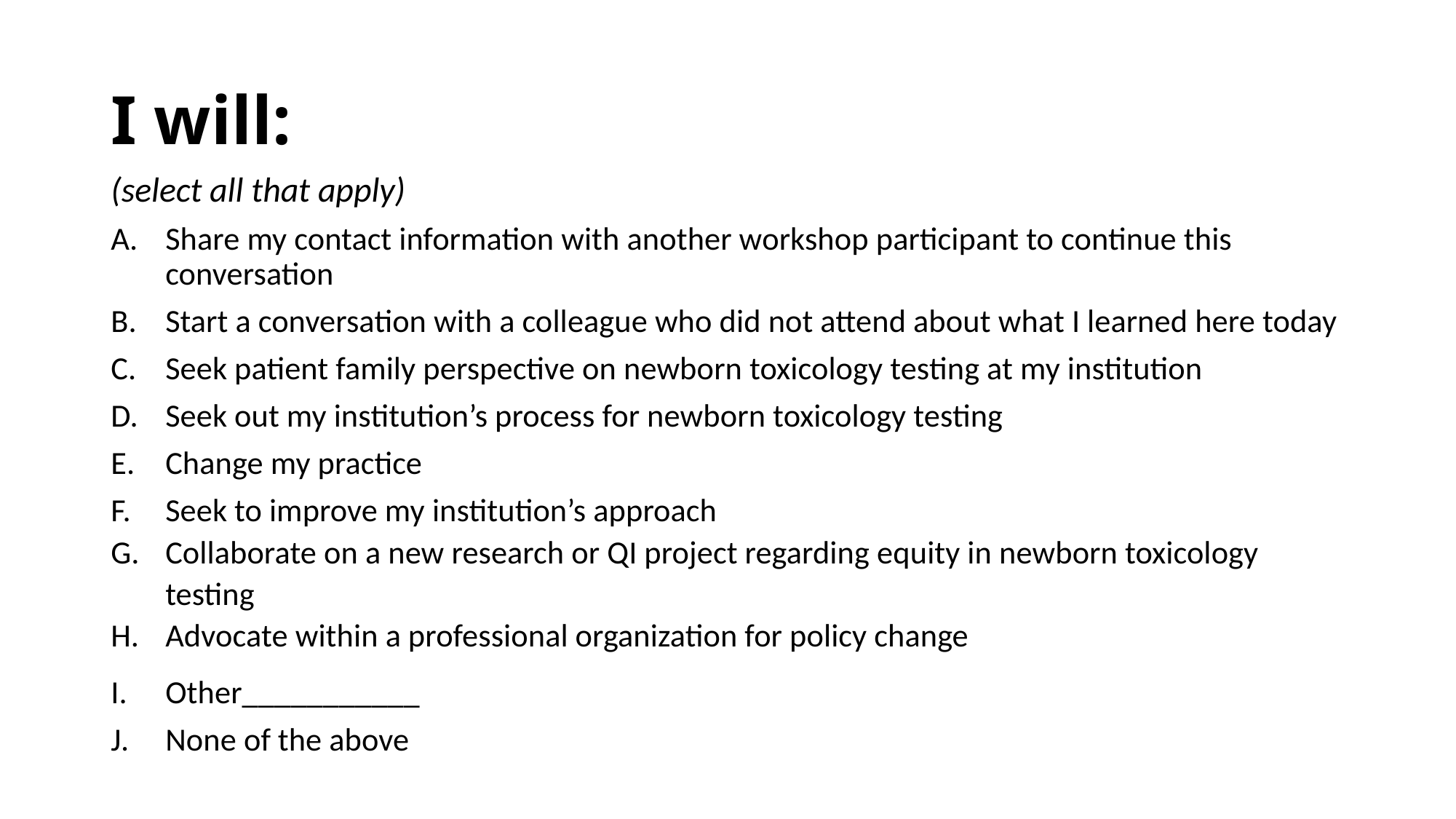

# I will:
(select all that apply)
Share my contact information with another workshop participant to continue this conversation
Start a conversation with a colleague who did not attend about what I learned here today
Seek patient family perspective on newborn toxicology testing at my institution
Seek out my institution’s process for newborn toxicology testing
Change my practice
Seek to improve my institution’s approach
Collaborate on a new research or QI project regarding equity in newborn toxicology testing
Advocate within a professional organization for policy change
Other___________
None of the above

## Slide 64
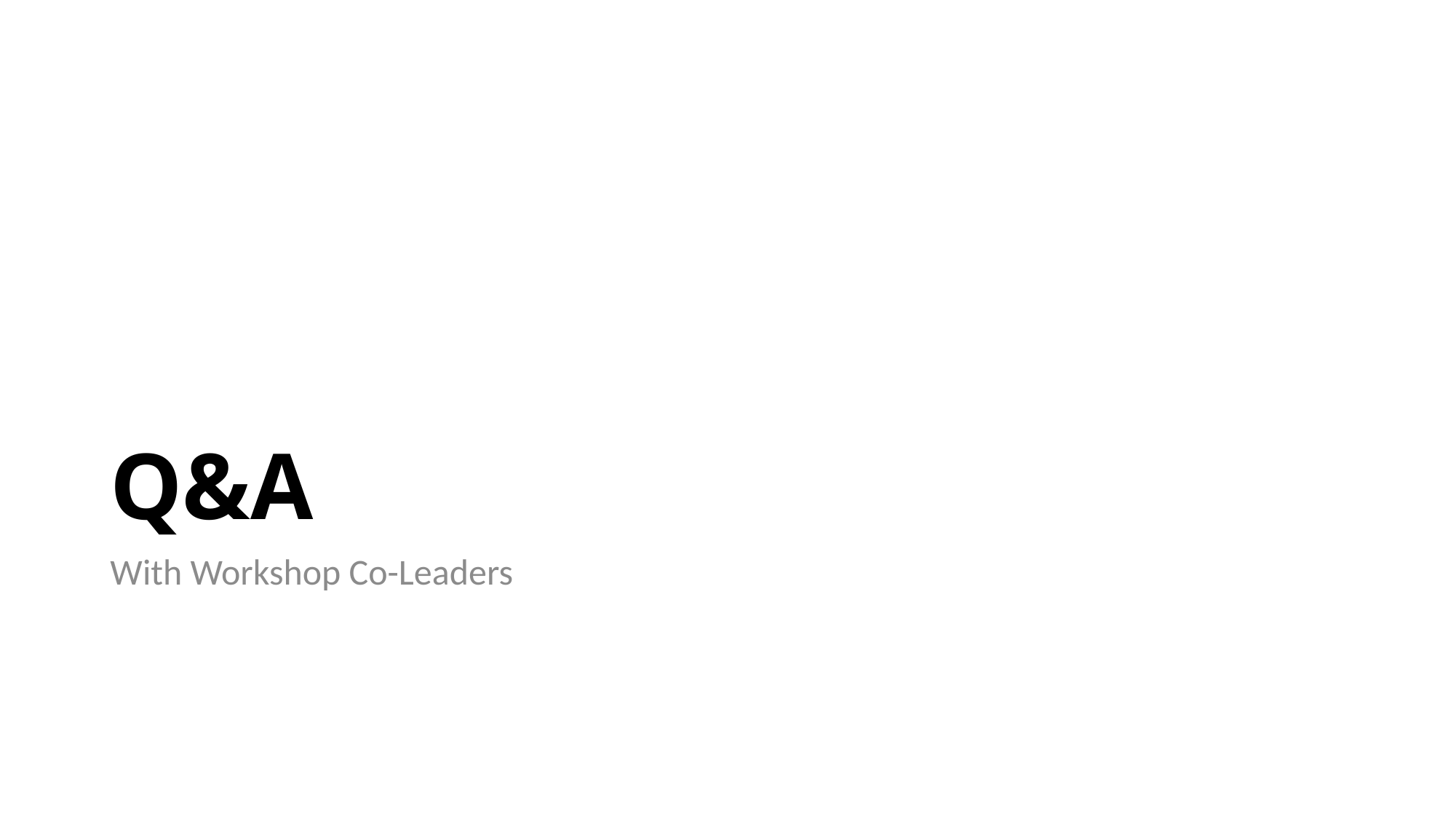

# Q&A
With Workshop Co-Leaders

## Slide 65
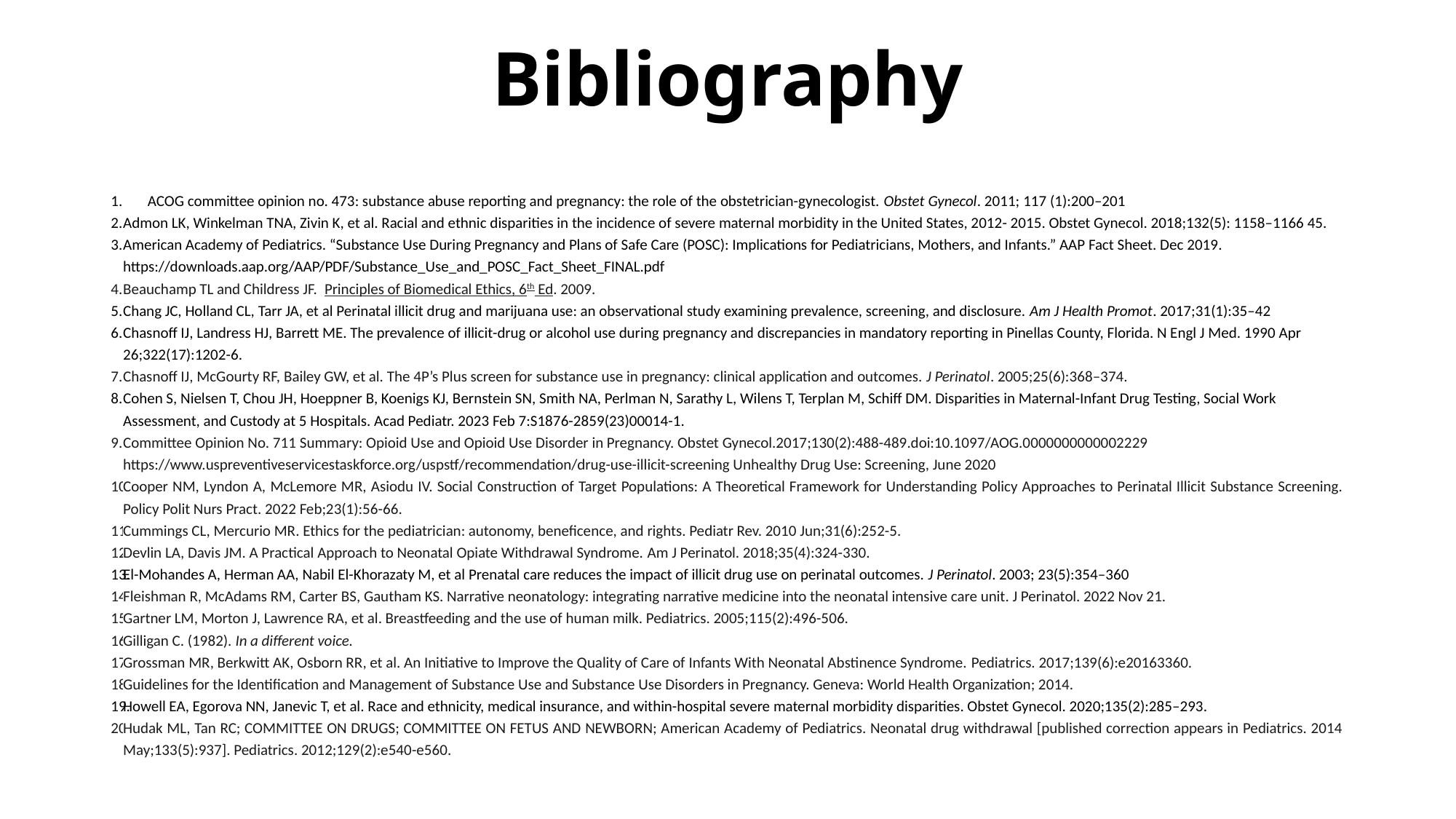

# Bibliography
ACOG committee opinion no. 473: substance abuse reporting and pregnancy: the role of the obstetrician-gynecologist. Obstet Gynecol. 2011; 117 (1):200–201
Admon LK, Winkelman TNA, Zivin K, et al. Racial and ethnic disparities in the incidence of severe maternal morbidity in the United States, 2012- 2015. Obstet Gynecol. 2018;132(5): 1158–1166 45.
American Academy of Pediatrics. “Substance Use During Pregnancy and Plans of Safe Care (POSC): Implications for Pediatricians, Mothers, and Infants.” AAP Fact Sheet. Dec 2019. https://downloads.aap.org/AAP/PDF/Substance_Use_and_POSC_Fact_Sheet_FINAL.pdf
Beauchamp TL and Childress JF. Principles of Biomedical Ethics, 6th Ed. 2009.
Chang JC, Holland CL, Tarr JA, et al Perinatal illicit drug and marijuana use: an observational study examining prevalence, screening, and disclosure. Am J Health Promot. 2017;31(1):35–42
Chasnoff IJ, Landress HJ, Barrett ME. The prevalence of illicit-drug or alcohol use during pregnancy and discrepancies in mandatory reporting in Pinellas County, Florida. N Engl J Med. 1990 Apr 26;322(17):1202-6.
Chasnoff IJ, McGourty RF, Bailey GW, et al. The 4P’s Plus screen for substance use in pregnancy: clinical application and outcomes. J Perinatol. 2005;25(6):368–374.
Cohen S, Nielsen T, Chou JH, Hoeppner B, Koenigs KJ, Bernstein SN, Smith NA, Perlman N, Sarathy L, Wilens T, Terplan M, Schiff DM. Disparities in Maternal-Infant Drug Testing, Social Work Assessment, and Custody at 5 Hospitals. Acad Pediatr. 2023 Feb 7:S1876-2859(23)00014-1.
Committee Opinion No. 711 Summary: Opioid Use and Opioid Use Disorder in Pregnancy. Obstet Gynecol.2017;130(2):488-489.doi:10.1097/AOG.0000000000002229 https://www.uspreventiveservicestaskforce.org/uspstf/recommendation/drug-use-illicit-screening Unhealthy Drug Use: Screening, June 2020
Cooper NM, Lyndon A, McLemore MR, Asiodu IV. Social Construction of Target Populations: A Theoretical Framework for Understanding Policy Approaches to Perinatal Illicit Substance Screening. Policy Polit Nurs Pract. 2022 Feb;23(1):56-66.
Cummings CL, Mercurio MR. Ethics for the pediatrician: autonomy, beneficence, and rights. Pediatr Rev. 2010 Jun;31(6):252-5.
Devlin LA, Davis JM. A Practical Approach to Neonatal Opiate Withdrawal Syndrome. Am J Perinatol. 2018;35(4):324-330.
El-Mohandes A, Herman AA, Nabil El-Khorazaty M, et al Prenatal care reduces the impact of illicit drug use on perinatal outcomes. J Perinatol. 2003; 23(5):354–360
Fleishman R, McAdams RM, Carter BS, Gautham KS. Narrative neonatology: integrating narrative medicine into the neonatal intensive care unit. J Perinatol. 2022 Nov 21.
Gartner LM, Morton J, Lawrence RA, et al. Breastfeeding and the use of human milk. Pediatrics. 2005;115(2):496-506.
Gilligan C. (1982). In a different voice.
Grossman MR, Berkwitt AK, Osborn RR, et al. An Initiative to Improve the Quality of Care of Infants With Neonatal Abstinence Syndrome. Pediatrics. 2017;139(6):e20163360.
Guidelines for the Identification and Management of Substance Use and Substance Use Disorders in Pregnancy. Geneva: World Health Organization; 2014.
Howell EA, Egorova NN, Janevic T, et al. Race and ethnicity, medical insurance, and within-hospital severe maternal morbidity disparities. Obstet Gynecol. 2020;135(2):285–293.
Hudak ML, Tan RC; COMMITTEE ON DRUGS; COMMITTEE ON FETUS AND NEWBORN; American Academy of Pediatrics. Neonatal drug withdrawal [published correction appears in Pediatrics. 2014 May;133(5):937]. Pediatrics. 2012;129(2):e540-e560.

## Slide 66
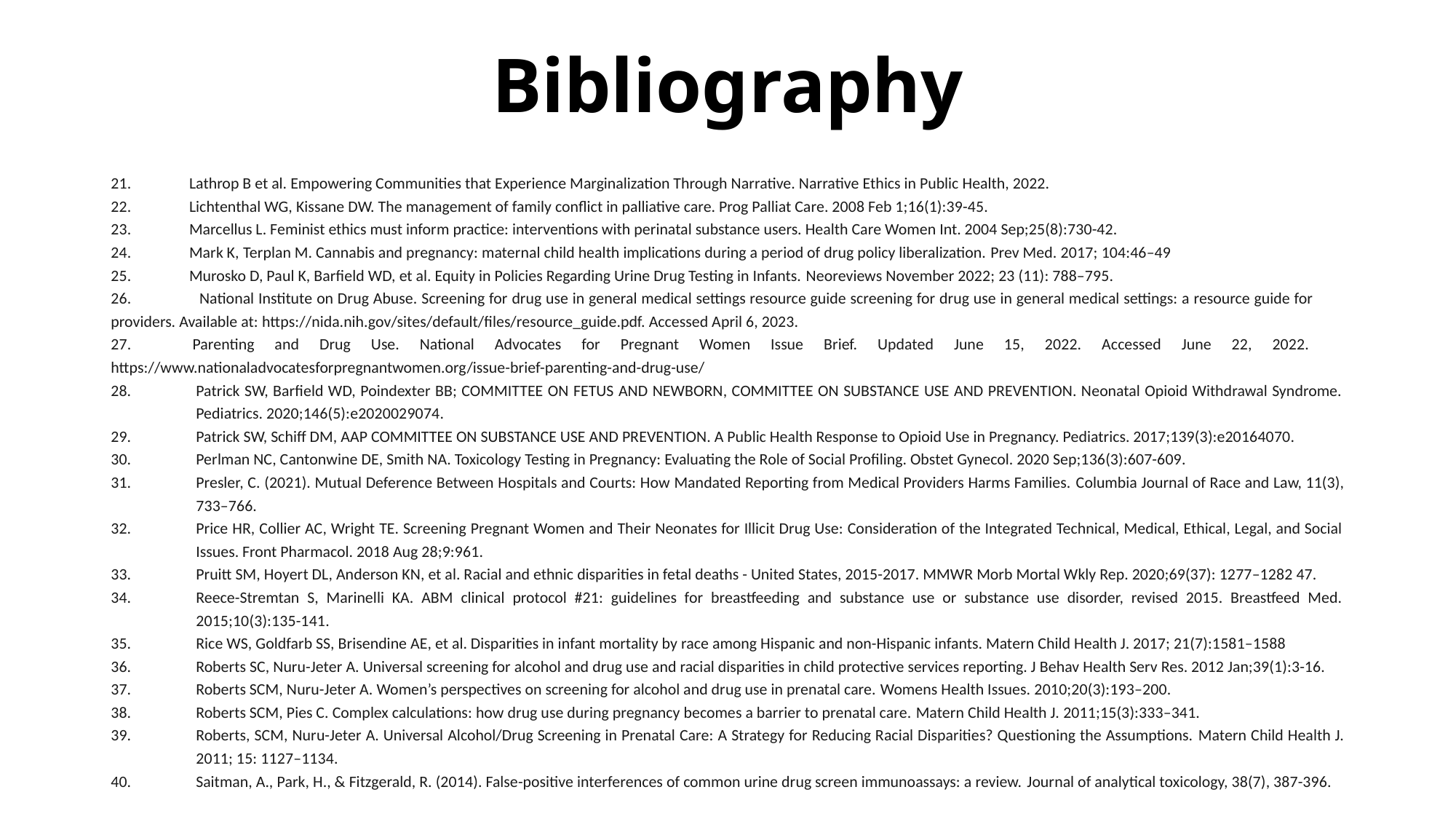

# Bibliography
21. Lathrop B et al. Empowering Communities that Experience Marginalization Through Narrative. Narrative Ethics in Public Health, 2022.
22. Lichtenthal WG, Kissane DW. The management of family conflict in palliative care. Prog Palliat Care. 2008 Feb 1;16(1):39-45.
23. Marcellus L. Feminist ethics must inform practice: interventions with perinatal substance users. Health Care Women Int. 2004 Sep;25(8):730-42.
24. Mark K, Terplan M. Cannabis and pregnancy: maternal child health implications during a period of drug policy liberalization. Prev Med. 2017; 104:46–49
25. Murosko D, Paul K, Barfield WD, et al. Equity in Policies Regarding Urine Drug Testing in Infants. Neoreviews November 2022; 23 (11): 788–795.
26. National Institute on Drug Abuse. Screening for drug use in general medical settings resource guide screening for drug use in general medical settings: a resource guide for 	providers. Available at: https://nida.nih.gov/sites/default/files/resource_guide.pdf. Accessed April 6, 2023.
27. Parenting and Drug Use. National Advocates for Pregnant Women Issue Brief. Updated June 15, 2022. Accessed June 22, 2022. 	https://www.nationaladvocatesforpregnantwomen.org/issue-brief-parenting-and-drug-use/
Patrick SW, Barfield WD, Poindexter BB; COMMITTEE ON FETUS AND NEWBORN, COMMITTEE ON SUBSTANCE USE AND PREVENTION. Neonatal Opioid Withdrawal Syndrome. Pediatrics. 2020;146(5):e2020029074.
Patrick SW, Schiff DM, AAP COMMITTEE ON SUBSTANCE USE AND PREVENTION. A Public Health Response to Opioid Use in Pregnancy. Pediatrics. 2017;139(3):e20164070.
Perlman NC, Cantonwine DE, Smith NA. Toxicology Testing in Pregnancy: Evaluating the Role of Social Profiling. Obstet Gynecol. 2020 Sep;136(3):607-609.
Presler, C. (2021). Mutual Deference Between Hospitals and Courts: How Mandated Reporting from Medical Providers Harms Families. Columbia Journal of Race and Law, 11(3), 733–766.
Price HR, Collier AC, Wright TE. Screening Pregnant Women and Their Neonates for Illicit Drug Use: Consideration of the Integrated Technical, Medical, Ethical, Legal, and Social Issues. Front Pharmacol. 2018 Aug 28;9:961.
Pruitt SM, Hoyert DL, Anderson KN, et al. Racial and ethnic disparities in fetal deaths - United States, 2015-2017. MMWR Morb Mortal Wkly Rep. 2020;69(37): 1277–1282 47.
Reece-Stremtan S, Marinelli KA. ABM clinical protocol #21: guidelines for breastfeeding and substance use or substance use disorder, revised 2015. Breastfeed Med. 2015;10(3):135-141.
Rice WS, Goldfarb SS, Brisendine AE, et al. Disparities in infant mortality by race among Hispanic and non-Hispanic infants. Matern Child Health J. 2017; 21(7):1581–1588
Roberts SC, Nuru-Jeter A. Universal screening for alcohol and drug use and racial disparities in child protective services reporting. J Behav Health Serv Res. 2012 Jan;39(1):3-16.
Roberts SCM, Nuru-Jeter A. Women’s perspectives on screening for alcohol and drug use in prenatal care. Womens Health Issues. 2010;20(3):193–200.
Roberts SCM, Pies C. Complex calculations: how drug use during pregnancy becomes a barrier to prenatal care. Matern Child Health J. 2011;15(3):333–341.
Roberts, SCM, Nuru-Jeter A. Universal Alcohol/Drug Screening in Prenatal Care: A Strategy for Reducing Racial Disparities? Questioning the Assumptions. Matern Child Health J. 2011; 15: 1127–1134.
Saitman, A., Park, H., & Fitzgerald, R. (2014). False-positive interferences of common urine drug screen immunoassays: a review. Journal of analytical toxicology, 38(7), 387-396.

## Slide 67
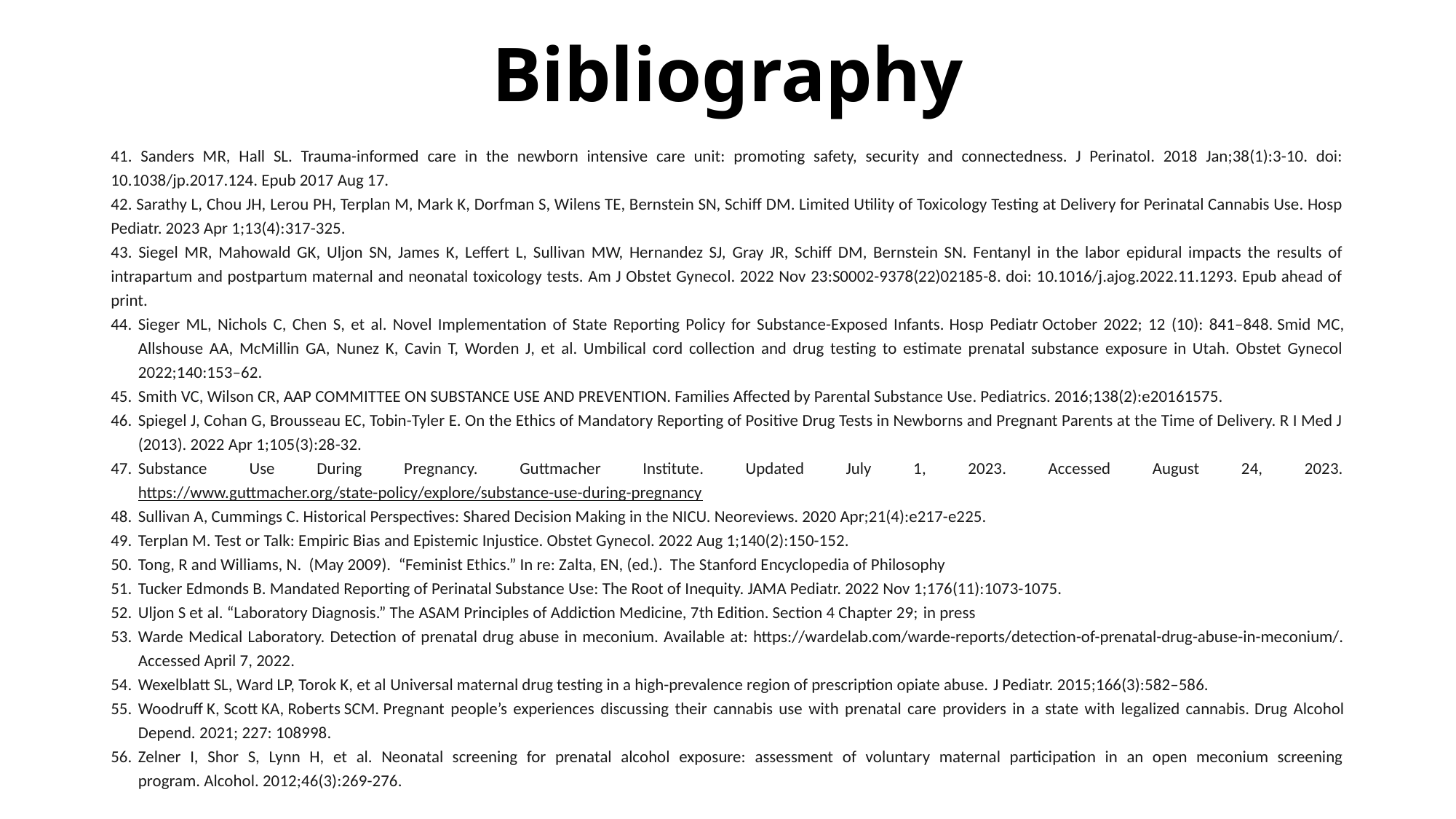

# Bibliography
41. Sanders MR, Hall SL. Trauma-informed care in the newborn intensive care unit: promoting safety, security and connectedness. J Perinatol. 2018 Jan;38(1):3-10. doi: 10.1038/jp.2017.124. Epub 2017 Aug 17.
42. Sarathy L, Chou JH, Lerou PH, Terplan M, Mark K, Dorfman S, Wilens TE, Bernstein SN, Schiff DM. Limited Utility of Toxicology Testing at Delivery for Perinatal Cannabis Use. Hosp Pediatr. 2023 Apr 1;13(4):317-325.
43. Siegel MR, Mahowald GK, Uljon SN, James K, Leffert L, Sullivan MW, Hernandez SJ, Gray JR, Schiff DM, Bernstein SN. Fentanyl in the labor epidural impacts the results of intrapartum and postpartum maternal and neonatal toxicology tests. Am J Obstet Gynecol. 2022 Nov 23:S0002-9378(22)02185-8. doi: 10.1016/j.ajog.2022.11.1293. Epub ahead of print.
Sieger ML, Nichols C, Chen S, et al. Novel Implementation of State Reporting Policy for Substance-Exposed Infants. Hosp Pediatr October 2022; 12 (10): 841–848. Smid MC, Allshouse AA, McMillin GA, Nunez K, Cavin T, Worden J, et al. Umbilical cord collection and drug testing to estimate prenatal substance exposure in Utah. Obstet Gynecol 2022;140:153–62.
Smith VC, Wilson CR, AAP COMMITTEE ON SUBSTANCE USE AND PREVENTION. Families Affected by Parental Substance Use. Pediatrics. 2016;138(2):e20161575.
Spiegel J, Cohan G, Brousseau EC, Tobin-Tyler E. On the Ethics of Mandatory Reporting of Positive Drug Tests in Newborns and Pregnant Parents at the Time of Delivery. R I Med J (2013). 2022 Apr 1;105(3):28-32.
Substance Use During Pregnancy. Guttmacher Institute. Updated July 1, 2023. Accessed August 24, 2023. https://www.guttmacher.org/state-policy/explore/substance-use-during-pregnancy
Sullivan A, Cummings C. Historical Perspectives: Shared Decision Making in the NICU. Neoreviews. 2020 Apr;21(4):e217-e225.
Terplan M. Test or Talk: Empiric Bias and Epistemic Injustice. Obstet Gynecol. 2022 Aug 1;140(2):150-152.
Tong, R and Williams, N. (May 2009). “Feminist Ethics.” In re: Zalta, EN, (ed.). The Stanford Encyclopedia of Philosophy
Tucker Edmonds B. Mandated Reporting of Perinatal Substance Use: The Root of Inequity. JAMA Pediatr. 2022 Nov 1;176(11):1073-1075.
Uljon S et al. “Laboratory Diagnosis.” The ASAM Principles of Addiction Medicine, 7th Edition. Section 4 Chapter 29; in press
Warde Medical Laboratory. Detection of prenatal drug abuse in meconium. Available at: https://wardelab.com/warde-reports/detection-of-prenatal-drug-abuse-in-meconium/. Accessed April 7, 2022.
Wexelblatt SL, Ward LP, Torok K, et al Universal maternal drug testing in a high-prevalence region of prescription opiate abuse. J Pediatr. 2015;166(3):582–586.
Woodruff K, Scott KA, Roberts SCM. Pregnant people’s experiences discussing their cannabis use with prenatal care providers in a state with legalized cannabis. Drug Alcohol Depend. 2021; 227: 108998.
Zelner I, Shor S, Lynn H, et al. Neonatal screening for prenatal alcohol exposure: assessment of voluntary maternal participation in an open meconium screening program. Alcohol. 2012;46(3):269-276.
