# Supplementary material for: How Can Newborn Toxicology Testing Be More Equitable? An Interactive Ethics Workshop
Source: MedEdPORTAL. 2024 Sep 10;20:11434. doi: 10.15766/mep_2374-8265.11434 (PMC11383834; doi:10.15766/mep_2374-8265.11434)
Supplement: Supplementary file 1 — Newborn Toxicology Workshop Slides.pptxParticipant Workbook.docxFacilitator Guide.docxSurvey 1.docxSurvey 2.docx [file mep_2374-8265.11434-s001.zip › B. Participant Workbook.docx]

**“How Can Newborn Toxicology Testing be More Equitable?”**

**Participant Workbook**

**Table of Contents**

Page 3 Objectives and Instructions

Page 4 Case #1

Page 5 Case #2

Page 6 Case #3

Page 7 Case #4

Page 8 Bibliography

**Objectives**

Through alternating facilitated small group discussion and “mini didactics” from content experts, participants will:

1. Assess the potential benefits and harms of current and alternative approaches to newborn toxicology testing.

1. Analyze varied approaches to newborn toxicology testing with regard to informed consent, bias and justice.

1. Leverage ethical analysis to reflect critically upon one’s own practice environment and identify actionable areas for change and/or advocacy.

**Order of Activities**

Session Leaders will introduce the workshop.

We will get to know each other through large group polling and small group introductions.

Each participant will be part of a small group with workshop co-leader(s). Each small group is assigned a particular patient case to discuss. Small groups will move through four discussion questions regarding their assigned case during the workshop.

**My case number is _____.**

Case 1 is found on Page 4 of this workbook. Case 2 is on Page 5. Case 3 is on Page 6. Case 4 is on Page 7.

For your reference, a bibliography of relevant sources can be found on pages 8-11 of this workbook.

**Case #1**

*Cases represent amalgamations of real-world patient care scenarios and do not necessarily reflect individual patient narratives.*

Armani was delivered at term after an unremarkable pregnancy. Her mother, Jada, had normal prenatal care including ultrasounds and lab work. During her second day of life, Armani develops jitteriness and hypertonia. She is hypoglycemic but her symptoms do not resolve with dextrose gel and normalization of her blood glucose. She is transferred to the neonatal intensive care unit for further evaluation and treatment. You review the pregnancy history with Jada, and she denies opioid or other substance use during pregnancy. Given the severity of Armani’s neurologic symptoms, you are considering performing a lumbar puncture, ordering an EEG and brain MRI.

**Small Group Discussion Questions**

*Each small group is assigned one of four cases.*

*All small groups will move through the following four discussion questions over the course of the workshop, discussing in relation to their assigned case.*

1. Are there indications to obtain toxicology testing from Armani? In your opinion, which indications are valuable or high yield?
2. What are the risks or limitations to toxicology testing for Armani? How do you balance the potential benefits and harms?
3. What are additional ethical considerations regarding toxicology testing for Armani?
4. How would you discuss newborn toxicology testing with Jada, Armani’s parent?

**Case #2**

*Cases represent amalgamations of real-world patient care scenarios and do not necessarily reflect individual patient narratives.*

Olivia was delivered at term. Her mother Emma’s pregnancy was notable for a paucity of prenatal care – she only received a dating ultrasound. This ultrasound was performed during a hospitalization for substance withdrawal while Emma was in police custody. She reported using heroin and cocaine during her pregnancy. Emma’s urine toxicology testing at time of delivery was positive for heroin, fentanyl, and cocaine. Olivia was admitted to the NICU with respiratory distress.

**Small Group Discussion Questions**

*Each small group is assigned one of four cases.*

*All small groups will move through the following four discussion questions over the course of the workshop, discussing in relation to their assigned case.*

1. Are there indications to obtain toxicology testing from Olivia? In your opinion, which indications are valuable or high yield?
2. What are the risks or limitations to toxicology testing for Olivia? How do you balance the potential benefits and harms?
3. What are additional ethical considerations regarding toxicology testing for Olivia?
4. How would you discuss newborn toxicology testing with Emma, Olivia’s parent?

**Case #3**

*Cases represent amalgamations of real-world patient care scenarios and do not necessarily reflect individual patient narratives.*

Clara is a term newborn delivered vaginally after an unremarkable pregnancy. Throughout the pregnancy, her mother, Gabriela, told her OB that she was using cannabis to improve her appetite and help her sleep. Maternal urine toxicology was positive for cannabis throughout pregnancy and at delivery. You consult social work who reports that there is no prior Child Protective Services (CPS) involvement for Gabriela’s two, older children.

**Small Group Discussion Questions**

*Each small group is assigned one of four cases.*

*All small groups will move through the following four discussion questions over the course of the workshop, discussing in relation to their assigned case.*

1. Are there indications to obtain toxicology testing from Clara? In your opinion, which indications are valuable or high yield?
2. What are the risks or limitations to toxicology testing for Clara? How do you balance the potential benefits and harms?
3. What are additional ethical considerations regarding toxicology testing for Clara?
4. How would you discuss newborn toxicology testing with Gabriela, Clara’s parent?

**Case #4**

*Cases represent amalgamations of real-world patient care scenarios and do not necessarily reflect individual patient narratives.*

Li is delivered at term after a pregnancy notable for an absence of prenatal care. Li’s mother, Xiang (pronounced SHYAHNG), reports that she unsuccessfully attempted to make appointments. Given this history, your colleagues order urine toxicology testing on Xiang at time of delivery. Her test is positive for cocaine, and she states that it is a “mystery” to her how this could be possible. Social work reports this to Child Protective Services (CPS) and the CPS supervisor requests testing on Li.

**Small Group Discussion Questions**

*Each small group is assigned one of four cases.*

*All small groups will move through the following four discussion questions over the course of the workshop, discussing in relation to their assigned case.*

1. Are there indications to obtain toxicology testing from Li? In your opinion, which indications are valuable or high yield?
2. What are the risks or limitations to toxicology testing for Li? How do you balance the potential benefits and harms?
3. What are additional ethical considerations regarding toxicology testing for Li?
4. How would you discuss newborn toxicology testing with Xiang, Li’s parent?

**Bibliography**

ACOG committee opinion no. 473: substance abuse reporting and pregnancy: the role of the obstetrician-gynecologist. *Obstet Gynecol*. 2011; 117 (1):200–201

Admon LK, Winkelman TNA, Zivin K, et al. Racial and ethnic disparities in the incidence of severe maternal morbidity in the United States, 2012- 2015. Obstet Gynecol. 2018;132(5): 1158–1166 45.

American Academy of Pediatrics. “Substance Use During Pregnancy and Plans of Safe Care (POSC): Implications for Pediatricians, Mothers, and Infants.” AAP Fact Sheet. Dec 2019. https://downloads.aap.org/AAP/PDF/Substance_Use_and_POSC_Fact_Sheet_FINAL.pdf

Beauchamp TL and Childress JF. Principles of Biomedical Ethics, 6^th^ Ed. 2009.

Chang JC, Holland CL, Tarr JA, et al Perinatal illicit drug and marijuana use: an observational study examining prevalence, screening, and disclosure. *Am J Health Promot*. 2017;31(1):35–42

Chasnoff IJ, Landress HJ, Barrett ME. The prevalence of illicit-drug or alcohol use during pregnancy and discrepancies in mandatory reporting in Pinellas County, Florida. N Engl J Med. 1990 Apr 26;322(17):1202-6. doi: 10.1056/NEJM199004263221706. PMID: 2325711.

Chasnoff IJ, McGourty RF, Bailey GW, et al. The 4P’s Plus screen for substance use in pregnancy: clinical application and outcomes. *J Perinatol*. 2005;25(6):368–374.

Cohen S, Nielsen T, Chou JH, Hoeppner B, Koenigs KJ, Bernstein SN, Smith NA, Perlman N, Sarathy L, Wilens T, Terplan M, Schiff DM. Disparities in Maternal-Infant Drug Testing, Social Work Assessment, and Custody at 5 Hospitals. Acad Pediatr. 2023 Feb 7:S1876-2859(23)00014-1. doi: 10.1016/j.acap.2023.01.012. Epub ahead of print. PMID: 36754165.

Committee Opinion No. 711 Summary: Opioid Use and Opioid Use Disorder in Pregnancy. Obstet Gynecol.2017;130(2):488-489.doi:10.1097/AOG.0000000000002229 <https://www.uspreventiveservicestaskforce.org/uspstf/recommendation/drug-use-illicit-screening> Unhealthy Drug Use: Screening, June 2020

Cooper NM, Lyndon A, McLemore MR, Asiodu IV. Social Construction of Target Populations: A Theoretical Framework for Understanding Policy Approaches to Perinatal Illicit Substance Screening. Policy Polit Nurs Pract. 2022 Feb;23(1):56-66. doi: 10.1177/15271544211067781.

Cummings CL, Mercurio MR. Ethics for the pediatrician: autonomy, beneficence, and rights. Pediatr Rev. 2010 Jun;31(6):252-5. doi: 10.1542/pir.31-6-252. PMID: 20516238.

Devlin LA, Davis JM. A Practical Approach to Neonatal Opiate Withdrawal Syndrome. Am J Perinatol. 2018;35(4):324-330. doi:10.1055/s-0037-1608630

El-Mohandes A, Herman AA, Nabil El-Khorazaty M, et al Prenatal care reduces the impact of illicit drug use on perinatal outcomes. *J Perinatol*. 2003; 23(5):354–360

Fleishman R, McAdams RM, Carter BS, Gautham KS. Narrative neonatology: integrating narrative medicine into the neonatal intensive care unit. J Perinatol. 2022 Nov 21. doi: 10.1038/s41372-022-01565-5. Epub ahead of print. PMID: 36414736.

Gartner LM, Morton J, Lawrence RA, et al. Breastfeeding and the use of human milk. Pediatrics. 2005;115(2):496-506. doi:10.1542/peds.2004-2491

Gilligan C. (1982). *In a different voice.*

Grossman MR, Berkwitt AK, Osborn RR, et al. An Initiative to Improve the Quality of Care of Infants With Neonatal Abstinence Syndrome. Pediatrics. 2017;139(6):e20163360. doi:10.1542/peds.2016-3360

Guidelines for the Identification and Management of Substance Use and Substance Use Disorders in Pregnancy. Geneva: World Health Organization; 2014.

Howell EA, Egorova NN, Janevic T, et al. Race and ethnicity, medical insurance, and within-hospital severe maternal morbidity disparities. Obstet Gynecol. 2020;135(2):285–293 46.

Hudak ML, Tan RC; COMMITTEE ON DRUGS; COMMITTEE ON FETUS AND NEWBORN; American Academy of Pediatrics. Neonatal drug withdrawal [published correction appears in Pediatrics. 2014 May;133(5):937]. Pediatrics. 2012;129(2):e540-e560. doi:10.1542/peds.2011-3212

Lathrop B et al. Empowering Communities that Experience Marginalization Through Narrative. Narrative Ethics in Public Health, 2022.

Lichtenthal WG, Kissane DW. The management of family conflict in palliative care. Prog Palliat Care. 2008 Feb 1;16(1):39-45. doi: 10.1179/096992608x296914.

Marcellus L. Feminist ethics must inform practice: interventions with perinatal substance users. Health Care Women Int. 2004 Sep;25(8):730-42. doi: 10.1080/07399330490475584.

Mark K, Terplan M. Cannabis and pregnancy: maternal child health implications during a period of drug policy liberalization. *Prev Med*. 2017; 104:46–49

Murosko D, Paul K, Barfield WD, et al. Equity in Policies Regarding Urine Drug Testing in Infants. *Neoreviews* November 2022; 23 (11): 788–795. <https://doi.org/10.1542/neo.23-10-e788>

National Institute on Drug Abuse. Screening for drug use in general medical settings resource guide screening for drug use in general medical settings: a resource guide for providers. Available at: https://nida.nih.gov/sites/default/files/resource_guide.pdf. Accessed April 6, 2023.

Parenting and Drug Use. National Advocates for Pregnant Women Issue Brief. Updated June 15, 2022. Accessed June 22, 2022. https://www.nationaladvocatesforpregnantwomen.org/issue-brief-parenting-and-drug-use/

Patrick SW, Barfield WD, Poindexter BB; COMMITTEE ON FETUS AND NEWBORN, COMMITTEE ON SUBSTANCE USE AND PREVENTION. Neonatal Opioid Withdrawal Syndrome. Pediatrics. 2020;146(5):e2020029074. doi:10.1542/peds.2020-029074

Patrick SW, Schiff DM, AAP COMMITTEE ON SUBSTANCE USE AND PREVENTION. A Public Health Response to Opioid Use

in Pregnancy. Pediatrics. 2017;139(3):e20164070. <https://doi.org/10.1542/peds.2016-4070>

Perlman NC, Cantonwine DE, Smith NA. Toxicology Testing in Pregnancy: Evaluating the Role of Social Profiling. Obstet Gynecol. 2020 Sep;136(3):607-609. doi: 10.1097/AOG.0000000000003986. PMID: 32769654.

Presler, C. (2021). Mutual Deference Between Hospitals and Courts: How Mandated Reporting from Medical Providers Harms Families. Columbia Journal of Race and Law, 11(3), 733–766. https://doi.org/10.52214/cjrl.v11i3.8750

Price HR, Collier AC, Wright TE. Screening Pregnant Women and Their Neonates for Illicit Drug Use: Consideration of the Integrated Technical, Medical, Ethical, Legal, and Social Issues. Front Pharmacol. 2018 Aug 28;9:961. doi: 10.3389/fphar.2018.00961.

Pruitt SM, Hoyert DL, Anderson KN, et al. Racial and ethnic disparities in fetal deaths - United States, 2015-2017. MMWR Morb Mortal Wkly Rep. 2020;69(37): 1277–1282 47.

Reece-Stremtan S, Marinelli KA. ABM clinical protocol #21: guidelines for breastfeeding and substance use or substance use disorder, revised 2015. Breastfeed Med. 2015;10(3):135-141. doi:10.1089/bfm.2015.9992

Rice WS, Goldfarb SS, Brisendine AE, et al. Disparities in infant mortality by race among Hispanic and non-Hispanic infants. Matern Child Health J. 2017; 21(7):1581–1588

Roberts SC, Nuru-Jeter A. Universal screening for alcohol and drug use and racial disparities in child protective services reporting. J Behav Health Serv Res. 2012 Jan;39(1):3-16. doi: 10.1007/s11414-011-9247-x. PMID: 21681593; PMCID: PMC3297420.

Roberts SCM, Nuru-Jeter A. Women’s perspectives on screening for alcohol and drug use in prenatal care. *Womens Health Issues*. 2010;20(3):193–200

Roberts SCM, Pies C. Complex calculations: how drug use during pregnancy becomes a barrier to prenatal care. *Matern Child Health J*. 2011;15(3):333–341

Roberts, SCM, Nuru-Jeter A. Universal Alcohol/Drug Screening in Prenatal Care: A Strategy for Reducing Racial Disparities? Questioning the Assumptions. *Matern Child Health J.* 2011; 15: 1127–1134.

Saitman, A., Park, H., & Fitzgerald, R. (2014). False-positive interferences of common urine drug screen immunoassays: a review. *Journal of analytical toxicology*, 38(7), 387-396.

Sanders MR, Hall SL. Trauma-informed care in the newborn intensive care unit: promoting safety, security and connectedness. J Perinatol. 2018 Jan;38(1):3-10. doi: 10.1038/jp.2017.124. Epub 2017 Aug 17.

Sarathy L, Chou JH, Lerou PH, Terplan M, Mark K, Dorfman S, Wilens TE, Bernstein SN, Schiff DM. Limited Utility of Toxicology Testing at Delivery for Perinatal Cannabis Use. Hosp Pediatr. 2023 Apr 1;13(4):317-325. doi: 10.1542/hpeds.2022-006897. PMID: 36855896; PMCID: PMC10071432.

Siegel MR, Mahowald GK, Uljon SN, James K, Leffert L, Sullivan MW, Hernandez SJ, Gray JR, Schiff DM, Bernstein SN. Fentanyl in the labor epidural impacts the results of intrapartum and postpartum maternal and neonatal toxicology tests. Am J Obstet Gynecol. 2022 Nov 23:S0002-9378(22)02185-8. doi: 10.1016/j.ajog.2022.11.1293. Epub ahead of print. PMID: 36427599.

Sieger ML, Nichols C, Chen S, et al. Novel Implementation of State Reporting Policy for Substance-Exposed Infants. *Hosp Pediatr* October 2022; 12 (10): 841–848. <https://doi.org/10.1542/hpeds.2022-006562>

Smid MC, Allshouse AA, McMillin GA, Nunez K, Cavin T, Worden J, et al. Umbilical cord collection and drug testing to estimate prenatal substance exposure in Utah. Obstet Gynecol 2022;140:153–62. doi: 10.1097/AOG.0000000000004868

Smith VC, Wilson CR, AAP COMMITTEE ON SUBSTANCE USE AND PREVENTION. Families Affected by Parental Substance

Use. Pediatrics. 2016;138(2):e20161575.  DOI: [10.1542/peds.2016-1575](https://doi.org/10.1542/peds.2016-1575)

Spiegel J, Cohan G, Brousseau EC, Tobin-Tyler E. On the Ethics of Mandatory Reporting of Positive Drug Tests in Newborns and Pregnant Parents at the Time of Delivery. R I Med J (2013). 2022 Apr 1;105(3):28-32. PMID: 35349617.

Substance Use During Pregnancy. Guttmacher Institute. Updated July 1, 2023. Accessed August 24, 2023. <https://www.guttmacher.org/state-policy/explore/substance-use-during-pregnancy>

Sullivan A, Cummings C. Historical Perspectives: Shared Decision Making in the NICU. Neoreviews. 2020 Apr;21(4):e217-e225. doi: 10.1542/neo.21-4-e217.

Terplan M. Test or Talk: Empiric Bias and Epistemic Injustice. Obstet Gynecol. 2022 Aug 1;140(2):150-152.

Tong, R and Williams, N. (May 2009). “Feminist Ethics.” In re: Zalta, EN, (ed.). *The Stanford Encyclopedia of Philosophy*

Tucker Edmonds B. Mandated Reporting of Perinatal Substance Use: The Root of Inequity. JAMA Pediatr. 2022 Nov 1;176(11):1073-1075. doi: 10.1001/jamapediatrics.2022.3404.

Uljon S et al. “Laboratory Diagnosis.” *The ASAM Principles of Addiction Medicine, 7^th^ Edition.* Section 4 Chapter 29; *in press*

Warde Medical Laboratory. Detection of prenatal drug abuse in meconium. Available at: https://wardelab.com/warde-reports/detection-of-prenatal-drug-abuse-in-meconium/. Accessed April 7, 2022

Wexelblatt SL, Ward LP, Torok K, et al Universal maternal drug testing in a high-prevalence region of prescription opiate abuse. *J Pediatr*. 2015;166(3):582–586

Woodruff K, Scott KA, Roberts SCM. Pregnant people’s experiences discussing their cannabis use with prenatal care providers in a state with legalized cannabis. *Drug Alcohol Depend*. 2021; 227: 108998.

Zelner I, Shor S, Lynn H, et al. Neonatal screening for prenatal alcohol exposure: assessment of voluntary maternal participation in an open meconium screening program. Alcohol. 2012;46(3):269-276. doi:10.1016/j.alcohol.2011.09.029
