# Supplementary material for: How Can Newborn Toxicology Testing Be More Equitable? An Interactive Ethics Workshop
Source: MedEdPORTAL. 2024 Sep 10;20:11434. doi: 10.15766/mep_2374-8265.11434 (PMC11383834; doi:10.15766/mep_2374-8265.11434)
Supplement: Supplementary file 1 — Newborn Toxicology Workshop Slides.pptxParticipant Workbook.docxFacilitator Guide.docxSurvey 1.docxSurvey 2.docx [file mep_2374-8265.11434-s001.zip › D. Survey 1.docx]

**Survey 1 – Immediate Post-workshop**

1. Informed Consent: Thank you for participating in our Workshop "How Can Newborn Toxicology Testing be More Equitable?". We are conducting a voluntary research survey project to understand how this workshop may influence the perspective and/or behavior of workshop attendees over time. Although you will not get personal benefit from taking part in this survey research project, your responses may help us understand more about the effectiveness of this workshop and ways to improve it in the future. We hope to receive completed surveys from the majority of workshop attendees, so your answers are important to us. You can decide whether to take part in this research or not. You are free to say yes or no. Even if you join this project, you do not have to stay in it. If you do participate, you are free to skip any questions or discontinue at any time. The workshop leadership team will not know that any information you provided came from you, nor even whether you participated in the study. Free text survey responses may be quoted in future presentations and/or publications describing this workshop. Should responses be quoted, any identifying details will be removed. The survey will ask you to share an email address where you can receive a second survey invitation in three months time. This email address will be kept confidential and not linked to the other survey responses you choose to share. You will then receive an email invitation to answer some questions about your opinions and habits in three months time. Each survey should take you around five minutes to complete. You will not receive anything for your participation. Your participation in this research is entirely voluntary. If you have questions about the study, please feel free to ask; the contact information for the study leader is: ___________________________
   1. I consent, begin the study
   2. I do not consent, I do not wish to participate
2. Are you a trainee?
   1. No
   2. Yes, I’m a professional student (but not studying to be a physician)
   3. Yes, I’m a medical student
   4. Yes, I’m a resident
   5. Yes, I’m a fellow
3. What is your training background? Select all completed training programs that apply.
   1. General Pediatrics Residency
   2. Medicine-Pediatrics Residency
   3. Pediatric Hospital Medicine Fellowship
   4. Family Medicine Residency
   5. Obstetrics and Gynecology Residency
   6. Maternal Fetal Medicine Residency
   7. Neonatology Fellowship
   8. Child Abuse Pediatrics Fellowship
   9. Bioethicist
   10. Advanced Practice Provider
   11. Nursing
   12. Lawyer
   13. Health Policy
   14. Healthcare Administration
   15. Other________
4. Do you work in a practice setting where you might order toxicology testing (urine, meconium, cord, etc) on a newborn during the birth hospitalization?
   1. Yes
   2. No
   3. Other (Please specify) ______________________________________________
5. How likely are you to recommend this workshop to a friend or colleague?
   1. 0 – not at all likely
   2. 1
   3. 2
   4. 3
   5. 4
   6. 5
   7. 6
   8. 7
   9. 8
   10. 9
   11. 10 – extremely likely
6. Did participating in this workshop introduce you to a new idea or a new way to discuss newborn toxicology testing?
   1. Yes
      1. If yes, please describe ______________________________________________
   2. No
7. Are you considering a change in the way you or your institution practices newborn toxicology testing after attending this workshop?
   1. Yes
      1. If yes, please describe ______________________________________________
   2. No
8. We want to check in with you later! Please share an email where you consent to receive an electronic survey invitation in three months time.

___________________________________________

1. In your opinion, what were the strengths of this workshop?

___________________________________________

1. In your opinion, how could this workshop be improved?

___________________________________________
