# Supplementary material for: How Can Newborn Toxicology Testing Be More Equitable? An Interactive Ethics Workshop
Source: MedEdPORTAL. 2024 Sep 10;20:11434. doi: 10.15766/mep_2374-8265.11434 (PMC11383834; doi:10.15766/mep_2374-8265.11434)
Supplement: Supplementary file 1 — Newborn Toxicology Workshop Slides.pptxParticipant Workbook.docxFacilitator Guide.docxSurvey 1.docxSurvey 2.docx [file mep_2374-8265.11434-s001.zip › E. Survey 2.docx]

**Survey 2 – 3-Month Post-Workshop**

1. Let’s make sure we reached the correct person! Did you attend the workshop entitled “How Can Newborn Toxicology Testing be Equitable?”
   1. Yes
   2. No
2. Informed Consent: Thank you for participating in our Workshop "How Can Newborn Toxicology Testing be More Equitable?" three months ago. We are conducting a voluntary research survey project to understand how this workshop may influence the perspective and/or behavior of workshop attendees over time. Although you will not get personal benefit from taking part in this survey research project, your responses may help us understand more about the effectiveness of this workshop and ways to improve it in the future. We hope to receive completed surveys from the majority of workshop attendees, so your answers are important to us. You can decide whether to take part in this research or not. You are free to say yes or no. Even if you join this project, you do not have to stay in it. If you do participate, you are free to skip any questions or discontinue at any time. The workshop leadership team will not know that any information you provided came from you, nor even whether you participated in the study. Free text survey responses may be quoted in future presentations and/or publications describing this workshop. Should responses be quoted, any identifying details will be removed. This survey should take you about five minutes to complete. You will not receive anything for your participation. Your participation in this research is entirely voluntary. If you have questions about the study, please feel free to ask; the contact information for the study leader is: ___________________________
   1. I consent, begin the study
   2. I do not consent, I do not wish to participate
3. Are you a trainee?
   1. No
   2. Yes, I’m a professional student (but not studying to be a physician)
   3. Yes, I’m a medical student
   4. Yes, I’m a resident
   5. Yes, I’m a fellow
4. What is your training background? Select all completed training programs that apply.
   1. General Pediatrics Residency
   2. Medicine-Pediatrics Residency
   3. Pediatric Hospital Medicine Fellowship
   4. Family Medicine Residency
   5. Obstetrics and Gynecology Residency
   6. Maternal Fetal Medicine Residency
   7. Neonatology Fellowship
   8. Child Abuse Pediatrics Fellowship
   9. Bioethicist
   10. Advanced Practice Provider
   11. Nursing
   12. Lawyer
   13. Health Policy
   14. Healthcare Administration
   15. Other________
5. Do you currently work in a practice setting where you might order toxicology testing (urine, meconium, cord, etc) on a newborn during the birth hospitalization?
   1. Yes
   2. No
   3. Other (Please specify) _________________________________________________
6. Did attending this workshop change the way you talk about newborn toxicology testing with others?
   1. Yes
      1. (if “Yes” selected): How has the way you talk about newborn toxicology testing changed?_______________________________________________________
   2. No
7. After attending this workshop, did you…(select all that apply)
   1. Further discuss this topic with another workshop participant after leaving the workshop
   2. Start a conversation with a colleague who didn’t attend about what you learned
   3. Seek patient family perspective on newborn toxicology testing at my institution
   4. Seek out your institution’s process for newborn toxicology testing
   5. Change your practice related to newborn toxicology testing
      1. (if “e” selected) How have you changed your practice since attending this workshop?__________________________________________________
   6. Seek to improve your institution’s approach
      1. (if “f” selected) How have you sought to improve your institution’s approach since attending this workshop?__________________________________
   7. Collaborate on a new research or QI project regarding equity in newborn toxicology testing
   8. Advocate within a professional organization for policy change
   9. Other___________________
   10. None of the above
8. In your opinion, what were the strengths of this workshop?

___________________________________________

1. In your opinion, how could this workshop be improved?

___________________________________________
