# Supplementary material for: How Can Newborn Toxicology Testing Be More Equitable? An Interactive Ethics Workshop
Source: MedEdPORTAL. 2024 Sep 10;20:11434. doi: 10.15766/mep_2374-8265.11434 (PMC11383834; doi:10.15766/mep_2374-8265.11434)
Supplement: Supplementary file 1 — Newborn Toxicology Workshop Slides.pptxParticipant Workbook.docxFacilitator Guide.docxSurvey 1.docxSurvey 2.docx [file mep_2374-8265.11434-s001.zip › C. Facilitator Guide.docx]

**How Can Newborn Toxicology Testing be More Equitable?**

**Facilitator Guide**

**Case Assignments for Small Group Facilitation:**

| **Case** | **Facilitator 1 Name** | **Facilitator 2 Name** | **Pages of Relevance** |
| --- | --- | --- | --- |
| **1** |  |  | Facilitator’s Guide pages 3-6  Participant Workbook page 4 |
| **2** |  |  | Facilitator’s Guide pages 7-10  Participant Workbook page 5 |
| **3** |  |  | Facilitator’s Guide pages 11-14  Participant Workbook page 6 |
| **4** |  |  | Facilitator’s Guide pages 15-18  Participant Workbook page 7 |

**second facilitator for each small group is optional.*

**Facilitators should review the accompanying slide deck prior to the session.**

**Facilitators should bring to the session:**

- Facilitator Guide (this document)
- Copies of “Participant Workbook” to distribute to small group participants at the start of the workshop (digital or paper)
- A time-keeping device (watch, phone, etc)

**Logistical Tips:**

- Workshop Agenda is included on page 2 of this Facilitator Guide
- The Participant Workbook can be distributed electronically or in paper copy form
- Large group polling questions can be implemented by the workshop leader reading the polling question and asking participants to raise hands for their preferred choice(s), or by utilizing an electronic polling interface such as Poll Everywhere©
- Case conclusions appear in this facilitator guide after Discussion Question #4 for each case. Facilitators may share information from the case conclusion with their small group whenever relevant to the discussion.

| **Timing** | **Agenda Item** | **Activity Type** |
| --- | --- | --- |
| **8:00-8:05a**  **(5 min)** | Session Introduction:  *Review objectives, scope, expectations* | Large Group Didactic  *Slides 1-4* |
| **8:05-8:15a**  **(10 min)** | Personal Experience:  *Prompt attendees to reflect on personal experience with this topic* | Large Group Audience Polling #1-4  Small Group Introductions  *Slides 5-10* |
| **8:15-8:20a**  **(5 min)** | Toxicology Testing Indications and Benefits:  *Review relevant literature and practice trends* | Large Group Didactic  *Slides 11-16* |
| **8:20-8:25a**  **(5 min)** | Toxicology Testing Risks and Limitations:  *Review relevant literature and practice trends* | Large Group Didactic  *Slides 17-27* |
| **8:25-8:40a**  **(15 min)** | Discussion Questions 1&2  *Small groups use assigned case narrative as basis for discussion* | Small Group Discussion *moderated by at minimum one facilitator. Group size dependent on number of attendees.*  *Slides 28-31* |
| **8:40-8:45a**  **(5 min)** | Small Groups Report to Large Group, Questions 1 & 2  *Workshop leader requests summary statements from each small group* | Small Group Report to Large Group  *Slide 32* |
| **8:45-9:00a**  **(15 min)** | Ethical Framework  *Review variety of ethical frameworks that may be applied to this topic* | Large Group Didactic  *Slides 33-55* |
| **9:00-9:15a**  **(15 min)** | Discussion Question 3 & 4  *Small groups use assigned case narrative as basis for discussion* | Small Group Discussion *moderated by at minimum one facilitator. Group size dependent on number of attendees.*  *Slides 56-59* |
| **9:15-9:20a**  **(5 min)** | Small Groups Report to Large Group, Questions 3 & 4  *Workshop leader requests summary statements from each small group* | Small Group Report to Large Group  *Slide 60* |
| **9:20-9:25a**  **(5 min)** | Closing and Next Steps  *Workshop leader prompts audience to reflect and commit to actions beyond attending this workshop* | Commitment for Action via polling  *Slides 61-63* |
| **9:25-9:30a** | Questions and Answers | Panel Composed of Workshop Co-Leaders  *Slide 64* |

**How Can Newborn Toxicology Testing be More Equitable?**

**Agenda**

**Case #1**

**First Small Group Discussion**

(15 minutes)

Armani was delivered at term after an unremarkable pregnancy. Her mother, Jada, had normal prenatal care including ultrasounds and lab work. During her second day of life, Armani develops jitteriness and hypertonia. She’s hypoglycemic but her symptoms do not resolve with dextrose gel and normalization of her blood glucose. She’s transferred to the neonatal intensive care unit for further evaluation and treatment. You review the pregnancy history with Jada and she denies opioid or other substance use during pregnancy. Given the severity of Armani’s neurologic symptoms, you’re considering performing a lumbar puncture, ordering an EEG and brain MRI.

1. **Are there indications to obtain toxicology testing from Armani? In your opinion, which indications are valuable or high yield?**

*Suggested Questions for Facilitators to Pose to their Small Group to Guide Conversation:*

- How could newborn toxicology testing results inform clinical management of Armani’s care?
  - If a toxicology (tox) test result can influence the need for a lumbar puncture (LP), electroencephalogram (EEG), or magnetic resonance imaging (MRI), it could be useful. Evaluations such as a LP, EEG, MRI, etc are more invasive and costly than a toxicology test. For the purposes of this discussion, it can be presumed that there are no other clinical symptoms or evidence of sepsis for Armani.
- If Armani’s toxicology testing is positive, does this change your plan of care? What if Armani’s toxicology testing is negative?
  - Regardless of the results of the toxicology test, a clinician is able to make a clinical diagnosis of NOWS and initiate first line nonpharmacologic treatment. Since the newborn is 2 – 3 days old, accuracy of a newborn’s urine and meconium test, the latter being highly dependent on collection technique, is limited. Some delivery hospitals are able to test a sample of umbilical cord tissue within a timeframe of days after birth, which may have higher sensitivity. Obtaining a maternal urine tox test could help with triangulation verification but would reflect medications received during labor (even those received via epidural). Lastly, some labs may need to send out certain tox tests and the turn-around time on the result will also need to be considered. It would not be safe to delay an LP or EEG for a toxicology test result if the clinical exam is concerning for meningitis or seizures rather than NOWS.

1. **What are the risks or limitations to toxicology testing for Armani? How do you balance the potential benefits and harms?**

*Suggested Questions for Facilitators to Pose to their Small Group to Guide Conversation:*

- Risks
  - From the family’s perspective?
    - fear of being stigmatized, anxiety of child protective service (CPS) engagement, worry of legal implications and the impact this may have on other children, employment, etc.
  - From the clinician’s perspective?
    - potential of jeopardizing trust with the family or jeopardizing family engagement in newborn care
  - From an institutional perspective?
    - Balancing clinician decision making with responsibility to local institutional or legal frameworks
    - Missing a diagnosis identifiable via testing could lead to patient harm and be considered negligent care.
  - From a population health perspective?
    - exacerbation of health disparities as younger, Medicaid, and minority populations have been identified to be at increased risk for inequitable care due to unconscious biases.
- Limitations
  - What information is a toxicology test unable to tell you in this case?
    - Toxicology testing detects the presence of a substance but cannot tell you the source or chronicity of exposure. There is also the possibility of multiple pathologic processes being underway, so it is important for a toxicology test to be interpreted in the context of a differential diagnosis.
  - What factors might influence the accuracy or interpretation of the toxicology testing result?
    - Accuracy of a newborn’s urine and meconium test, the latter being highly dependent on collection technique, have been called into question. Some delivery hospitals may test a sample of umbilical cord tissue within a timeframe of days after delivery, which may have higher sensitivity. Discussions with your hospital lab can clarify concerns and ensure optimization. Turn-around time on testing results may preclude their usefulness for timely medical management decisions.
- Alternatives
  - What information can screening questionnaires or conversations provide? What information can’t be ascertained through this approach?
    - Screening questionnaires are crucial to building rapport with families, especially on topics which are currently stigmatized and on which implicit biases have been shown to lead to disparate care. There is potential for more nuanced information to be obtained via questionnaire or interview than that provided by a tox test.

**Case #1**

**Second Small Group Discussion**

(15 minutes)

Armani was delivered at term after an unremarkable pregnancy. Her mother, Jada, had normal prenatal care including ultrasounds and lab work. During her second day of life, Armani develops jitteriness and hypertonia. She’s hypoglycemic but her symptoms do not resolve with dextrose gel and normalization of her blood glucose. She’s transferred to the neonatal intensive care unit for further evaluation and treatment. You review the pregnancy history with Jada and she denies opioid or other substance use during pregnancy. Given the severity of Armani’s neurologic symptoms, you’re considering performing a lumbar puncture, ordering an EEG and brain MRI.

1. **What are additional ethical considerations regarding toxicology testing for Armani?**

*Suggested Questions for Facilitators to Pose to their Small Group to Guide Conversation:*

- What ethical framework fits well with this case in your opinion?
  - Respect for Autonomy: (Slide 40-41)
    - Disclosure and/or parental permission: the parent has a right to make decisions for their child
  - Beneficence: (Slide 42)
    - The infant has a right to be free from suffering
    - The medical team has a duty to the patient, which includes consideration of the family
  - Nonmaleficence: (Slide 42)
    - The medical team has a duty to do no harm through neglectful actions
  - Justice: (Slide 43)
    - Is this how another family or infant would be treated?
  - Feminist Ethics: (Slide 45)
    - How might broader hierarchical social systems limit the autonomy of individuals in this case?
  - Narrative Ethics: (Slide 46-47)
    - How will this family tell their infant’s “birth story”?
  - Relational Ethics: (Slide 48)
    - How can the therapeutic alliance between family and health care clinicians be prioritized?
  - Trauma-Informed Care: (Slide 49)
    - Does Jada feel safe to engage with the healthcare team?
- What cognitive biases may complicate decision-making in this case? (Slide 36)
  - Anchoring Bias: Does one piece of information dominate decision-making?
  - Implicit Bias: How can clinicians guard against implicit bias in this case?

1. **How would you discuss newborn toxicology testing with Jada, Armani’s parent?**

*Suggested Questions for Facilitators to Pose to their Small Group to Guide Conversation:*

- What is the role of informed parental permission or parental assent?
  - A newborn cannot provide consent for toxicology testing. Informed parental permission or parental assent requires disclosure of the indications, benefits, and risks of an intervention. A risk of newborn toxicology testing is mandatory reporting of positive toxicology testing results to CPS. If informed assent is pursued, it is important to disclose what the plan of care would be after a parent declines a newborn toxicology test. If engaging CPS is a planned response to a declined toxicology test, this plan should be included in the informed assent process. If a clinician plans to proceed with a test due to medical necessity regardless of parental opinion, then a framework of disclosure rather than informed assent can be helpful.
- How can clinicians facilitate a conversation that conveys respect?
  - For example, providing time for a parent to consider their path and ask follow-up questions is an expression of respect.
- How can clinicians facilitate transparency?
  - For example, the health care team can map out what the process would look like no matter which path a parent chooses.

*Case facilitators may share the case conclusion with their small group whenever relevant to the discussion.*

**Case Conclusion:** Armani’s urine toxicology test was positive for oxycodone. Jada subsequently acknowledges using non-prescribed opioids during pregnancy. Armani responds well to nonpharmacologic management of her opioid withdrawal.

**Case #2**

**First Small Group Discussion**

(15 minutes)

Olivia was delivered at term. Her mother, Emma’s, pregnancy was notable for a paucity of prenatal care – she only received a dating ultrasound. This ultrasound was performed during a hospitalization for substance withdrawal while Emma was in police custody. She reported using heroin and cocaine during her pregnancy. Emma’s urine toxicology testing at time of delivery was positive for heroin, fentanyl, and cocaine. Olivia is admitted to the NICU with respiratory distress during her first day of life.

1. **Are there indications to obtain toxicology testing from Olivia? In your opinion, which indications are valuable or high yield?**

*Suggested Questions for Facilitators to Pose to their Small Group to Guide Conversation:*

- How could newborn toxicology testing results inform clinical management of Olivia’s care?
  - For this scenario, a maternal positive toxicology test has already revealed short acting opioid exposure, in addition to other substances. Furthermore, disclosure of polysubstance use has already been established. Evaluations such as supplemental oxygen and chest radiographs for the newborn’s respiratory distress may be necessary regardless of any newborn toxicology test result.
- If Olivia’s toxicology testing is positive, does this change your plan of care? What if Olivia’s toxicology testing is negative?
  - Based on maternal history, disclosure and toxicology test results, the newborn qualifies for observation (ie, extended birth hospitalization stay) and management of Neonatal Opioid Withdrawal Syndrome (NOWS). A positive newborn toxicology test would not change this plan, but some states ask or require a toxicology test be obtained on the newborn. If the tox test were negative, it would be highly concerning to be a false negative and a period of observation for NOWS would still be indicated.

1. **What are the risks or limitations to toxicology testing for Olivia? How do you balance the potential benefits and harms?**

*Suggested Questions for Facilitators to Pose to their Small Group to Guide Conversation:*

- Risks
  - From the family’s perspective?
    - fear of being stigmatized, anxiety of child protective service (CPS) engagement, worry of legal implications and the impact this may have on other children, employment, etc.
  - From the clinician’s perspective?
    - potential of jeopardizing trust with the family or jeopardizing family engagement in newborn care
  - From an institutional perspective?
    - Balancing clinician decision making with responsibility to local institutional or legal frameworks
    - Missing a diagnosis identifiable via testing could lead to patient harm and be considered negligent care.
  - From a population health perspective?
    - exacerbation of health disparities as younger, Medicaid, and minority populations have been identified to be at increased risk for inequitable care due to unconscious biases.
- Limitations
  - What information is a toxicology test unable to tell you in this case?
    - Toxicology testing detects the presence of a substance but cannot tell you the source or chronicity of exposure. For example, a positive fentanyl result from a newborn urine specimen could reflect prescribed fentanyl used for pain control during labor (including epidural anesthesia) or could reflect prenatal non-prescribed fentanyl exposure in the hours to days prior to delivery. There is also the possibility of multiple pathologic processes being underway – for example, Olivia likely has a co-morbid cardiorespiratory condition necessitating NICU admission – so it is important for a toxicology test to be interpreted in the context of the differential diagnosis.
  - What factors might influence the accuracy or interpretation of the toxicology testing result?
    - Accuracy of a newborn’s urine and meconium test, the latter being highly dependent on collection technique, have been called into question. Delivery hospitals may test a sample of umbilical cord tissue within a limited timeframe, which may have higher sensitivity. Discussions with your hospital lab can clarify concerns and ensure optimization. Turn-around time on testing results may preclude their usefulness for timely medical management decisions.
- Alternatives
  - What information can screening questionnaires or conversations provide? What information can’t be ascertained through this approach?
    - Screening questionnaires are crucial to building rapport with families, especially on topics which are currently stigmatized and on which implicit biases have been shown to lead to disparate care. There is potential for more nuanced information to be obtained via questionnaire or interview than that provided by a tox test.

**Case #2**

**Second Small Group Discussion**

(15 minutes)

Olivia was delivered at term. Her mother, Emma’s, pregnancy was notable for a paucity of prenatal care – she only received a dating ultrasound. This ultrasound was performed during a hospitalization for substance withdrawal while Emma was in police custody. She reported using heroin and cocaine during her pregnancy. Emma’s urine toxicology testing at time of delivery was positive for heroin, fentanyl, and cocaine. Olivia is admitted to the NICU with respiratory distress on during her first day of life.

1. **What are additional ethical considerations regarding toxicology testing for Olivia?**

*Suggested Questions for Facilitators to Pose to their Small Group to Guide Conversation:*

- What ethical framework fits well with this case in your opinion?
  - Respect for Autonomy: (Slide 40-41)
    - Disclosure and/or parental permission: the parent has a right to make decisions for their child
  - Beneficence: (Slide 42)
    - The infant has a right to be free from suffering
    - The medical team has a duty to the patient, which includes consideration of the family
  - Nonmaleficence: (Slide 42)
    - The medical team has a duty to do no harm through neglectful actions
  - Justice: (Slide 43)
    - Is this how another family or infant would be treated?
  - Feminist Ethics: (Slide 45)
    - How might broader hierarchical social systems limit the autonomy of individuals in this case?
  - Narrative Ethics: (Slide 46-47)
    - How will this family tell their infant’s “birth story”?
  - Relational Ethics: (Slide 48)
    - How can the therapeutic alliance between family and health care clinicians be prioritized?
  - Trauma-Informed Care: (Slide 49)
    - Does Emma feel safe to engage with the healthcare team?
- What cognitive biases may complicate decision-making in this case? (Slide 36)
  - Anchoring Bias: Does one piece of information dominate decision-making?
  - Implicit Bias: How can clinicians guard against implicit bias in this case?

1. **How would you discuss newborn toxicology testing with Emma, Olivia’s parent?**

*Suggested Questions for Facilitators to Pose to their Small Group to Guide Conversation:*

- What is the role of informed parental permission or assent?
  - A newborn cannot provide consent for toxicology testing. Informed parental permission or parental assent requires disclosure of the indications, benefits, and risks of an intervention. A risk of newborn toxicology testing is mandatory reporting of positive toxicology testing results to CPS. If informed assent is pursued, it is important to disclose what the plan of care would be after a parent declines a newborn toxicology test. If engaging CPS is a planned response to a declined toxicology test, this plan should be included in the informed assent process. If a clinician plans to proceed with a test due to medical necessity regardless of parental opinion, then a framework of disclosure rather than informed assent can be helpful.
- How can clinicians facilitate a conversation that conveys respect?
  - For example, providing time for a parent to consider their path and ask follow-up questions is an expression of respect.
- How can clinicians facilitate transparency?
  - For example, the health care team can map out what the process would look like no matter which path a parent chooses.

*Case facilitators may share the case conclusion with their small group whenever relevant to the discussion.*

**Case Conclusion:** Olivia’s urine toxicology testing was positive only for cocaine. Her respiratory distress, attributed to TTN, resolved. Olivia developed symptoms consistent with opioid withdrawal during her second day of life. She responded well to nonpharmacologic, supportive care.

**Case #3**

**First Small Group Discussion**

(15 minutes)

Clara is a term newborn delivered vaginally after an unremarkable pregnancy. Throughout the pregnancy, her mother, Gabriela, told her OB that she was using cannabis to improve her appetite and help her sleep. Maternal urine toxicology was positive for cannabis throughout pregnancy and at delivery. You consult social work who reports that there is no prior Child Protective Services (CPS) involvement for Gabriela’s two, older children.

1. **Are there indications to obtain toxicology testing from Clara? In your opinion, which indications are valuable or high yield?**

*Suggested Questions for Facilitators to Pose to their Small Group to Guide Conversation:*

- How does newborn toxicology testing results change the inform / impact clinical management of Clara’s care?
  - Though each state may have different positions on the legalization of cannabis, it remains a federally illegal substance. Because of this variation state-to-state, the law on toxicology (tox) testing the infant who has been exposed to prenatal cannabis is not uniform. It has been shown that among pregnant patients who disclose sole cannabis use during pregnancy, approximately 1% may be withholding information on using other illicit / inappropriate substances. Thus, testing the newborn to discern whether they were exposed to other substances which would affect the newborn hospitalization, e.g. opioids, would be low yield and opposite to high-value care models.
- If Clara’s toxicology testing is positive, does this change your plan of care? What if Clara’s toxicology testing is negative?
  - Counseling on the potential for harm with perinatal cannabis use, especially for women wanting to breastfeed, would be indicated given the maternal disclosure. As perinatal cannabis exposure has not been shown to have acute neonatal effects, clinical care during the birth hospitalization would remain the same if a newborn tox test were positive or negative.

1. **What are the risks or limitations to toxicology testing for Clara? How do you balance the potential benefits and harms?**

*Suggested Questions for Facilitators to Pose to their Small Group to Guide Conversation:*

- Risks
  - From the family’s perspective?
    - fear of being stigmatized, anxiety of child protective service (CPS) engagement, worry of legal implications and the impact this may have on other children, employment, etc.
  - From the clinician’s perspective?
    - potential of jeopardizing trust with the family or jeopardizing family engagement in newborn care
  - From an institutional perspective?
    - Balancing clinician decision making with responsibility to local institutional or legal frameworks
    - Missing a diagnosis identifiable via testing could lead to patient harm and be considered negligent care.
  - From a population health perspective?
    - exacerbation of health disparities as younger, Medicaid, and minority populations have been identified to be at increased risk for inequitable care due to unconscious biases.
- Limitations
  - What information is a toxicology test unable to tell you in this case?
    - The presence of THC in a tox test does not inform one on when the substance was last used as THC is known to linger in biologic samples for days to weeks after last reported use.
  - What factors might influence the accuracy or interpretation of the toxicology testing result?
    - Accuracy of newborn urine and meconium testing, the latter being highly dependent on collection technique, has been called into question. Some delivery hospitals are able to tox test a sample from the umbilical cord within a limited timeframe, which may have higher sensitivity. Discussions with your hospital lab can clarify concerns and ensure optimization. Lastly, some labs may need to send out certain tox tests and the turn-around time on the result will also need to be considered.
    - Interpretation of prior CPS involvement needs to be made with caution and sensitivity. At its core, CPS is a supportive agency aiming to support families and the presence of their involvement should not be taken as a marker of parental abuse or neglect. Lastly, supporting families with locally available resources does not obligate involvement of CPS and may be facilitated by health care clinicians, with the assistance of social workers if available to you.
- Alternatives
  - What information can screening questionnaires or conversations provide? What information can’t be ascertained through this approach?
    - Screening questionnaires are crucial to building rapport with families, especially on topics which are currently stigmatized and on which implicit biases have been shown to lead to disparate care. There is potential for more nuanced information to be obtained via questionnaire or interview than that provided by a tox test.

**Case #3**

**Second Small Group Discussion**

(15 minutes)

Clara is a term newborn delivered vaginally after an unremarkable pregnancy. Throughout the pregnancy, her mother, Gabriela, told her OB that she was using cannabis to improve her appetite and help her sleep. Maternal urine toxicology was positive for cannabis throughout pregnancy and at delivery. You consult social work who reports that there is no prior Child Protective Services (CPS) involvement for Gabriela’s two, older children.

1. **What are additional ethical considerations regarding toxicology testing for Clara?**

*Suggested Questions for Facilitators to Pose to their Small Group to Guide Conversation:*

- What ethical framework fits well with this case in your opinion?
  - Respect for Autonomy: (Slide 40-41)
    - Disclosure and/or parental permission: the parent has a right to make decisions for their child
  - Beneficence: (Slide 42)
    - The infant has a right to be free from suffering
    - The medical team has a duty to the patient, which includes consideration of the family
  - Nonmaleficence: (Slide 42)
    - The medical team has a duty to do no harm through neglectful actions
  - Justice: (Slide 43)
    - Is this how another family or infant would be treated?
  - Feminist Ethics: (Slide 45)
    - How might broader hierarchical social systems limit the autonomy of individuals in this case?
  - Narrative Ethics: (Slide 46-47)
    - How will this family tell their infant’s “birth story”?
  - Relational Ethics: (Slide 48)
    - How can the therapeutic alliance between family and health care clinicians be prioritized?
  - Trauma-Informed Care: (Slide 49)
    - Does Gabriela feel safe to engage with the healthcare team?
- What cognitive biases may complicate decision-making in this case? (Slide 36)
  - Anchoring Bias: Does one piece of information dominate decision-making?
  - Implicit Bias: How can clinicians guard against implicit bias in this case?

1. **How would you discuss newborn toxicology testing with Gabriela, Clara’s parent?**

*Suggested Questions for Facilitators to Pose to their Small Group to Guide Conversation:*

- What is the role of informed parental permission or parental assent?
  - A newborn cannot provide consent for toxicology testing. Informed parental permission or parental assent requires disclosure of the indications, benefits, and risks of an intervention. A risk of newborn toxicology testing is mandatory reporting of positive toxicology testing results to CPS. If informed assent is pursued, it is important to disclose what the plan of care would be after a parent declines a newborn toxicology test. If engaging CPS is a planned response to a declined toxicology test, this plan should be included in the informed assent process. If a clinician plans to proceed with a test due to medical necessity regardless of parental opinion, then a framework of disclosure rather than informed assent can be helpful.
- How can clinicians facilitate a conversation that conveys respect?
  - For example, providing time for a parent to consider their path and ask follow-up questions is an expression of respect.
- How can clinicians facilitate transparency?
  - For example, the health care team can map out what the process would look like no matter which path a parent chooses.

*Case facilitators may share the case conclusion with their small group whenever relevant to the discussion.*

**Case Conclusion:** Social work does not request toxicology testing on Clara and you do not order any. CPS referral is not placed. Clara is discharged home with her parents during her second day of life.

**Case #4**

**First Small Group Discussion**

(15 minutes)

Li is delivered at term after a pregnancy notable for an absence of prenatal care. Li’s mother, Xiang (pronounced SHYAHNG), reports that she unsuccessfully attempted to make appointments. Given this history, your colleagues order urine toxicology testing on Xiang at time of delivery. Her test is positive for cocaine and she states that it is a “mystery” to her how this could be possible. Social work reports this to Child Protective Services (CPS) and the CPS supervisor requests testing on Li.

1. **Are there indications to obtain toxicology testing from Li? In your opinion, which indications are valuable or high yield?**

*Suggested Questions for Facilitators to Pose to their Small Group to Guide Conversation:*

- How does newborn toxicology testing results change the inform / impact clinical management of Li’s care?
  - It can be presumed that Li is well-appearing for this case. There is variation in legal mandates between states with regards to obtaining a toxicology (tox) test on a newborn. Some states may require it in a scenario like this. Other states may not. From a medical perspective, prenatal cocaine exposure is an important historical detail to consider in holistic assessment of the newborn but does not precipitate withdrawal symptoms requiring extended observation or pharmacologic intervention, in contrast to chronic prenatal opioid exposure. Clinicians can provide counseling on substances and breastfeeding with Xiang regardless of Li’s toxicology test result.
- If Li’s toxicology testing is positive, does this change your plan of care? What if Li’s toxicology testing is negative?
  - Additional tox testing of urine, meconium and / or the umbilical cord tissue could add additional information, but each also harbor imperfect results. If Li’s tox test were obtained and positive for an opiate, in the context of a negative maternal urine tox test for opioids, Li’s result could be a false positive or reflect opioids administered to Xiang during labor (including via epidural). Challenges with collecting an adequate meconium sample, the available timeframe to test the umbilical cord, and sending out tests to other labs can lead to delays or influence the accuracy of a test. A negative tox test on Li, however, could help substantiate Xiang’s view that the cocaine identified in her urine was a false positive.

1. **What are the risks or limitations to toxicology testing for Li? How do you balance the potential benefits and harms?**

*Suggested Questions for Facilitators to Pose to their Small Group to Guide Conversation:*

- Risks
  - From the family’s perspective?
    - fear of being stigmatized, anxiety of child protective service (CPS) engagement, worry of legal implications and the impact this may have on other children, employment, etc.
  - From the clinician’s perspective?
    - potential of jeopardizing trust with the family or jeopardizing family engagement in newborn care
  - From an institutional perspective?
    - Balancing clinician decision making with responsibility to local institutional or legal frameworks
    - Missing a diagnosis identifiable via testing could lead to patient harm and be considered negligent care.
  - From a population health perspective?
    - exacerbation of health disparities as younger, Medicaid, and minority populations have been identified to be at increased risk for inequitable care due to unconscious biases.
- Limitations
  - What information is a toxicology test unable to tell you in this case?
    - Toxicology testing detects the presence of a substance but cannot tell you the source or chronicity of exposure.
  - What factors might influence the accuracy or interpretation of the toxicology testing result?
    - Accuracy of toxicology testing is influenced by specimen collection, method of analysis and confirmation testing, the assay’s false positive/negative rate, and timing of maternal last use of a substance, among other factors. Discussions with your hospital lab can clarify concerns and ensure optimization.
- Alternatives
  - What information can screening questionnaires or conversations provide? What information can’t be ascertained through this approach?
    - Screening questionnaires are crucial to building rapport with families, especially on topics which are currently stigmatized and on which implicit biases have been shown to lead to disparate care. There is potential for more nuanced information to be obtained via questionnaire or interview than that provided by a tox test.

**Case #4**

**Second Small Group Discussion**

(15 minutes)

Li is delivered at term after a pregnancy notable for an absence of prenatal care. Li’s mother, Xiang (pronounced SHYAHNG), reports that she unsuccessfully attempted to make appointments. Given this history, your colleagues order urine toxicology testing on Xiang at time of delivery. Her test is positive for cocaine and she states that it is a “mystery” to her how this could be possible. Social work reports this to Child Protective Services (CPS) and the CPS supervisor requests testing on Li.

1. **What are additional ethical considerations regarding toxicology testing for Li?**

*Suggested Questions for Facilitators to Pose to their Small Group to Guide Conversation:*

- What ethical framework fits well with this case in your opinion?
  - Respect for Autonomy: (Slide 40-41)
    - Disclosure and/or parental permission: the parent has a right to make decisions for their child
  - Beneficence: (Slide 42)
    - The infant has a right to be free from suffering
    - The medical team has a duty to the patient, which includes consideration of the family
  - Nonmaleficence: (Slide 42)
    - The medical team has a duty to do no harm through neglectful actions
  - Justice: (Slide 43)
    - Is this how another family or infant would be treated?
  - Feminist Ethics: (Slide 45)
    - How might broader hierarchical social systems limit the autonomy of individuals in this case?
  - Narrative Ethics: (Slide 46-47)
    - How will this family tell their infant’s “birth story”?
  - Relational Ethics: (Slide 48)
    - How can the therapeutic alliance between family and health care clinicians be prioritized?
  - Trauma-Informed Care: (Slide 49)
    - Does Xiang feel safe to engage with the healthcare team?
- What cognitive biases may complicate decision-making in this case? (Slide 36)
  - Anchoring Bias: Does one piece of information dominate decision-making?
  - Implicit Bias: How can clinicians guard against implicit bias in this case?

1. **How would you discuss newborn toxicology testing with Xiang, Li’s parent?**

*Suggested Questions for Facilitators to Pose to their Small Group to Guide Conversation:*

- What is the role of informed parental permission or parental assent?
  - A newborn cannot provide consent for toxicology testing. Informed parental permission or parental assent requires disclosure of the indications, benefits, and risks of an intervention. A risk of newborn toxicology testing is mandatory reporting of positive toxicology testing results to CPS. If informed assent is pursued, it is important to disclose what the plan of care would be after a parent declines a newborn toxicology test. If engaging CPS is a planned response to a declined toxicology test, this plan should be included in the informed assent process. If a clinician plans to proceed with a test due to medical necessity regardless of parental opinion, then a framework of disclosure rather than informed assent can be helpful.
- How can clinicians facilitate a conversation that conveys respect?
  - For example, providing time for a parent to consider their path and ask follow-up questions is an expression of respect.
- How can clinicians facilitate transparency?
  - For example, the health care team can map out what the process would look like no matter which path a parent chooses.

*Case facilitators may share the case conclusion with their small group whenever relevant to the discussion.*

**Case Conclusion:** Li remains clinically well. Xiang desires breastfeeding but is advised to stop breastfeeding and feed Li formula. Li’s urine toxicology testing is negative for all substances including cocaine.
