# Supplementary material for: How Can Newborn Toxicology Testing Be More Equitable? An Interactive Ethics Workshop
Source: MedEdPORTAL. 2024 Sep 10;20:11434. doi: 10.15766/mep_2374-8265.11434 (PMC11383834; doi:10.15766/mep_2374-8265.11434)
Supplement: Supplementary file 1 — Newborn Toxicology Workshop Slides.pptxParticipant Workbook.docxFacilitator Guide.docxSurvey 1.docxSurvey 2.docx [file mep_2374-8265.11434-s001.zip › _Educational Summary Report_11434.pdf]

MedEdPORTAL® The AAMC Journal of Teaching and Learning Resources

Original Publication

Open Access

How Can Newborn Toxicology Testing Be More Equitable? An Interactive Ethics Workshop

Kelsey Ryan, MD*, Rakhi Gupta Basuray, MD, Christy Cummings, MD

*Corresponding author: keryan@mcw.edu

Abstract

Introduction: Practice variation in newborn toxicology testing during the birth hospitalization exists across institutions and legal jurisdictions. While testing can provide benefits, indiscriminate testing has been shown to perpetuate health care inequities. In the backdrop of an opioid epidemic and a charged medicolegal landscape, this workshop guides participants to reexamine newborn toxicology testing through a shared ethical lens. **Methods:** We conducted a live, 90-minute workshop in English at an international pediatric conference. Physicians, residents, and fellows participated in large- and small-group breakout sessions to learn relevant clinical and bioethical frameworks, share their own local context and expertise, and explore ethical applications through case-based discussions. We administered two anonymous online follow-up surveys to assess self-perceived impact on participant knowledge, behavior, and clinical practice. **Results:** Seven facilitators and 45 individuals participated in the workshop. Eighteen participants completed survey 1 immediately following workshop conclusion, and six participants completed survey 2 after 3 months had elapsed. Immediately following the workshop, 94% of respondents reported that they had been introduced to a new idea, and 82% were considering practice change. A low response rate to survey 2 limited interpretation, but some respondents reported self-perceived change following workshop attendance. **Discussion:** This workshop facilitated conversation between physician participants on a complex pediatric health care inequity issue using an ethical framework.

Keywords:
Equity, Newborn, Social Constructivism, Toxicology, Ethics/Bioethics, Health Equity, Neonatal-Perinatal Medicine, Pediatrics

Educational Objectives

By the end of this activity, learners will be able to:

1. Assess the potential benefits and harms of current and alternative approaches to newborn toxicology testing.
2. Analyze varied approaches to newborn toxicology testing with regard to informed consent, bias, and justice.
3. Leverage ethical analysis to reflect critically upon their own practice environment and identify actionable areas for change and/or advocacy.

Introduction

Newborn toxicology testing is recommended when it informs clinical management of the neonate.[1,2] Institutions and clinicians operationalize this recommendation differently. Universal toxicology testing of pregnant patients is not recommended by professional medical organizations due to evidence of downstream biases in clinical care and child protective service referrals.[3,4] Furthermore, the accuracy and reliability of toxicology testing have been challenged. Methods used to collect samples, interpretation of results on timing and duration of fetal exposure, and efficiency of result reporting are fraught with caveats.[1] Clinicians and public health officials involved in the care of newborns affected by parental substance use disorder struggle with identifying an appropriate course of action with regard to toxicology testing of newborns and local interpretations of federal legislation that aims to create plans of safe care.[5,6]

The educational tool presented here builds upon existing educational literature supporting learner engagement with issues of health equity through clinical cases illustrating biomedical concepts.[7-10] Returning to principles of biomedical ethics can help manage uncertainty and allows clinicians to answer the call for action articulated in the American Academy of Pediatrics 2020 clinical report on neonatal opioid withdrawal syndrome:

Citation:
Ryan K, Gupta Basuray R, Cummings C. How can newborn toxicology testing be more equitable? An interactive ethics workshop. *MedEdPORTAL*. 2024;20:11434.
https://doi.org/10.15766/mep_2374-8265.11434

Copyright © 2024 Ryan et al. This is an open-access publication distributed under the terms of the Creative Commons Attribution license.

"Pediatricians should be aware of and reduce institutional biases in implementing universal toxicology testing for infants, which could result in unequal consequences for mothers and infants on the basis of race, ethnicity, and/or socioeconomic status."[1,11-13] Our workshop informs participants of ethical constructs related to newborn toxicology testing and empowers them to conceptualize equitable change within their local context using a social constructivism framework.[14]

Methods

Context

Facilitators with expertise in newborn medicine, toxicology, and ethics collaborated in workshop design, implementation, and evaluation. Eight clinician educators with expertise in the care of people affected by substance use disorder, newborn care, neonatology, and pediatric bioethics formed the workshop planning group. We designed our workshop using the framework of social constructivism, or creation of knowledge through interactions with others. We hoped to facilitate collaborative dialogue between individuals with diverse expertise and thereby place participants in their zone of proximal development, where they might critically reappraise their own practice and envision alternative approaches.[14]

Sociocultural theory focused our workshop objectives, methods, and evaluative efforts on identifying whether participants engaged with new ideas and conceptualized future changes in their own practice. Large-group didactic material enabled participants to establish common knowledge, while small-group discussion allowed them to consider varied approaches and critically reflect on their own practice to identify opportunities for change. Live, anonymous, online polling circulated various audience perspectives in real time and prompted participants to contemplate future action. Online, asynchronous, anonymous follow-up surveys were intended to prompt self-assessment of participant reaction, learning, personal behavior change, and change in patient practice using the framework of the New World Kirkpatrick model of assessment.[15,16] Additionally, we applied a net promoter score (NPS) as a validated quantitative marker of engagement with continuing education curricula. NPS was assessed by asking, on a scale of 1-10, the likelihood that a respondent would recommend the activity to friends or colleagues. Responses less than 7 were deemed detractors, 7-8 were passives, and 9-10 were promoters. The NPS score was calculated using the following formula: [percentage of respondents who are promoters] − [percentage of respondents who are detractors]. The score could range from −100 to 100.[17]

Implementation

The workshop was implemented in one 90-minute setting but could be scaled up or down to accommodate varying numbers of facilitators and participants and could be repeated with variation provided by individual audience members and interactive discussions. Planning was performed in anticipation of approximately 50 in-person attendees with a broad range of previous knowledge, skills, and attitudes on the topic, including both trainees and nontrainees, as well as physicians and nonphysicians. We aimed to create a workshop that could facilitate multidisciplinary conversation between medical or nursing students, residents, fellows, attending physicians, nurses, advanced practice providers, social workers, and lactation consultants as well as interested policymakers, lawyers, patient advocates, and/or child protective services personnel. Small groups ranged from three to 10 people, and each small group required at least one facilitator. Ideally, each small group would have had facilitators with newborn clinical experience and/or bioethical expertise. However, the workshop was intentionally created to allow anyone to facilitate after reviewing the workshop materials in advance. The workshop was best conducted in a conference room environment with audiovisual projection capabilities for large-group didactic material and round tables to accommodate small-group case-based discussion.

We conducted the workshop at a large international pediatric conference with physician attendees representing a variety of practice specialty and institutional backgrounds. One individual from the workshop planning group served as the workshop leader, and seven served as workshop facilitators. The workshop leader and all facilitators cared for newborns in their clinical practice, while four workshop leaders had additional bioethical expertise. No prerequisite knowledge was required of learners. Workshop attendance was voluntary, self-selected, and likely influenced by preexisting interest in the topic. Participants self-selected seating, and one or two facilitators were available for each small group. When possible, facilitators were partnered to provide complementary clinical and bioethical expertise on the topic. The workshop leader led the session agenda utilizing a PowerPoint slide deck to transition the audience through alternating large- and small-group activities (Appendix A).[11] Each participant was provided with either a paper or digital copy of the participant workbook, which contained an overview of the session, materials on the four cases for discussion, and additional references (Appendix B). Each small-group table was assigned one case study. Though cases were different, four identical prompts for discussion were offered following each case, unifying the conversation across small groups. Facilitators received

Copyright © 2024 Ryan et al. This is an open-access publication distributed under the terms of the Creative Commons Attribution license.

a facilitator guide in advance; this guide contained logistical instructions, the agenda, and discussion prompts (Appendix C).

The workshop leader used live, anonymous, online polling, hosted by Poll Everywhere, to welcome the large group and illuminate the diversity of expertise in the room.[12] The workshop leader then introduced a series of clinical experts to briefly review the uses and limitations of newborn toxicology testing. These mini-lectures ensured workshop participants entered small-group conversation with a foundation of common knowledge to spark discussion. Participants and facilitators were instructed to read and discuss their table's case using discussion prompts 1 and 2. After approximately 15 minutes, small-group discussion time concluded with the workshop leader asking for a readout, or brief summary, from each table. The audience then returned to a large-group mini-lecture to cover bioethical frameworks applicable to newborn toxicology testing. This was followed by a second small-group breakout session to discuss prompts 3 and 4, with one last readout to the large group. The session concluded with the workshop leader guiding the large group through a reflective live-polling question, asking each participant to commit to next steps, and moderating a question-and-answer session.

Evaluation

To evaluate workshop impact on participants, we designed two follow-up survey tools. Prior to conducting the workshop, we piloted surveys for validity testing with a group of pediatric clinicians with content expertise (neonatal hospitalists) who did not attend the workshop. The Medical College of Wisconsin Institutional Review Board approved collection of workshop participant survey responses with online written informed consent obtained at the start of each survey (PRO00047413 registered as minimal risk category 2 FLEX review). At the conclusion of the workshop, information sheets were distributed to attendees to offer the opportunity to voluntarily participate in research surveys assessing self-assessed workshop impact. Access to the online informed consent and survey 1 was provided by QR code. Survey 1 solicited participants' immediate reactions to the workshop, opened immediately after the in-person workshop completed, and remained open for 14 days (Appendix D). Survey 2 was disseminated 3 months after workshop completion to those survey 1 respondents who shared an email contact; this survey also remained open for 14 days (Appendix E). Surveys were hosted by Qualtrics. All potential participants were advised verbally and in writing that workshop leaders would not know if participants completed any survey and would be masked to respondent identifying information. Participants could disengage from the research at any time, and surveys could be completed asynchronously. Survey results were analyzed in Microsoft Excel.

Results

One workshop leader, seven workshop facilitators, and 45 workshop participants participated in this peer-reviewed workshop at the international Pediatric Academic Societies conference hosted in Washington, DC, in April 2023. A summary of live-polling responses from workshop participants can be found in Table 1. The majority of participants identified a shared background of having completed a general pediatrics residency. Two participants identified as current residents and one as a current fellow; the remainder identified as no longer in training. Ninety-three percent of participants affirmed currently working in a practice setting where they might order toxicology testing on a newborn during the birth hospitalization. When asked what brought them to this workshop, 77% of participants responded, "I'm worried that toxicology testing for newborns may not always

Table 1. Workshop Live-Polling Responses ($N = 45$)

Survey Item	No. (%)
Respondent characteristics	
Training status	
Resident physician	2 (7)
Attending physician	26 (93)
Total unique responses	28 (60)
Completed training (select all that apply)	
General pediatrics residency	28 (96)
Medicine-pediatrics residency	2 (7)
Pediatric hospital medicine fellowship	3 (10)
Neonatology fellowship	8 (28)
Bioethicist	6 (21)
Other	6 (21)
Total unique responses	29 (64)
Current practice	
Order newborn toxicology testing	26 (93)
Do not order newborn toxicology testing	2 (7)
Total unique responses	28 (62)
Motivation (select all that apply)	
I think my institution does newborn toxicology testing well	5 (16)
I'm worried that toxicology testing for newborns may not always be "fair" or "appropriate"	24 (77)
I want to find out what other practitioners are doing in this area	22 (71)
I'm interested in examining a clinical scenario through a bioethical framework	13 (42)
Total unique responses	31 (69)
Respondent reactions	
Commitment to action (select all that apply)	
Share my contact information	11 (30)
Start a conversation with a colleague	30 (83)
Learn institutional approach	13 (36)
Change my practice	11 (30)
Improve institutional approach	28 (78)
Collaborate on research or quality improvement	15 (42)
Advocate for policy change	14 (39)
Other	2 (6)
Total unique responses	36 (80)

Copyright © 2024 Ryan et al. This is an open-access publication distributed under the terms of the Creative Commons Attribution license.

be ‘fair’ or ‘appropriate.”’ At the conclusion of the workshop, when asked to identify personal next steps, 83% of workshop participants intended to “start a conversation with a colleague who did not attend about what I learned here today,” and 78% intended to “seek to improve my institution’s approach.”

Survey 1 received 18 responses (40% response rate of workshop participants), including three who identified as trainees. The results of survey 1 can be reviewed in Table 2. The majority of respondents had a favorable reaction to the workshop, with an NPS of 66.7 and 78% of respondents classifying as promoters. The majority of respondents affirmed learning new content and reported considering practice change after attending the workshop. Free-text responses included positive reflections on the course (two of four), neutral (one of four), and suggestions for improvement (one). For example, one respondent shared the positive reflection “Thank you for being brave.”

Survey 2 received six responses (40% response rate of those who shared contact information in survey 1, 13% of original workshop participants), and all respondents identified as nontrainees. The results of survey 2 can be reviewed in Table 3. Verbatim free-text survey responses are not included in Table 3 to safeguard against unintentional identification of respondents. Five out of six respondents (83%) reported that attending this workshop changed the way they talk about newborn toxicology testing with others. While no respondents reported a change in their personal practice, four respondents reported advocating for change to an institutional or professional organization’s policy. For example, “Engaged the OB staff based on [American College of Obstetricians and Gynecologists] plans to support questionnaire based screening of families. Our hospital uses universal screening.”

Table 2. Responses to Immediate Follow-up Survey 1 ($N = 18$)

Survey Item	No. (%)
Respondent characteristics	
Training status	
Resident physician	2 (11)
Physician fellow	1 (6)
Attending physician	15 (83)
Total unique responses	18 (100)
Completed training (select all that apply)	
General pediatrics residency	16 (89)
Pediatric hospital medicine fellowship	1 (6)
Neonatology fellowship	7 (39)
Bioethicist	1 (6)
Health care administration	1 (6)
Other[a]	3 (17)
Total unique responses	18 (100)
Current practice	
Order newborn toxicology testing	17 (94)
Do not order newborn toxicology testing	1 (6)
Total unique responses	18 (100)
Respondent reactions	
Workshop net promoter score = 66.7	
Promoter (9 or 10)	14 (78)
Passive (7 or 8)	2 (11)
Detractor (0-6)	2 (11)
Total unique responses	18 (100)
Self-assessed change in knowledge	
Workshop introduced new knowledge	17 (94)
Workshop did not introduce new knowledge	1 (6)
Total unique responses	18 (100)
Self-assessed change in behavior	
Considering a change in practice	14 (82)
Not considering a change in practice	2 (12)
Other[b]	1 (6)
Total unique responses	17 (94)

[a]Those who selected “Other” entered the free-text responses “research fellowship,” “health policy research fellowship,” and “health policy.”
[b]The individual who selected “Other” entered the free-text response “maybe.”

Discussion

This workshop facilitates cocreation of knowledge and critical reflection regarding newborn toxicology testing by contextualizing evidence-based medicine and participant experience within bioethical frameworks. The workshop can be carried out in one session, with no prior preparation required, and with a multidisciplinary audience. We report its successful

Table 3. Responses to 3-Month Follow-up Survey ($N = 6$)

Survey Item	No. (%)
Respondent characteristics	
Training status	
Resident physician	0 (0)
Physician fellow	0 (0)
Attending physician	6 (100)
Total unique responses	6 (100)
Completed training (select all that apply)	
General pediatrics residency	6 (100)
Pediatric hospital medicine fellowship	1 (17)
Neonatology fellowship	2 (33)
Total unique responses	6 (100)
Current practice	
Order newborn toxicology testing	5 (83)
Do not order newborn toxicology testing	1 (17)
Total unique responses	6 (100)
Respondent reactions	
Self-assessed change in communication behavior	
The way I talk about this topic has changed	5 (83)
The way I talk about this topic has not changed	1 (17)
Total unique responses	6 (100)
Self-assessed change in collaborative behavior	
Discuss with another workshop participant after the workshop	2 (50)
Discuss with a colleague who didn’t attend	2 (50)
Seek patient family perspective on newborn toxicology testing at my institution	1 (25)
Seek out my institution’s process for newborn toxicology testing	3 (75)
Change my practice related to newborn toxicology testing	0 (0)
Seek to improve my institution’s approach	4 (100)
Collaborate on a new research or quality improvement project regarding equity in newborn toxicology testing	4 (100)
Advocate within a professional organization for policy change	1 (25)
Total unique responses	4 (100)

Copyright © 2024 Ryan et al. This is an open-access publication distributed under the terms of the Creative Commons Attribution license.

implementation with a physician audience in a onetime, in-person setting. Our survey results indicate that, after attending, some participants reconsidered their personal or institutional practice and some reported advocating for change at an institutional or organizational level. our educational resource provides a structured tool for those interested in facilitating constructive conversation on this issue with a biomedical ethical perspective.

We learned from workshop implementation that even amongst pediatric conference attendees who self-selected to attend, a wide variety of newborn toxicology testing practices was reported. This underscores the potential for structured ethical analysis, rather than practice standardization, to be a feasible first step toward consensus building. Facilitators noted that small groups needed encouragement to engage with an ethical lens, as opposed to a probabilistic approach. In the words of one facilitator,

> One thing that was challenging was trying to have people examine the cases from the ethical lens as most at the table were more comfortable with the other concepts/logistics presented. However, we did get time for that and were able to push participants to examine the concept of best interest—is it truly best? And consider other ethical frameworks like narrative ethics for the case.

This suggests that the workshop activities, particularly facilitated small-group discussion, pushed participants to enter their zone of proximal development.[10] Small-group facilitators appreciated having both clinical and biomedical ethics experts cofacilitating the small-group discussion to model the integration of clinical and ethical priorities, suggesting that pairing facilitators of differing backgrounds for each small group may be optimal.

Based upon feedback received from survey responses, we modified workshop materials to aid generalizability and future implementation. Formatting of cases and discussion questions was streamlined in the participant workbook (Appendix B). Reference to feeding choices was added to case 4. Case-specific discussion prompts were added to the facilitator guide as examples of how facilitators might apply an ethical framework in small-group discussion (Appendix C).

Our assessment of this workshop's impact is limited. First, we report our experience with a single implementation that occurred with a self-selected physician audience. We assume geographic and institutional variation amongst this group of workshop participants based upon the general conference attendee population, but we did not collect demographic data. While we aimed for the workshop to be applicable to a wide variety of disciplines, we cannot report its multidisciplinary impact based upon this single implementation. We also observed low survey response rates compounded by significant response attrition. Survey 1's response rate was 40% of workshop attendees, and survey 2's was 13% of workshop attendees. Self-selected workshop attendees may have had existing interest in this topic, predisposing them toward positive responses to the workshop, and such bias would only be amplified by sequential surveys with marked response attrition. Those who self-perceive impact may also be more motivated to respond to follow-up surveys. Finally, self-reported surveys can only report self-perceived behavior change. Due to the many different practice contexts of participants at our workshop, it was logistically infeasible to independently solicit data regarding impact on direct patient care. However, this could be a rich addition to workshop assessment if implemented in a single- or defined practice context.

While the majority of participants reported completing pediatric physician training, this workshop could be of value to anyone providing health care for pregnant people and/or infants, including medical students, residents, fellows, and other health professions trainees, as well as those who interface with health care from affiliated disciplines such as policy or law. This workshop could also be implemented in a variety of contexts, including local or single-institution groups of participants. If participants at a single institution were to implement the workshop, measuring direct patient outcomes such as toxicology testing frequency and referral practices after workshop participation would be a potential next step to assess impact at the highest level of Kirkpatrick's model—results. We hope that dissemination of workshop materials will enable implementation of the workshop across a range of contexts and audiences.

Appendices

A. Newborn Toxicology Workshop Slides.pptx
B. Participant Workbook.docx
C. Facilitator Guide.docx
D. Survey 1.docx
E. Survey 2.docx

All appendices are peer reviewed as integral parts of the Original Publication.

Kelsey Ryan, MD: Associate Professor, Department of Pediatrics, Medical College of Wisconsin; ORCID: https://orcid.org/0000-0002-9836-3963

Copyright © 2024 Ryan et al. This is an open-access publication distributed under the terms of the Creative Commons Attribution license.

Rakhi Gupta Basuray, MD: Assistant Professor, Department of Pediatrics, The Ohio State University College of Medicine, and Division of Pediatric Hospital Medicine, Nationwide Children's Hospital

Christy Cummings, MD: Associate Professor, Department of Pediatrics, Harvard Medical School, and Division of Newborn Medicine, Boston Children's Hospital

Acknowledgments
The authors wish to acknowledge Leela Sarathy, MD, Sharon Ostfeld-Johns, MD, Armand Antommaria, MD, PhD, Steve Leuthner, MD, MA, and Erin Rholl, MD, MA, who contributed to workshop design and facilitated workshop implementation.

Disclosures
None to report.

Funding/Support
None to report.

Prior Presentations
Ryan KS, Basuray RG, Sarathy L, et al. How can newborn toxicology testing be equitable? Workshop presented at: Pediatric Academic Societies 2023 Meeting; April 28-May 1, 2023; Washington, DC.

Ethical Approval
The Medical College of Wisconsin Institutional Review Board approved this project.

References

1. Patrick SW, Barfield WD, Poindexter BB; Committee on Fetus and Newborn; Committee on Substance Use and Prevention. Neonatal opioid withdrawal syndrome. *Pediatrics*. 2020;146(5):e2020029074. https://doi.org/10.1542/peds.2020-029074
2. Harris M, Schiff DM, Saia K, Muftu S, Standish KF, Wachman EM. Academy of Breastfeeding Medicine clinical protocol #21: breastfeeding in the setting of substance use and substance use disorder (revised 2023). *Breastfeed Med*. 2023;18(10):715-733. https://doi.org/10.1089/bfm.2023.29256.abm
3. Roberts SCM, Nuru-Jeter A. Universal screening for alcohol and drug use and racial disparities in child protective services reporting. *J Behav Health Serv Res*. 2012;39(1):3-16. https://doi.org/10.1007/s11414-011-9247-x
4. American Society of Addiction Medicine Committee on Obstetric Practice. Committee opinion no. 711: opioid use and opioid use disorder in pregnancy. *Obstet Gynecol*. 2017;130(2):e81-e94. https://doi.org/10.1097/AOG.0000000000002235
5. Presler C. Mutual deference between hospitals and courts: how mandated reporting from medical providers harms families. *Columbia J Race Law*. 2021;11(3):733-766. https://doi.org/10.52214/cjrl.v11i3.8750
6. Comprehensive Addiction and Recovery Act, Pub L No. 114-198, 130 Stat 695 (2016).
7. Jensen NC, Cocks M, Brintz BJ, Stoff B, Cipriano SD. Structured case-based ethics discussion for trainees and faculty on dermatopathology. *MedEdPORTAL*. 2023;19:11314. https://doi.org/10.15766/mep_2374-8265.11314
8. Gaffney MM, Cottingham AH. Four medical ethics team-based learning modules. *MedEdPORTAL*. 2012;8:9268. https://doi.org/10.15766/mep_2374-8265.9268
9. Gregg A, Allen W, Black E, Davidson R, McCormack W. An interdisciplinary team-based learning experience in clinical ethics. *MedEdPORTAL*. 2013;9:9579. https://doi.org/10.15766/mep_2374-8265.9579
10. Barkley L, Alford D. Medical ethics and health equity: the Henrietta Lacks story. *MedEdPORTAL*. 2015;11:10276. https://doi.org/10.15766/mep_2374-8265.10276
11. Wynia MK. Professional civil disobedience—medical-society responsibilities after *Dobbs*. *N Engl J Med*. 2022;387(11):959-961. https://doi.org/10.1056/NEJMp2210192
12. Understanding the opioid overdose epidemic. Centers for Disease Control and Prevention. April 5, 2024. Accessed August 8, 2024. https://www.cdc.gov/overdose-prevention/about/understanding-the-opioid-overdose-epidemic.html
13. Beauchamp TL, Childress JF. *Principles of Biomedical Ethics*. Oxford University Press; 1979.
14. Mercer N, Howe C. Explaining the dialogic processes of teaching and learning: the value and potential of sociocultural theory. *Learn Cult Soc Interact*. 2012;1(1):12-21. https://doi.org/10.1016/j.lcsi.2012.03.001
15. Kirkpatrick JD, Kirkpatrick WK. *Kirkpatrick's Four Levels of Training Evaluation*. ATD Press; 2016.
16. Moreau KA. Has the new Kirkpatrick generation built a better hammer for our evaluation toolbox? *Med Teach*. 2017;39(9):999-1001. https://doi.org/10.1080/0142159X.2017.1337874
17. Lucero KS. Net promoter score (NPS): what does net promoter score offer in the evaluation of continuing medical education? *J Eur CME.* 2022;11(1):2152941. https://doi.org/10.1080/21614083.2022.2152941

Received: September 21, 2023
Accepted: April 24, 2024
Published: September 10, 2024

Copyright © 2024 Ryan et al. This is an open-access publication distributed under the terms of the Creative Commons Attribution license.
